# Second generation β-elemene nitric oxide derivatives with reasonable linkers: potential hybrids against malignant brain glioma

**DOI:** 10.1080/14756366.2021.2016734

**Published:** 2022-01-10

**Authors:** Renren Bai, Junlong Zhu, Ziqiang Bai, Qing Mao, Yingqian Zhang, Zi Hui, Xinyu Luo, Xiang-Yang Ye, Tian Xie

**Affiliations:** a School of Pharmacy, Hangzhou Normal University, Hangzhou, Zhejiang, China; b Key Laboratory of Elemene Class Anti-Cancer Chinese Medicine of Zhejiang Province, Engineering Laboratory of Development and Application of Traditional Chinese Medicine from Zhejiang Province, Collaborative Innovation Center of Chinese Medicines from Zhejiang Province, Hangzhou Normal University, Hangzhou, China

**Keywords:** β-Elemene, NO donor, natural product, anti-tumour, malignant glioma

## Abstract

Elemene is a second-line broad-spectrum anti-tumour drug that has been used in China for more than two decades. However, its main anti-tumour ingredient, β-elemene, has disadvantages, including excessive lipophilicity and relatively weak anti-tumour efficacy. To improve the anti-tumour activity of β-elemene, based on its minor molecular weight character, we introduced furoxan nitric oxide (NO) donors into the β-elemene structure and designed six series of new generation β-elemene NO donor hybrids. The synthesised compounds could effectively release NO *in vitro*, displayed significant anti-proliferative effects on U87MG, NCI-H520, and SW620 cell lines. In the orthotopic glioma model, compound **Id** significantly and continuously suppressed the growth of gliomas in nude mice, and the brain glioma of the treatment group was markedly inhibited (>90%). In short, the structural fusion design of NO donor and β-elemene is a feasible strategy to improve the *in vivo* anti-tumour activity of β-elemene.

## 1. Introduction

As an endogenous gas signal molecule, nitric oxide (NO) plays various positive biological and therapeutic effects^1–3^. For example, nitrate type NO donors are frequently applied in the discovery of cardiovascular hybrid drugs. Representative drugs, nitroglycerine and isosorbide dinitrate, can release nitric oxide in the body to exert vasodilation effects^4^. Another critical role of nitric oxide is in the field of anti-tumour drug discovery and development. Low concentrations of NO provide prosurvival effects and high concentrations can induce tumour cell apoptosis^5^. Furoxan NO donors are a classic type of NO donors that release high NO levels both *in vitro* and *in vivo*, leading to the growth inhibition and apoptosis of tumour cells^6–8^. Althrough the introduction of furoxan nitric oxide donors to the parent structure, the anti-tumour activities of the obtained derivatives are often significantly improved, even increasing thousands of times^1^.

**Figure 1.**
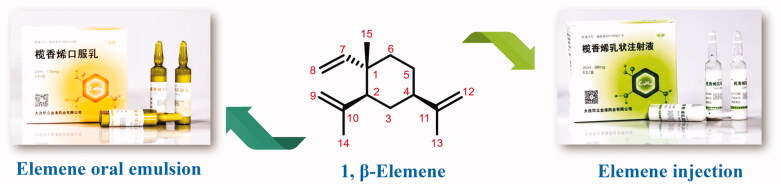
The structure of β-elemene and its clinical preparations.

Natural products have always been a significant direct and indirect source of anti-tumour drugs^9^. Elemene, an anti-tumour natural medicine, is a crucial example. Elemene is a sesquiterpene exacted from the rhizome of *Curcuma wenyujin*. β-Elemene is the highest content in elemene crude extract and is also the most important anti-tumour active ingredient^10^^,^^11^. The anti-tumour mechanisms of β-elemene are relatively complicated. It exerts a good anti-tumour effect in the human body through comprehensive mechanisms, including inhibition of tumour cell growth and proliferation, apoptosis, blocking tumour cell invasion and metastasis, and immune system regulation. More importantly, in human clinical trials, β-elemene also enhances the sensitivitsy of chemotherapy or radiotherapy and reverses multidrug resistance (MDR) ^7^^,^^9^^,^^12–15^. After years of experimentation and efforts, elemene oral emulsion (CFDA number H20010338) and elemene injection (CFDA number H10960114) developed by our research team was approved by the China Food and Drug Administration (CFDA) as broad-spectrum anti-tumour drugs in the 1990s^7^ Figure 1.

Although elemene has achieved good clinical effects and feedbacks, the structure of its main active ingredient β-elemene has certain defects. The molecular formula of β-elemene is C_15_H_24_, which means there are only carbon and hydrogen elements in the structure. Therefore, β-elemene is a volatile oil that is insoluble in water and can only be formulated in liposome formats. In addition, its anti-tumour activity is moderate, and its anti-proliferative half-maximal inhibitory concentrations (IC_50_) against tumour cells are primarily in the range of 200–400 μM. Therefore, it is necessary to carry out purposeful structural modifications to improve its physical and chemical properties on the one hand and enhance its anti-tumour effect on the other.

Based on our experience in the structural modification and optimisation of natural products, strategies from three aspects are generally adopted according to the different molecular weights (MWs) of natural products. For compounds with MWs of less than 300, preferentially introducing other active pharmacophores into their structure is a wise choice; for natural products with MWs of 300–500, it generally tends to synthesise their structural analogues; and in terms of natural products with MWs of more than 500, a better strategy is to simplify their key structures.

Going back to β-elemene, its MW is only 204.4. Therefore, the introduction of other anti-tumour pharmacophores to the β-elemene structure is a preferable and effective strategy. The promising and potent anti-tumour effects of furoxan NO donors prompted us to design and search for the second generation of NO donor β-elemene hybrids. Herein, we reported the design, synthesis, and anti-tumour evaluation of the novel β-elemene nitric oxide derivatives.

## 2. Results and discussion

### 2.1. Design of the β-elemene NO derivatives

**Figure 2.**
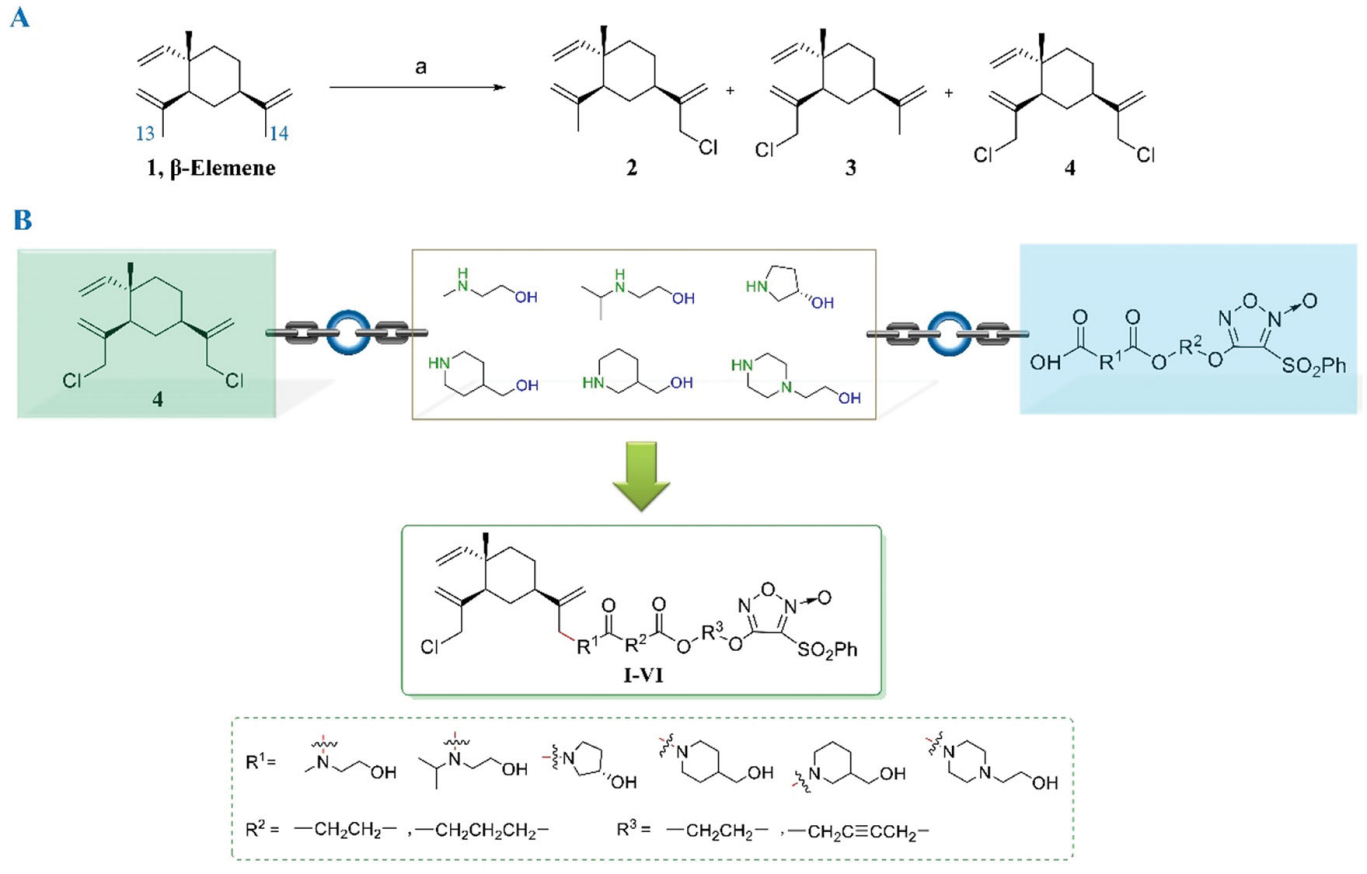
(A) The allylicchlorination reaction of β-elemene and the corresponding reaction products **2–4**. (B) The designing strategy of β-elemene NO donor hybrids.

In the process of structural modification of β-elemene, it is essential to prepare the 13-chloro-β-elemene (**2**) first, which is then applied as a critical intermediate and starting material for subsequent reactions. As shown in Figure 2, in the chlorination reaction, 13-chloro-β-elemene, 14-chloro-β-elemene (**3**), and 13,14-dichloro-β-elemene (**4**) are simultaneously produced. Although 13-chloro-β-elemene is the main product, the polarities of the monosubstituted chlorinated derivatives 13-chloro-β-elemene and 14-chloro-β-elemene are too similar to separate. Even if being prepared by HPLC, it is still challenging to obtain baseline separation. Their mixture can only be used as the raw material for subsequent reactions, resulting in forming a certain amount of the 14-substituted derivatives in the final products, increasing the complexity in purification.

However, high purified 13,14-dichloro-β-elemene is relatively easier to obtain since its polarity is much stronger than that of monochloro-β-elemenes. In addition, the anti-tumour activity of 13,14-dichloro-β-elemene is almost equivalent to that of 13-chloro-β-elemene, 14-chloro-β-elemene, and β-elemene. Therefore, 13,14-dichloro-β-elemene was selected as the core structure for further structural modification in the manuscript.

**Figure 3.**
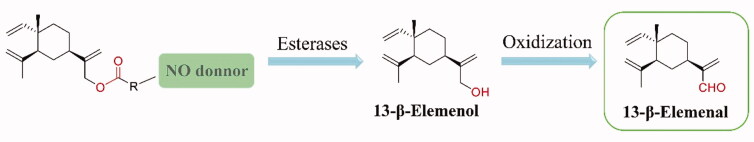
*In vivo* metabolizion of 13-β-elemenol ester NO donor derivatives may result in producing toxic 13-β-elemenal.

Another important task is to choose a suitable linker to combine β-elemene and furazan NO donors. The author Bai’s former research group used 13-β-elemenol as a key intermediate and connected it with furoxan nitric oxide donors *via* esterification reaction affording a series of ester nitric oxide β-elemene derivatives (Figure 3). Although this series of compounds showed well *in vitro* anti-tumour activity and *in vivo* anti-tumour effects, the ester group at 13-position is quickly metabolised into 13-β-elemenol by esterase *in vivo*. Subsequently, 13-β-elemenol will be rapidly oxidised and metabolised to 13-β-elemenal, which exhibits potent cytotoxicity, resulting in a greater safety risk under long-term medication^8^.

Based on years of experience in the structural modification of β-elemene, it is found that the introduction of a nitrogen-containing group at 13-position significantly increases the anti-proliferative activity. Therefore, alcohol amine structures, containing both amino and alcoholic hydroxyl groups, were chosen as the linkers (Figure 2(B)), in which the amino group was reacted with 13-chloro of β-elemene, and the alcoholic hydroxyl group was connected to furazan moieties. Therefore, such linkers not only play the role of linking chains but also help to improve the overall anti-tumour activity and decrease the cytotoxicity of β-elemene derivatives. In summary, the 13,14-dichloro-β-elemene and furzan NO donors were combined using the alcohol amine structures as the linkers, and six series of novel generation β-elemene NO derivatives were designed and synthesised.

### 2.2. Chemistry

**Scheme 1.**
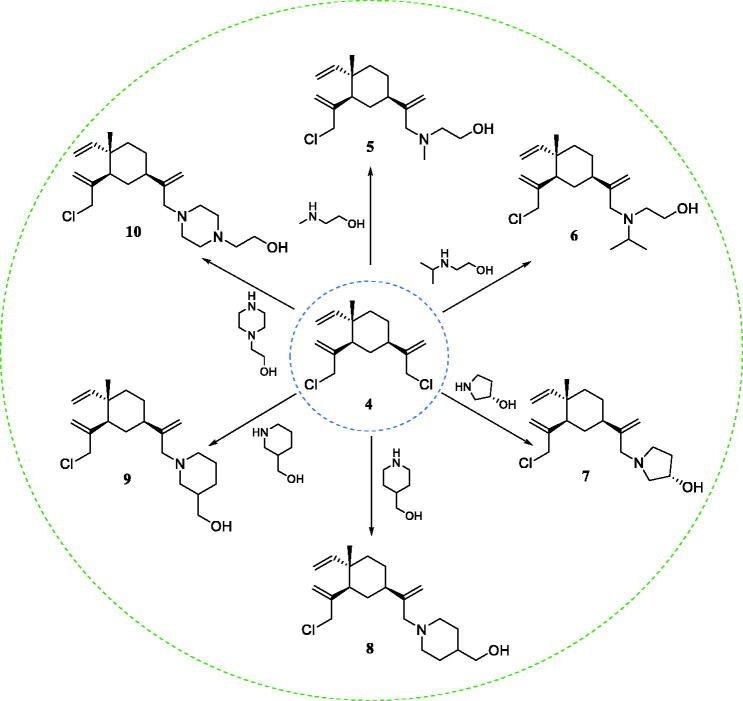
Reagents and conditions: DIPEA, DMF, 60 °C.

The synthetic route to prepare β-elemene key intermediates is shown in Scheme 1. Chlorination of β-elemene (**1**) with *N*-chlorosuccinimide (NCS) produced the chlorinated mixture of **2**, **3**, and **4**, which was quickly passed through the column to obtain intermediate **4**. Then, the reaction of **4** with different alcohol amines afforded key intermediates **5**–**10**.

**Scheme 2.**
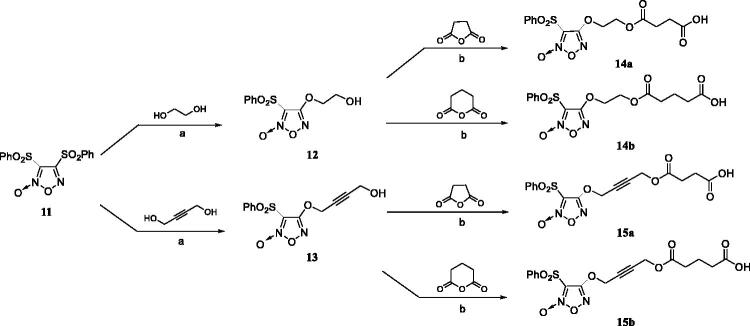
Reagents and conditions: (a) THF, NaOH (4:1, v/v), r.t.; (b) DMAP, DCM, r.t.

The synthetic method of furazan NO donors is illustrated in Scheme 2. The key intermediate **11** was prepared according to reported methods^8^. The substituted furoxan derivatives **14a–b** and **15a–b** were synthesised in a two-step sequence. Compounds **12** and **13** were prepared *via* hydroxylation of the starting material **11** with the corresponding disubstituted alcohols in THF. Then, compounds **12** and **13** were converted to key intermediates **14a–b** or **15a–b** by treatment with corresponding anhydrides in the presence of DMAP.

**Scheme 3.**
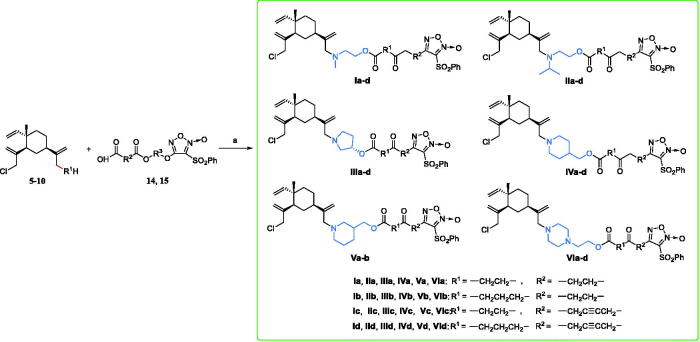
Reagents and conditions: EDCI, DMAP, DCM, r.t.

The synthetic route chosen to synthesise the designed hybrids is outlined in Scheme 3. Amidation reaction of β-elemene intermediates **5–10** with furazan intermediates **14a–b** or **15a–b** under the condition of DMAP and EDCI, respectively, to obtain the final NO-donating β-elemene derivatives **Ia–d**, **IIa–d**, **IIIa–d**, **Iva–d**, **Via–d**, **Va–d,** and **Via–d**.

### 2.3. NO-releasing test

Since the designing strategy of the compounds is to improve the anti-tumour activity by introducing NO donor, the NO release ability and NO release level of the compounds are essential properties.^16^ Through the *in vitro* NO release test of all the prepared compounds, it was found that with time increasing, the NO release levels of all compounds showed an overall increasing trend (Table 1). After 30 min, the NO release levels of most compounds began to rise significantly. Among them, the NO release levels of compounds **Id**, **IIId**, **IVd,** and **Vd** are relatively higher than others. However, compounds **Ia**, **Ib**, **IIa**, **VIa**, **VIb,** and **VIc** were lower, and the NO levels were <20 μM at 120 min. In general, the *in vitro* NO release levels of compounds with a 1,4-butynediol linker are higher than those with an ethylene glycol linker. Moreover, the *in vitro* NO release levels of compounds possessing a glutaric anhydride as the linking group is higher than that of compounds with a succinic anhydride as the linking group. In summary, all the compounds can effectively release NO, which will be helpful to improve the anti-tumour activity.

**Table 1.** NO-release concentrations of β-elemene derivatives at 100 μM.

Compounds	Concentrations of NO at seven-time points (Mean ± SD, μM)						
	5 min	10 min	20 min	30 min	60 min	90 min	120 min
**Ia**	1.73 ± 0.06	2.31 ± 0.09	2.90 ± 0.41	4.04 ± 0.05	7.76 ± 0.18	12.67 ± 0.59	17.21 ± 0.35
**Ib**	2.10 ± 0.20	2.40 ± 0.02	2.84 ± 0.10	3.79 ± 0.11	6.74 ± 0.30	10.72 ± 1.00	14.97 ± 0.59
**Ic**	10.78 ± 0.10	12.02 ± 0.64	15.83 ± 0.43	19.79 ± 0.14	32.99 ± 0.56	48.39 ± 1.26	58.84 ± 0.44
**Id**	12.53 ± 0.08	14.80 ± 0.26	19.55 ± 0.48	23.65 ± 0.92	42.31 ± 0.79	66.97 ± 1.53	93.18 ± 0.50
**IIa**	0.59 ± 0.08	41.61 ± 1.28	48.58 ± 1.54	2.57 ± 0.02	5.58 ± 1.25	9.22 ± 0.56	13.64 ± 0.63
**IIb**	3.38 ± 0.28	5.87 ± 2.84	4.62 ± 0.19	6.22 ± 0.14	9.97 ± 0.33	15.53 ± 0.86	20.60 ± 0.54
**IIc**	5.68 ± 0.07	7.17 ± 0.63	9.51 ± 1.00	9.04 ± 2.53	20.74 ± 0.44	30.04 ± 1.34	40.44 ± 0.43
**IId**	10.55 ± 0.19	12.34 ± 0.72	15.52 ± 0.79	18.95 ± 1.01	29.50 ± 0.79	43.00 ± 0.72	55.33 ± 0.46
**IIIa**	3.25 ± 0.12	3.90 ± 0.41	5.98 ± 0.57	7.59 ± 0.45	11.11 ± 0.74	17.29 ± 0.29	23.48 ± 0.27
**IIIb**	8.39 ± 0.10	9.44 ± 0.29	10.65 ± 0.63	12.22 ± 0.31	18.15 ± 0.56	26.49 ± 0.05	34.97 ± 0.06
**IIIc**	9.49 ± 0.25	10.79 ± 1.24	14.43 ± 0.77	15.97 ± 1.48	24.58 ± 1.10	37.37 ± 0.53	50.99 ± 0.01
**IIId**	13.20 ± 0.43	16.04 ± 0.20	21.29 ± 0.79	26.90 ± 1.11	46.58 ± 1.17	74.45 ± 1.89	103.92 ± 0.53
**IVa**	11.09 ± 0.11	12.18 ± 0.07	14.69 ± 0.10	17.14 ± 0.10	25.80 ± 0.16	38.78 ± 0.29	51.94 ± 0.62
**IVb**	12.34 ± 0.04	13.53 ± 0.64	15.94 ± 0.14	18.96 ± 0.07	29.94 ± 0.90	45.42 ± 1.59	62.49 ± 1.97
**IVc**	8.67 ± 0.13	10.07 ± 0.61	12.98 ± 0.23	15.92 ± 0.25	25.47 ± 1.41	36.31 ± 0.96	48.15 ± 1.23
**IVd**	16.97 ± 0.47	18.93 ± 0.28	22.62 ± 0.03	27.04 ± 0.13	42.47 ± 0.156	64.24 ± 0.92	86.29 ± 1.33
**Va**	4.84 ± 0.22	5.66 ± 0.33	6.95 ± 0.17	8.44 ± 0.17	12.43 ± 0.20	17.81 ± 0.41	22.91 ± 0.58
**Vb**	8.89 ± 0.05	10.18 ± 0.28	12.42 ± 0.92	14.32 ± 0.56	21.50 ± 0.89	32.40 ± 1.44	42.83 ± 1.22
**Vc**	13.76 ± 0.60	15.87 ± 0.01	19.21 ± 0.43	22.88 ± 0.45	34.60 ± 0.88	48.73 ± 0.61	56.52 ± 1.58
**Vd**	15.19 ± 0.20	17.57 ± 0.96	21.31 ± 0.45	25.52 ± 0.79	39.29 ± 1.17	58.12 ± 1.32	77.28 ± 1.14
**VIa**	0.08 ± 0.04	0.41 ± 0.22	0.99 ± 0.36	1.64 ± 0.43	2.98 ± 0.48	6.43 ± 0.07	10.20 ± 0.17
**VIb**	0.33 ± 0.13	0.89 ± 0.07	1.35 ± 0.00	1.91 ± 0.16	4.05 ± 0.36	7.20 ± 0.21	10.51 ± 0.29
**VIc**	4.74 ± 0.07	5.95 ± 0.59	7.49 ± 1.22	7.63 ± 0.88	8.44 ± 1.76	13.57 ± 0.66	17.82 ± 1.74
VId	10.64 ± 0.07	11.37 ± 0.63	12.38 ± 0.15	13.68 ± 0.36	18.24 ± 0.17	25.90 ± 0.28	34.64 ± 0.06

### 2.3. *In vitro* anti-tumour activity

The *in vitro* anti-tumour activities of all the compounds against U87MG (malignant glioma cells), NCI-H520 (lung cancer cells), SW620 (colon cancer cells) tumour cell lines were first preliminary screened at the concentration of 1 μM. Their antiproliferative activities were significantly more potent than the positive control β-elemene and 13, 14-dichloro-β-elemene, demonstrating that the introduction of NO donors has successfully enhanced the anti-tumour effect of β-elemene (Table 2).

**Table 2.** Proliferation inhibition of β-elemene and β-elemene derivatives at 1 μM.

Compounds	Inhibitory rate ± SEM (%, 1 μM)		
	SW620	U87MG	NCI-H520
**β-elemene**	2.5 ± 4.1	4.3 ± 2.2	3.2 ± 0.7
**13,14-bischloro-β-elemene**	3.3 ± 1.0	3.5 ± 1.0	0.9 ± 3.0
**Ia**	30.7 ± 3.5	39.5 ± 2.1	28.8 ± 0.8
**Ib**	42.3 ± 3.7	56.0 ± 2.1	48.5 ± 3.0
**Ic**	30.1 ± 5.2	34.9 ± 0.6	35.0 ± 2.2
**Id**	47.5 ± 3.7	55.5 ± 1.9	48.7 ± 2.0
**IIa**	33.0 ± 2.5	47.1 ± 0.9	44.2 ± 1.3
**IIb**	35.6 ± 2.8	54.3 ± 1.3	43.4 ± 1.5
**IIc**	46.6 ± 1.2	56.7 ± 0.9	44.5 ± 2.2
**IId**	10.8 ± 3.2	4.5 ± 1.0	5.6 ± 1.6
**IIIa**	39.3 ± 4.2	51.3 ± 1.3	44.5 ± 2.8
**IIIb**	45.9 ± 3.9	53.1 ± 1.4	46.7 ± 2.0
**IIIc**	30.5 ± 3.3	42.0 ± 6.5	46.0 ± 1.0
**IIId**	31.7 ± 1.4	47.8 ± 1.1	39.1 ± 0.7
**IVa**	53.6 ± 2.4	52.6 ± 1.7	48.5 ± 2.5
**IVb**	55.4 ± 1.5	54.8 ± 1.9	50.1 ± 1.1
**IVc**	31.3 ± 0.3	40.5 ± 3.3	45.9 ± 1.4
**IVd**	39.8 ± 1.9	52.2 ± 3.4	52.3 ± 1.1
Va	38.3 ± 3.0	51.0 ± 2.2	45.4 ± 1.9
Vb	46.1 ± 0.9	48.3 ± 1.5	43.9 ± 1.0
**Vc**	40.6 ± 2.8	41.9 ± 1.1	46.1 ± 2.7
**Vd**	43.2 ± 1.8	53.9 ± 0.6	44.5 ± 2.4
**VIa**	43.6 ± 2.5	52.6 ± 1.0	47.3 ± 2.6
**VIb**	42.8 ± 0.5	52.4 ± 2.0	44.1 ± 2.4
**VIc**	21.2 ± 2.1	33.4 ± 1.5	30.3 ± 2.9
**VId**	43.2 ± 5.5	50.3 ± 1.2	46.8 ± 2.1

Subsequently, the compounds with an inhibition greater than 40% at the concentration of 1 μM were selected to further test their IC_50_ values against these three tumour cell lines. As illustrated in Table 3, the anti-proliferative activities of most of the compounds are absolutely more potent than β-elemene and 13,14-dichloro-β-elemene. For SW620 cell line, compounds **Id** and **IVb** are over 110-fold more active than β-elemene; for U87MG cell line, compounds **Ib**, **Id**, **IIa**, **IIb**, **IIIa**, **IIIb,** and **VIb** displayed over 250-fold more potent than β-elemene; in terms of NCI-H520 cell line, the activity of compounds **Ib**, **Id**, **IIIb**, **IVb,** and **VIb** is over 100-fold greater than β-elemene.

**Table 3.** IC_50_ values of compounds against threes tumour cell lines.

Compounds	IC_50_ ± SEM (μM)		
	SW620	U87MG	NCI-H520
**β-elemene**	>100	>100	>100
**13,14-bischloro-β-elemene**	>100	>100	>100
**Ib**	–	0.358 ± 0.023	0.777 ± 0.024
**Id**	0.858 ± 0.033	0.369 ± 0.013	0.719 ± 0.017
**IIa**	–	0.369 ± 0.025	–
**IIb**	–	0.366 ± 0.019	–
**IIc**	1.111 ± 0.115	0.882 ± 0.034	–
**IIIa**	–	0.323 ± 0.013	–
**IIIb**	1.022 ± 0.09	0.343 ± 0.029	0.848 ± 0.053
**IIIc**	–	–	1.019 ± 0.041
**IIId**	–	0.898 ± 0.054	–
**IVa**	1.069 ± 0.101	1.119 ± 0.022	1.301 ± 0.043
**IVb**	0.814 ± 0.068	0.477 ± 0.011	0.846 ± 0.027
**IVc**	–	–	1.038 ± 0.033
**IVd**	–	2.160 ± 0.548	1.046 ± 0.013
**Va**	1.088 ± 0.04	0.811 ± 0.016	–
**Vb**	–	–	1.212 ± 0.152
**Vc**	–	0.985 ± 0.012	–
**Vd**	–	1.114 ± 0.052	1.175 ± 0.066
**VIa**	–	0.926 ± 0.052	–
**VIb**	–	0.372 ± 0.006	0.962 ± 0.054
**VId**	–	0.960 ± 0.075	1.062 ± 0.002

According to the *in vitro* NO release experiment and the anti-tumour activity screening of the synthesised NO donor β-elemene derivatives, the following conclusions can be drawn: (1) The synthesised NO donor β-elemene derivatives exerted remarkably improved *in vitro* anti-tumour activity. Relatively speaking, the designed compound demonstrated better sensitivity to U87MG; (2) It is a feasible strategy to apply alcohol amine structures as the linkers. Among them, the compounds with *N*-methyl-2-hydroxyethylamine, 2-(isopropylamino)ethanol and (*R*)-3-pyrrolidinol as the linkers exhibited greater activity overall (e.g. **Ib**, **Id**, **IIa**, **IIb**, **IIIa**, and **IIIb**); (3) The activity of compounds with ethylene glycols as the furoxan linker is generally more potent than that of 1,4-butynediol, and the compounds with glutarates as the transition side chain are more active than those with succinates (e.g. **Ib**>**Id**, **IIb**>**IIa**>**IIc**, **IVb**>**IVa**>**IVd**, **VIb**>**VIa**>**VId**).

An additional MTT assay was also performed to test the tumour cell selectivity and cytotoxicity of candidate compound **Id**. The human lung fibroblast HFL-1 cell, a normal line, was selected to evaluate the antiproliferative activity of compound **Id**. The IC_50_ value of **Id** against HFL-1 cell line was 1.957 μM, while its IC_50_ values against SW620, U87MG and NCI-H520 cell lines were 0.856, 0.369 and 0.716 μM, respectively. Therefore, compound **Id** showed slight selectivity to tumour cells, but more assays need to be performed to verify its selectivity.

### 2.3. *In vivo* anti-tumour activity against malignant brain glioma

Malignant glioma is one of the most common malignant brain tumours. Despite intensive treatment by surgery, radiation, and chemotherapy, the prognosis for malignant glioma is still very poor, and the median survival is only about 15 months.^17^ Therefore, there is an urgent need to discover more effective drugs. Since compound **Id** exhibited a broad-spectrum anti-tumour activity and showed a markable anti-proliferative effect on U87MG cells, it was selected as the candidate for further *in vivo* anti-tumour test against malignant brain glioma in the orthotopic glioma model.

**Figure 4.**
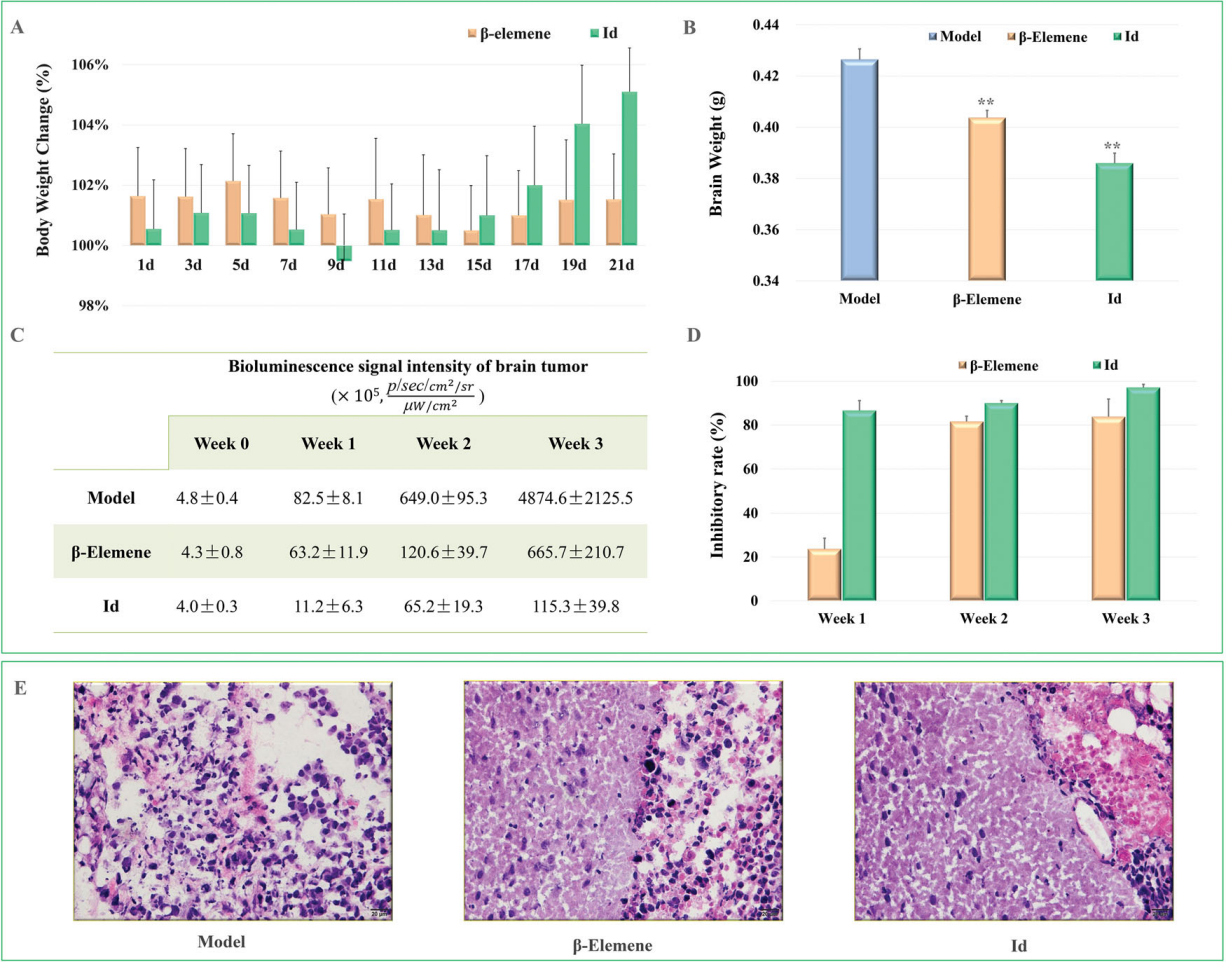
The anti-tumour activity of β-elemene and compound **Id** against brain malignant glioma (*n* = 5). (A) Body weight (model group as 100%). (B) Brain weight. (C) Boluminescence signal intensity of brain tumour. (D) Inhibitory rates of β-elemene and compound **Id** from week 1 to week 3. (E) Representative brain glioma images of the model, β-elemene, and compound **Id** groups. (F) Histological analysis of the brain glioma tumour tissue (**P < 0.01 vs. model group; ^##^*P* < 0.01 vs. β-elemene group).

As shown in Figure 4(A), during the three weeks treatment, the mouse bodyweight of the β-elemene and **Id** groups all increased slightly, indicating that both β-elemene and compound **Id** did not exhibit observed toxicity. More importantly, the tumour volume in the model group continued to increase, but the tumour growth was effectively inhibited after treatment with β-elemene and compound **Id**. The brain weight of β-elemene and compound **Id** groups was markedly lighter than that of the model group (Figure 4(B)), and the living and physical conditions of mice were much better than the model groups. On the other hand, the bioluminescence signal intensity of gliomas in the model group continued to increase rapidly, but the signal intensity declined dramatically in β-elemene and compound **Id** groups (Figure 4(C)). From the point of view of inhibitory rate (Figure 4(D)), the inhibitory activity of compound **Id** exceeded 80% in the first week, which was remarkably more potent than that of β-elemene (>20%). In the second week, both β-elemene and the compound **Id** reached >80% inhibition. In the last week of administration, compound **Id** blocked the tumour growth by >90% (β-elemene: >80%), displaying potent anti-glioma activity. The histological analysis indicated that the infiltration of malignant glioma tissue was inhibited, and the necrotic area was significantly reduced (Figure 4(E)).

In summary, both β-elemene and the compound **Id** displayed effective therapeutic activity, but the compound **Id** showed a more significant inhibition than that of β-elemene. The above results indicate that the introduction of NO donors effectively enhances the anti-tumour effect of β-elemene *in vivo*, and is a feasible strategy for the structural modification of β-elemene in further investigation.

## 3. Discussion

The designed second-generation β-elemene NO derivatives exhibited promising anti-tumour activity both *in vitro* and *in vivo*. However, more in-depth studies are needed to further evaluate their drug-like properties and mechanism of action. Firstly, the structures of the NO donor compounds contain several unstable groups, and the *in vivo* stability of the preferred compound needs to be further tested. Secondly, it is necessary to study the tissue targeting of the compounds. It is interesting and essential to determine whether the compound releases NO after passing through the blood-brain barrier (BBB) and then inhibits the growth of glioma, or whether it has been decomposed to release the NO donor already before passing the BBB, and then the NO donor pass the BBB and exerts the anti-tumour effect. Moreover, the influence of the number of NO donors in β-elemene derivatives on the activity is also worthy of in-depth studies, such as if the introduction of NO donors at both positions 13 and 14 will further improve the anti-tumour activity.

## 4. Conclusion

Natural products are an important source of anti-tumour drugs, and it is of great significance to carry out structural modification and optimisation to enhance the activities and improve their physicochemical properties. To improve the structural property limitations and the anti-tumour activity of β-elemene, we selected various alcohol amine structures as linkers to successfully introduce furoxan NO donors to the C-13 of β-elemene and obtained a series of hybrids with remarkable increased anti-tumour activities both *in vitro* and *in vivo*. The candidate compound **Id** not only exerted marked anti-tumour effects *in vitro* but also significantly suppressed the growth of malignant gliomas in the orthotopic glioma model, achieving a desired therapeutic effect during the three weeks’ administration (>90% inhibition). Subsequent experiments will be mainly focussed on the investigation of the anti-tumour effects of other reasonable linkers and different types of NO donors, and further test the selectivity and specificity of β-elemene NO donor hybrids on various tumours.

## Supplementary Material

Supplemental Material
