# Supplementary material for: Second generation β-elemene nitric oxide derivatives with reasonable linkers: potential hybrids against malignant brain glioma
Source: J Enzyme Inhib Med Chem. 2022 Jan 10;37(1):379–85. doi: 10.1080/14756366.2021.2016734 (PMC8757613; doi:10.1080/14756366.2021.2016734)

Supporting Information

Second generation β-elemene nitric oxide derivatives with reasonable linkers: potential hybrids against malignant brain glioma

Renren Bai [a, b, *], Junlong Zhu [a, b], Ziqiang Bai [a, b], Qing Mao [a, b], Yingqian Zhang [a, b], Zi Hui [a, b], Xinyu Luo [a, b], Xiang-Yang Ye [a, b, *], Tian Xie [a, b, *]

[a]*School of Pharmacy, Hangzhou Normal University, Hangzhou, Zhejiang 311121, PR China.*

[b]*Key Laboratory of Elemene Class Anti-Cancer Chinese Medicine of Zhejiang Province, Engineering Laboratory of Development and Application of Traditional Chinese Medicine from Zhejiang Province, Collaborative Innovation Center of Chinese Medicines from Zhejiang Province, Hangzhou Normal University, Hangzhou, PR China.*

Contents

1. Chemistry

   1.1. General information

   1.2. General synthetic procedures for **4**

   1.3. General synthetic procedures for **5-10**

   1.4. General synthetic procedure for **11**

   1.5. General synthetic procedures for **12** and **13**

   1.6. General synthetic procedures for **14a-b**

   1.7. General synthetic procedures for **15a-b**

   1.8. General synthetic procedures for Ia-d, IIa-b, IIIa-d, IVa-d, Va-b, and VIa-b

2. NO-releasing test

3. Anti-proliferative assay

4. U87MG orthotopic glioma model

5. 1H and 13C NMR spectra of compounds **Ia-d**, **IIa-b**, **IIIa-d**, **IVa-d**, **Va-b**, and **VIa-b**

1. Chemistry

1.1. General information

All of the chemical materials were purchased from commercial suppliers. The melting points of the compounds were determined using Bϋchi B-540 capillary melting point instrument. NMR spectra were recorded on a Bruker instrument at 400 MHz for ^{1}H NMR and 100 MHz for ^{13}C NMR, using $CDCl_3$ as the deuterated solvent. Chemical shifts(δ) are reported in parts per million (ppm) relative to residual solvent as an internal reference. Mass spectra were recorded with Thermo Finnigan LCQ-Advantage in positive polarity. High-resolution mass spectra (HRMS) were measured on a Bruker MICR OTOF-Q II instrument or Shimadzu LCMSIT-TOF mass spectrometer using ESI technique.

1.2. General synthetic procedures for 4

A solution of β-elemene 1 (1.02 g, 5 mmol) and THF (2 mL) in DCM (8 mL) was stirred for 10 min at 0 ℃. Then, NCS (2.0 g, 15 mmol), ytterbium trifluoromethane sulfonate (310 mg, 0.5 mmol), and chlorotrimethylsilane (TMCS) (54 mg, 0.5 mmol) were added. The reaction mixture was allowed to stir at 0 ℃ for 5 h. Then, the organic phase was evaporated under vacuum; the residue was diluted in H2O (15 mL) and was extracted three times with EA (20 mL). The combined organic layer was sequentially washed with water and brine, dried with anhydrous Na2SO4, and concentrated in vacuo. The residue was purified via column chromatography (pure petroleum ether) and compound 4 (650 mg, 48%) was obtained as a colorlessliquid.

^{1}H NMR (400 MHz, $CDCl_3$) δ 5.85 – 5.72 (m, 1H), 5.28 (s, 1H), 5.18 (s, 1H), 5.04 (s, 1H), 4.98 – 4.89 (m, 3H), 4.15 – 4.05 (m, 3H), 3.97 (d, J = 11.7 Hz, 1H), 2.35 – 2.21 (m, 2H), 1.77 – 1.42 (m, 6H), 0.99 (s, 3H). ^{13}C NMR (100 MHz, $CDCl_3$) δ 149.6, 149.0, 147.5, 116.4, 113.5, 111.6, 51.1, 47.8, 47.5, 41.0, 39.8, 39.7, 33.8, 27.0, 15.8.

1.3. General synthetic procedures for 5-10

The solution of compound **4** (1.1 mmol), DIPEA (1.3 mmol), and the corresponding hydroxyl amine (1.3 mmol) in DMF (2 mL) was stirred at 60 ℃ for 12 h. The reaction was monitored by TLC. The mixture was quenched with water at room temperature and extracted with EA. The organic layer was separated, washed with water and brine, dried over Na_2SO_4, and

concentrated in vacuo. The crude was purified *via* column chromatography (DCM/MeOH 150:1, v/v) to give the products as light yellow liquid.

1.3.1. 2-((2-((1R,3R,4S)-3-(3-chloroprop-1-en-2-yl)-4-methyl-4-vinylcyclohexyl) allyl)(methyl)amino)ethan-1-ol (5)

Light yellow liquid, yield 44%. ^{1}H NMR (400 MHz, $CDCl_3$) δ 5.78 (dd, J = 18.0, 10.3 Hz, 1H), 5.27 (s, 1H), 5.01 – 4.88 (m, 5H), 4.14 – 3.91 (m, 2H), 3.61 (t, J = 5.4 Hz, 2H), 3.01 (s, 2H), 2.56 – 2.51 (m, 2H), 2.28 (dd, J = 12.5, 3.4 Hz, 1H), 2.20 (s, 3H), 2.11 (t, J = 11.8 Hz, 1H), 1.72 – 1.44 (m, 6H), 0.98 (s, 3H). ^{13}C NMR (100 MHz, $CDCl_3$) δ 151.0, 149.3, 147.8, 116.2, 111.4, 111.3, 63.1, 59.0, 58.6, 51.0, 41.9, 41.7, 40.0, 39.9, 34.1, 27.3, 16.0. HRMS (ESI) calcd for $C_{18}H_{31}ClNO$ 312.2089 $[M + H]^+$, found 312.2080.

1.3.2. 2-((2-((1R,3R,4S)-3-(3-chloroprop-1-en-2-yl)-4-methyl-4-vinylcyclohexyl) allyl)(isopropyl)amino)ethan-1-ol (6)

Light yellow liquid, yield 60%. ^{1}H NMR (400 MHz, $CDCl_3$) δ 5.77 (dd, J = 17.1, 11.2 Hz, 1H), 5.26 (s, 1H), 5.02 – 4.79 (m, 5H), 4.02 (dd, J = 47.3, 11.7 Hz, 2H), 3.51 (s, 2H), 3.02 (d, J = 18.7 Hz, 3H), 2.54 (s, 2H), 2.27 (dd, J = 12.6, 3.1 Hz, 1H), 2.14 – 2.02 (m, 1H), 1.71 – 1.41 (m, 6H), 0.98 (d, J = 7.2 Hz, 9H). ^{13}C NMR (100 MHz, $CDCl_3$) δ 149.2, 147.7, 116.3, 111.4, 58.5, 54.9, 51.2, 49.9, 49.1, 47.9, 41.7, 39.9, 40.0, 34.3, 27.3, 17.8, 17.7, 15.9. HRMS (ESI) calcd for $C_{20}H_{35}ClNO$ 340.2402 $[M + H]^+$, found 340.2394.

1.3.3. (S)-1-(2-((1R,3R,4S)-3-(3-chloroprop-1-en-2-yl)-4-methyl-4-vinylcyclohexyl)allyl)pyrrolidin-3-ol (7)

Light yellow liquid, yield 48%. ^{1}H NMR (400 MHz, $CDCl_3$) δ 5.77 (dd, J = 17.1, 11.1 Hz, 1H), 5.26 (s, 1H), 5.05 – 4.80 (m, 5H), 4.30 (s, 1H), 4.09 (d, J = 11.6 Hz, 1H), 3.96 (d, J = 11.7 Hz, 1H), 3.05 (q, J = 13.4 Hz, 2H), 2.85 (td, J = 8.1, 7.2, 4.1 Hz, 1H), 2.66 (d, J = 10.2 Hz, 1H), 2.55 (s, 1H), 2.43 (dd, J = 10.2, 5.1 Hz, 1H), 2.31 – 2.07 (m, 4H), 1.69 – 1.41 (m, 6H), 0.96 (s, 3H). ^{13}C NMR (100 MHz, $CDCl_3$) δ 151.4, 149.3, 147.7, 116.3, 111.3, 110.2, 71.5, 63.1, 60.9, 52.6, 51.1, 47.7, 42.0, 39.9, 39.8, 35.0, 34.0, 27.1, 15.8. HRMS (ESI) calcd for $C_{19}H_{31}ClNO$ 324.2089 $[M + H]^+$, found 324.2083.

1.3.4. (1-(2-((1R,3R,4S)-3-(3-chloroprop-1-en-2-yl)-4-methyl-4-vinylcyclohexyl) allyl)piperidin-4-yl)methanol (8)

Light yellow liquid, yield 60%. ^{1}H NMR (400 MHz, $CDCl_3$) *δ* 5.79 (dd, *J* = 17.9, 10.4 Hz, 1H), 5.26 (s, 1H), 4.98 – 4.86 (m, 5H), 4.09 (d, *J* = 11.7 Hz, 1H), 3.97 (d, *J* = 11.7 Hz, 1H), 3.50 (d, *J* = 6.4 Hz, 2H), 2.90 (d, *J* = 14.5 Hz, 4H), 2.32 – 2.23 (m, 1H), 2.13 (d, *J* = 7.9 Hz, 1H), 1.88 (s, 2H), 1.75 – 1.57 (m, 7H), 1.53 – 1.43 (m, 4H), 0.98 (s, 3H). ^{13}C NMR (100 MHz, $CDCl_3$) *δ* 149.1, 147.6, 115.9, 110.9, 67.8, 63.5, 53.5, 53.3, 50.8, 47.6, 42.0, 39.7, 39.6, 38.5, 33.8, 28.6, 26.8, 15.6. HRMS (ESI) calcd for $C_{21}H_{35}ClNO$ 352.2402 $[M + H]^+$, found 352.2384.

1.3.5. (1-(2-((1R,3R,4S)-3-(3-chloroprop-1-en-2-yl)-4-methyl-4-vinylcyclohexyl) allyl)piperidin-3-yl)methanol (9)

Light yellow liquid, yield 51%. ^{1}H NMR (400 MHz, $CDCl_3$) *δ* 5.83 – 5.73 (m, 1H), 5.26 (s, 1H), 4.91 (dd, *J* = 10.5, 6.4 Hz, 5H), 4.14 – 4.02 (m, 1H), 3.97 (d, *J* = 11.7 Hz, 1H), 3.69 – 3.54 (m, 2H), 2.89 (d, *J* = 3.9 Hz, 2H), 2.68 (d, *J* = 9.7 Hz, 1H), 2.47 (s, 2H), 2.27 (dd, *J* = 11.0, 5.0 Hz, 1H), 2.09 (ddt, *J* = 12.2, 8.2, 4.3 Hz, 2H), 1.82 – 1.74 (m, 2H), 1.70 – 1.41 (m, 9H), 0.97 (s, 3H). ^{13}C NMR (100 MHz, $CDCl_3$) δ 150.7, 149.4, 147.8, 147.8, 116.2, 111.3, 111.0, 67.5, 64.1, 57.8, 54.4, 51.0, 47.8, 42.1, 39.9, 39.9, 34.0, 27.8, 27.0, 24.6, 15.8. HRMS (ESI) calcd for $C_{21}H_{35}ClNO$ 352.2402 $[M + H]^+$, found 352.2412.

1.3.6. 2-(4-(2-((1R,3R,4S)-3-(3-chloroprop-1-en-2-yl)-4-methyl-4-vinylcyclohexyl) allyl)piperazin-1-yl)ethan-1-ol (10)

Light yellow liquid, yield 44%. ^{1}H NMR (400 MHz, $CDCl_3$) *δ* 5.78 (dd, *J* = 18.0, 10.2 Hz, 1H), 5.26 (s, 1H), 4.97 – 4.85 (m, 5H), 4.09 (d, *J* = 11.5 Hz, 1H), 3.96 (d, *J* = 11.6 Hz, 1H), 3.61 (t, *J* = 5.4 Hz, 2H), 2.91 (d, *J* = 4.2 Hz, 2H), 2.62 – 2.38 (m, 10H), 2.30 – 2.25 (m, 1H), 2.15 – 2.08 (m, 1H), 1.64 – 1.43 (m, 6H), 0.97 (s, 3H). ^{13}C NMR (100 MHz, $CDCl_3$) *δ* 150.7, 149.4, 147.8, 116.3, 111.3, 111.0, 63.5, 59.4, 57.8, 53.3, 53.1, 51.2, 47.7, 42.2, 39.9, 39.9, 34.0, 27.1, 15.8. HRMS (ESI) calcd for $C_{21}H_{36}ClN_2O$ 367.2511 $[M + H]^+$, found 367.2503.

1.4. General synthetic procedure for 11

To a solution of 2-(phenylthio)ethan-1-ol (4.75 g, 28 mmol) in ethanoic acid was added dropwise hydrogen peroxide (7 mL, 30% w/w) at 0 °C. After addition, the mixture was stirred at room temperature for 5-6 h. Nitric acid (12.5 mL, 98% w/w) was then added dropwise to the mixture at 0 °C, and the mixture was stirred at 140 °C for 6-8 h. The reaction was monitored by TLC. After completion, the reaction mixture was kept to 0 °C and washed with water thrice after a Büchner filtration. The filter cake was dried without further purification to give **11** (2 g, yield 40%), as a white crystal, m.p. 157-159 °C. ^{1}H NMR (400 MHz, $CDCl_3$) δ 8.17 (t, J = 7.3 Hz, 4H), 7.81 (q, J = 7.5 Hz, 2H), 7.70 – 7.62 (m, 4H). ^{13}C NMR (100 MHz, $CDCl_3$) δ 155.7, 137.2, 136.4, 136.3, 136.0, 130.3, 130.0, 129.7, 129.3, 115.3. HRMS (ESI) calcd for $C_{14}H_{10}N_2NaO_6S_2$ 388.9872 $[M + Na]^+$, found 388.9882.

1.5. General synthetic procedures for 12 and 13

To a solution of **11** (0.5 mmol) in THF was added sodium hydroxide (0.2 mL, 25% w/w) at 0 °C. The mixture was stirred at 0 °C for 10 min. Then the corresponding alcohol (2 mmol) was dissolved in the mixture and stirred at room temperature for 4 h. The reaction was monitored by TLC. Butylene oxide was evaporated under vacuum. The residue was diluted with H_2O (20 mL) and was extracted three times with DCM (20 mL). The combined organic layer was sequentially washed with water and brine, dried with anhydrous Na_2SO_4, and concentrated in vacuo. The crude was purified *via* column chromatography (DCM/MeOH 400:1, v/v) to give the product as a white crystal.

1.5.1. 4-(2-Hydroxyethoxy)-3-(phenylsulfonyl)-1,2,5-oxadiazole 2-oxide (12)

White solid, yield 48%, m.p. 128-130 °C. ^{1}H NMR (400 MHz, $CDCl_3$) δ 8.07 (d, J = 8.2 Hz, 2H), 7.77 (t, J = 7.5 Hz, 1H), 7.63 (t, J = 7.8 Hz, 2H), 4.59 – 4.49 (m, 2H), 4.08 – 4.02 (m, 2H). ^{13}C NMR (100 MHz, $CDCl_3$) δ 159.1, 137.8, 135.9, 129.9, 128.7, 110.7, 73.0, 60.5. HRMS (ESI) calcd for $C_{10}H_{10}N_2NaO_6S$ 309.0152 $[M + Na]^+$, found 309.0142.

1.5.2. 4-((4-Hydroxybut-2-yn-1-yl)oxy)-3-(phenylsulfonyl)-1,2,5-oxadiazole 2-oxide (13)

White solid, yield 53%, m.p. 116-118 °C. ^{1}H NMR (400 MHz, $CDCl_3$) δ 8.07 (d, J = 7.7 Hz, 2H), 7.76 (t, J = 7.4 Hz, 1H), 7.63 (t, J = 7.7 Hz, 2H), 5.10 (s, 2H), 4.34 (s, 2H). ^{13}C NMR (100 MHz, $CDCl_3$) δ 158.2, 138.0, 135.9, 129.8, 128.8, 110.8, 88.3, 77.7, 59.0, 51.1. HRMS (ESI) calcd for $C_{12}H_{10}N_2NaO_6S$ 333.0512 $[M + Na]^+$, found 333.0512.

1.6. General synthetic procedures for 14a-b

A mixture of **12** (0.63 mmol), the corresponding acid anhydride (0.76 mmol), and DMAP (0.32 mmol) in anhydrous DCM (5 mL) was stirred at room temperature for 6 h. The reaction was monitored by TLC. The mixture was diluted with H_2O (10 mL) and extracted three times with DCM (20 mL). The combined organic layer was sequentially washed with water and brine, dried with anhydrous Na_2SO_4, and concentrated in vacuo. The crude was purified *via* column chromatography (DCM/MeOH 150:1, v/v) to give the product as a white solid.

1.6.1. 4-(2-((3-Carboxypropanoyl)oxy)ethoxy)-3-(phenylsulfonyl)-1,2,5-oxadiazole 2-oxide (14a)

White solid, yield 67%, m.p. 118-120 °C. ^{1}H NMR (400 MHz, $CDCl_3$) δ 8.07 (d, J = 7.3 Hz, 2H), 7.76 (t, J = 7.5 Hz, 1H), 7.63 (t, J = 7.9 Hz, 2H), 4.72 – 4.59 (m, 2H), 4.57 – 4.44 (m, 2H), 2.92 – 2.51 (m, 4H). ^{13}C NMR (100 MHz, $CDCl_3$) δ 176.9, 171.9, 158.8, 138.2, 135.8, 129.8, 128.8, 110.6, 69.0, 61.6, 29.0, 28.9. HRMS (ESI) calcd for $C_{14}H_{14}N_2NaO_9S$ 409.0312 $[M + Na]^+$, found 409.0295.

1.6.2. 4-(2-((4-carboxybutanoyl)oxy)ethoxy)-3-(phenylsulfonyl)-1,2,5-oxadiazole 2-oxide (14b)

White solid, yield 53%, m.p. 91-93 °C. ^{1}H NMR (400 MHz, $CDCl_3$) δ 8.06 (d, J = 8.1 Hz, 2H), 7.76 (t, J = 7.5 Hz, 1H), 7.62 (t, J = 7.8 Hz, 2H), 4.66 – 4.60 (m, 2H), 4.55 – 4.48 (m, 2H), 2.47 (q, J = 7.0 Hz, 4H), 1.98 (p, J = 7.2 Hz, 2H). ^{13}C NMR (100 MHz, $CDCl_3$) δ 178.6, 172.7, 158.8, 138.0, 135.8, 129.8, 128.8, 110.6, 69.0, 61.3, 33. 0, 32.9, 19.7. HRMS (ESI) calcd for $C_{15}H_{16}N_2NaO_9S$ 423.0469 $[M + Na]^+$, found 423.0465.

1.7. General synthetic procedures for 15a-b

A mixture of **13** (0.6 mmol), the corresponding acid anhydride (0.6 mmol), and DMAP (0.3 mmol) in absolute DCM (5 mL) was stirred at room temperature for 8 h. The reaction was monitored by TLC. The mixture was diluted with H_2O (10 mL) and extracted three times with DCM (20 mL). The combined organic layer was sequentially washed with water and brine, dried with anhydrous Na_2SO_4, and concentrated in vacuo. The crude was purified *via* column chromatography (DCM/MeOH 150:1, v/v) to give the product as a white crystal.

1.7.1. 4-((4-((3-carboxypropanoyl)oxy)but-2-yn-1-yl)oxy)-3-(phenylsulfonyl)-1,2,5-oxadiaz (15a)

White solid, yield 54%, m.p. 106-108 °C. ^{1}H NMR (400 MHz, $CDCl_3$) δ 8.07 (d, J = 7.4 Hz, 2H), 7.77 (t, J = 7.5 Hz, 1H), 7.63 (t, J = 7.9 Hz, 2H), 5.10 (s, 2H), 4.77 (s, 2H), 2.70 (dt, J = 8.1, 4.7 Hz, 4H). ^{13}C NMR (100 MHz, $CDCl_3$) δ 177.2, 171.4, 158.1, 138.1, 135.9, 129.9, 128.8, 110.8, 83.9, 78.8, 58.8, 52.3, 28.8, 28.8. HRMS (ESI) calcd for $C_{16}H_{14}N_2NaO_9S$ 433.0312 $[M + Na]^+$, found 433.0317.

1.7.2. 4-(4-((4-Carboxybutanoyl)oxy)but-2-yn-1-yl)oxy)-3-(phenylsulfonyl)-1,2,5-oxadiazole 2-oxide (15b)

White solid, yield 54%, m.p. 94-96 °C. ^{1}H NMR (400 MHz, $CDCl_3$) δ 8.07 (d, J = 7.6 Hz, 2H), 7.77 (t, J = 7.5 Hz, 1H), 7.63 (t, J = 7.8 Hz, 2H), 5.10 (s, 2H), 4.74 (s, 2H), 2.46 (td, J = 7.3, 4.9 Hz, 4H), 1.97 (p, J = 7.3 Hz, 2H). ^{13}C NMR (100 MHz, $CDCl_3$) δ 179.0, 172.1, 158.0, 137.7, 135.8, 129.8, 128.7, 110.6, 83.9, 78.6, 58.6, 52.0, 32.8, 19.6. HRMS (ESI) calcd for $C_{16}H_{14}N_2NaO_9S$ 447.0469 $[M + Na]^+$, found 447.0486.

1.8. General synthetic procedures for Ia-d, IIa-b, IIIa-d, IVa-d, Va-b, and VIa-b

A solution of intermediates **5-10** (0.08 mmol), NO donor intermediates **12a-b** and **13a-b** (0.10 mmol), EDCI (0.12 mmol), and DMAP (0.01 mmol) in anhydrous DCM (2 mL) was stirred at room temperature for 8 h. The reaction was monitored by TLC. The reaction mixture was diluted with DCM (5 mL). The organic layer was sequentially washed with water and brine, dried with anhydrous Na_2SO_4, and concentrated in vacuo. The crude was purified *via* column

chromatography (DCM/MeOH) to give the product as a light yellow liquid.

1.8.1. 4-(2-((4-(2-((2-((1R,3R,4S)-3-(3-chloroprop-1-en-2-yl)-4-methyl-4-vinylcyclohexyl)allyl)(methyl)amino)ethoxy)-4-oxobutanoyl)oxy)ethoxy)-3-(phenylsulfonyl)-1,2,5-oxadiazole 2-oxide (Ia)

Pale yellow liquid, yield 69%. ^{1}H NMR (400 MHz, $CDCl_3$) δ 8.07 (d, J = 7.8 Hz, 2H), 7.75 (d, J = 7.5 Hz, 1H), 7.63 (t, J = 7.8 Hz, 2H), 5.78 (dd, J = 17.8, 10.4 Hz, 1H), 5.26 (s, 1H), 5.02 – 4.87 (m, 5H), 4.65 – 4.59 (m, 2H), 4.57 – 4.48 (m, 2H), 4.21 (t, J = 5.8 Hz, 2H), 4.09 (d, J = 11.7 Hz, 1H), 3.96 (d, J = 11.8 Hz, 1H), 3.01 (s, 2H), 2.63 (d, J = 5.5 Hz, 6H), 2.32 – 2.22 (m, 4H), 1.66 – 1.42 (m, 6H), 0.97 (s, 3H). ^{13}C NMR (100 MHz, $CDCl_3$) δ 172.0, 172.0, 158.7, 149.2, 147.7, 138.1, 135.6, 129.7, 128.6, 116.1, 111.2, 110.4, 77.0, 68.9, 62.5, 61.4, 55.3, 50.8, 47.9, 42.6, 41.5, 39.8, 33.8, 29.0, 28.9, 27.1, 15.8. HRMS (ESI) calcd for $C_{32}H_{43}ClN_3O_9S$ 680.2403 $[M + H]^+$, found 680.2441.

1.8.2. 4-(2-((5-(2-((2-((1R,3R,4S)-3-(3-chloroprop-1-en-2-yl)-4-methyl-4-vinylcyclohexyl)allyl)(methyl)amino)ethoxy)-5-oxopentanoyl)oxy)ethoxy)-3-(phenylsulfonyl)-1,2,5-oxadiazole 2-oxide (Ib)

Pale yellow liquid, yield 71%. ^{1}H NMR (400 MHz, $CDCl_3$) δ 8.06 (d, J = 8.5 Hz, 2H), 7.76 (t, J = 7.5 Hz, 1H), 7.63 (t, J = 7.9 Hz, 2H), 5.87 – 5.71 (m, 1H), 5.26 (s, 1H), 5.01 – 4.86 (m, 5H), 4.67 – 4.60 (m, 2H), 4.53 – 4.46 (m, 2H), 4.17 (t, J = 5.7 Hz, 2H), 4.09 (d, J = 11.6 Hz, 1H), 3.96 (d, J = 11.7 Hz, 1H), 2.95 (s, 2H), 2.57 (t, J = 5.1 Hz, 2H), 2.43 (dt, J = 17.0, 7.3 Hz, 4H), 2.28 (dd, J = 11.9, 4.0 Hz, 1H), 2.16 – 2.07 (m, 1H), 2.02 – 1.93 (m, 2H), 1.65 – 1.35 (m, 6H), 0.96 (s, 3H). ^{13}C NMR (100 MHz, $CDCl_3$) δ 173.0, 172.8, 158.8, 151.4, 149.4, 147.8, 138.1, 135.8, 129.8, 128.7, 116.3, 110.9, 110.5, 69.0, 63.3, 62.5, 61.2, 55.4, 51.1, 47.7, 42.9, 41.5, 39.9, 39.9, 33.9, 33.2, 33.1, 27.1, 20.0, 15.8. HRMS (ESI) calcd for $C_{33}H_{45}ClN_3O_9S$ 694.2560 $[M + H]^+$, found 694.2562.

1.8.3. 4-((4-((4-(2-((2-((1R,3R,4S)-3-(3-chloroprop-1-en-2-yl)-4-methyl-4-vinylcyclohexyl)allyl)(methyl)amino)ethoxy)-4-oxobutanoyl)oxy)but-2-yn-1-yl)oxy)-3-(phenylsulfonyl)-1,2,5-oxadiazole 2-oxide (Ic)

Pale yellow liquid, yield 74%. ^{1}H NMR (400 MHz, $CDCl_3$) δ 8.07 (d, J = 7.4 Hz, 2H), 7.77 (t, J = 7.5 Hz, 1H), 7.64 (t, J = 7.9 Hz, 2H), 5.78 (dd, J = 16.9, 11.3 Hz, 1H), 5.26 (s, 1H), 5.10 (s, 2H), 5.05 – 4.85 (m, 5H), 4.76 (s, 2H), 4.20 (t, J = 5.6 Hz, 2H), 4.12 – 4.06 (m, 1H), 3.96 (d, J = 11.7 Hz, 1H), 2.97 (s, 2H), 2.70 – 2.56 (m, 6H), 2.31 – 2.09 (m, 5H), 1.70 – 1.41 (m, 6H), 0.97 (s, 3H). ^{13}C NMR (100 MHz, $CDCl_3$) δ 172.2, 171.6, 158.0, 151.3, 149.4, 149.4, 147.7, 137.9, 135.9, 129.9, 128.8, 116.3, 111.3, 110.7, 83.9, 78.7, 63.2, 62.8, 58.7, 55.3, 52.3, 51.1, 47.7, 42.8, 41.4, 39.9, 39.9, 33.9, 29.0, 28.9, 27.1, 15.8. HRMS (ESI) calcd for $C_{34}H_{43}ClN_3O_9S$ 704.2403 [M + H]$^+$, found 704.2408.

1.8.4. 4-((4-((5-(2-((2-((1R,3R,4S)-3-(3-chloroprop-1-en-2-yl)-4-methyl-4-vinylcyclohexyl)allyl)(methyl)amino)ethoxy)-5-oxopentanoyl)oxy)but-2-yn-1-yl)oxy)-3-(phenylsulfonyl)-1,2,5-oxadiazole 2-oxide (Id)

Pale yellow liquid, yield 73%. ^{1}H NMR (400 MHz, $CDCl_3$) δ 8.07 (d, J = 7.4 Hz, 2H), 7.77 (t, J = 7.5 Hz, 1H), 7.63 (t, J = 7.8 Hz, 2H), 5.77 (dd, J = 17.1, 11.2 Hz, 1H), 5.25 (s, 1H), 5.09 (s, 2H), 4.98 – 4.83 (m, 5H), 4.73 (s, 2H), 4.17 (t, J = 5.8 Hz, 2H), 4.09 (d, J = 11.7 Hz, 1H), 3.96 (d, J = 11.7 Hz, 1H), 2.96 (s, 2H), 2.57 (t, J = 5.5 Hz, 2H), 2.41 (dt, J = 17.1, 7.3 Hz, 4H), 2.31 – 2.18 (m, 4H), 2.12 (s, 1H), 1.96 (p, J = 7.1 Hz, 2H), 1.68 – 1.41 (m, 6H), 0.96 (s, 3H). ^{13}C NMR (100 MHz, $CDCl_3$) δ 172.9, 172.2, 158.0, 151.3, 149.3, 147.7, 137.8, 135.9, 129.8, 128.8, 116.3, 111.3, 110.9, 110.7, 84.0, 78.6, 63.2, 62.4, 58.7, 55.4, 52.0, 51.1, 47.7, 39.9, 33.8, 33.2, 33.0, 27.1, 20.0, 15.8. HRMS (ESI) calcd for $C_{35}H_{45}ClN_3O_9S$ 718.2560 [M + H]$^+$, found 718.2553.

1.8.5. 4-(2-((4-(2-((2-((1R,3R,4S)-3-(3-chloroprop-1-en-2-yl)-4-methyl-4-vinylcyclohexyl)allyl)(isopropyl)amino)ethoxy)-4-oxobutanoyl)oxy)ethoxy)-3-(phenylsulfonyl)-1,2,5-oxadiazole 2-oxide (IIa)

Pale yellow liquid, yield 58%. ^{1}H NMR (400 MHz, $CDCl_3$) δ 8.07 (d, J = 7.5 Hz, 2H), 7.76 (t, J = 7.5 Hz, 1H), 7.63 (t, J = 7.8 Hz, 2H), 5.77 (dd, J = 17.6, 10.6 Hz, 1H), 5.25 (s, 1H), 5.03 – 4.84 (m, 5H), 4.66 – 4.58 (m, 2H), 4.56 – 4.47 (m, 2H), 4.11 – 3.93 (m, 4H), 3.06 – 2.82 (m, 3H), 2.72 – 2.56 (m, 6H), 2.30 – 2.09 (m, 2H), 1.65 – 1.40 (m, 6H), 1.02 – 0.90 (m, 9H). ^{13}C NMR (100 MHz, $CDCl_3$) δ 172.2, 172.1, 158.7, 149.8, 149.3, 147.7, 138.0, 135.7, 129.7, 128.7, 116.2, 111.2, 110.4, 110.0, 68.9, 64.0, 61.4, 55.6, 51.2, 50.2, 47.7, 41.3, 39.9, 39.9, 34.0, 28.9, 28.9, 27.1,

18.0, 17.7, 15.7. HRMS (ESI) calcd for $C_{34}H_{47}ClN_3O_9S$ 708.2716 $[M + H]^+$, found 708.2703.

1.8.6. 4-(2-((5-(2-((2-((1R,3R,4S)-3-(3-chloroprop-1-en-2-yl)-4-methyl-4-vinylcyclohexyl)allyl)(isopropyl)amino)ethoxy)-5-oxopentanoyl)oxy)ethoxy)-3-(phenylsulfonyl)-1,2,5-oxadiazole 2-oxide (IIb)

Pale yellow liquid, yield 68%. ^{1}H NMR (400 MHz, $CDCl_3$) δ 8.06 (d, J = 7.4 Hz, 2H), 7.76 (t, J = 7.5 Hz, 1H), 7.62 (t, J = 7.8 Hz, 2H), 5.77 (dd, J = 16.9, 11.3 Hz, 1H), 5.25 (s, 1H), 5.03 – 4.82 (m, 5H), 4.65 – 4.56 (m, 2H), 4.54 – 4.43 (m, 2H), 4.12 – 3.91 (m, 4H), 3.08 – 2.79 (m, 3H), 2.58 (t, J = 6.6 Hz, 2H), 2.42 (dt, J = 26.0, 7.3 Hz, 4H), 2.26 (dd, J = 12.7, 3.4 Hz, 1H), 2.13 (t, J = 11.3 Hz, 1H), 1.97 (p, J = 7.3 Hz, 2H), 1.66 – 1.41 (m, 6H), 1.01 – 0.87 (m, 9H). ^{13}C NMR (100 MHz, $CDCl_3$) δ 172.9, 172.8, 158.8, 149.8, 149.3, 147.8, 138.0, 135.8, 129.8, 128.7, 116.3, 111.3, 110.5, 110.1, 69.0, 63.8, 61.2, 55.6, 51.2, 50.2, 47.8, 41.4, 40.0, 39.9, 34.1, 33.2, 33.1, 27.1, 20.0, 18.1, 17.8, 15.8. HRMS (ESI) calcd for $C_{35}H_{48}ClN_3O_9S$ 722.2873 $[M + H]^+$, found 722.2873.

1.8.7. 4-((4-((4-(2-((2-((1R,3R,4S)-3-(3-chloroprop-1-en-2-yl)-4-methyl-4-vinylcyclohexyl)allyl)(isopropyl)amino)ethoxy)-4-oxobutanoyl)oxy)but-2-yn-1-yl)oxy)-3-(phenylsulfonyl)-1,2,5-oxadiazole 2-oxide (IIc)

Pale yellow liquid, yield 47%. ^{1}H NMR (400 MHz, $CDCl_3$) δ 8.08 (d, J = 7.7 Hz, 2H), 7.77 (t, J = 7.4 Hz, 1H), 7.64 (t, J = 7.8 Hz, 2H), 5.78 (dd, J = 17.1, 11.1 Hz, 1H), 5.25 (s, 1H), 5.10 (s, 2H), 4.76 (s, 2H), 4.18 – 3.91 (m, 4H), 2.98 (d, J = 45.6 Hz, 3H), 2.65 (dt, J = 10.6, 5.1 Hz, 6H), 2.31 – 2.11 (m, 2H), 1.64 – 1.41 (m, 6H), 0.97 (s, 9H). ^{13}C NMR (100 MHz, $CDCl_3$) δ 172.1, 171.6, 158.1, 149.4, 147.9, 138.1, 135.8, 129.9, 128.8, 116.2, 111.3, 110.7, 84.0, 78.8, 58.7, 55.7, 52.2, 51.1, 48.0, 47.9, 41.5, 40.0, 34.2, 29.1, 29.0, 27.3, 22.8, 18.1, 17.8, 15.9. HRMS (ESI) calcd for $C_{36}H_{47}ClN_3O_9S$ 732.2716 $[M + H]^+$, found 732.2711.

1.8.8. 4-((4-((5-(2-((2-((1R,3R,4S)-3-(3-chloroprop-1-en-2-yl)-4-methyl-4-vinylcyclohexyl)allyl)(isopropyl)amino)ethoxy)-5-oxopentanoyl)oxy)but-2-yn-1-yl)oxy)-3-(phenylsulfonyl)-1,2,5-oxadiazole 2-oxide (IId)

Pale yellow liquid, yield 53%. ^{1}H NMR (400 MHz, $CDCl_3$) δ 8.07 (d, J = 7.4 Hz, 2H), 7.77 (t, J = 7.5 Hz, 1H), 7.63 (t, J = 7.8 Hz, 2H), 5.78 (dd, J = 17.0, 11.2 Hz, 1H), 5.25 (s, 1H), 5.09 (s,

2H), 5.04 – 4.81 (m, 5H), 4.74 (s, 2H), 4.13 – 3.91 (m, 4H), 3.09 – 2.79 (m, 3H), 2.59 (t, *J* = 6.2 Hz, 2H), 2.40 (dt, *J* = 26.3, 7.4 Hz, 5H), 2.26 (dd, *J* = 12.2, 3.5 Hz, 1H), 2.14 (s, 1H), 1.96 (p, *J* = 7.3 Hz, 2H), 1.66 – 1.41 (m, 6H), 1.06 – 0.89 (m, 9H). ^{13}C NMR (100 MHz, $CDCl_3$) δ 172.7, 172.0, 157.9, 149.3, 147.7, 137.9, 135.7, 129.7, 128.7, 116.1, 111.1, 110.6, 110.0, 84.0, 78.5, 63.7, 58.6, 55.6, 51.8, 51.0, 50.3, 47.9, 47.9, 41.4, 39.9, 39.9, 34.1, 33.2, 33.0, 27.1, 20.0, 18.0, 17.7, 15.8. HRMS (ESI) calcd for $C_{37}H_{49}ClN_3O_9S$ 746.2873 [M + H]$^+$, found 746.2871.

1.8.9. 4-(2-((4-(((S)-1-(2-((1R,3R,4S)-3-(3-chloroprop-1-en-2-yl)-4-methyl-4-vinylcyclohexyl)allyl)pyrrolidin-3-yl)oxy)-4-oxobutanoyl)oxy)ethoxy)-3-(phenylsulfonyl)-1,2,5-oxadiazole 2-oxide (IIIa)

Pale yellow liquid, yield 68%. ^1H NMR (400 MHz, $CDCl_3$) δ 8.06 (d, *J* = 7.5 Hz, 2H), 7.76 (t, *J* = 7.5 Hz, 1H), 7.63 (t, *J* = 7.8 Hz, 2H), 5.77 (dd, *J* = 17.1, 11.2 Hz, 1H), 5.26 (s, 1H), 5.17 (s, 1H), 5.00 – 4.83 (m, 5H), 4.66 – 4.57 (m, 2H), 4.56 – 4.48 (m, 2H), 4.09 (d, *J* = 11.6 Hz, 1H), 3.96 (d, *J* = 11.7 Hz, 1H), 3.12 (d, *J* = 13.4 Hz, 1H), 2.99 (d, *J* = 13.4 Hz, 1H), 2.76 – 2.60 (m, 7H), 2.40 (s, 1H), 2.31 – 2.19 (m, 2H), 1.87 – 1.79 (m, 1H), 1.67 – 1.40 (m, 6H), 0.96 (s, 3H). ^{13}C NMR (100 MHz, $CDCl_3$) δ 172.2, 172.2, 158.8, 149.3, 147.7, 138.0, 135.8, 129.8, 128.7, 116.2, 111.3, 110.5, 74.7, 68.9, 61.5, 60.8, 60.0, 53.0, 51.1, 47.6, 42.0, 39.9, 39.8, 34.0, 31.9, 29.2, 29.0, 27.1, 15.8. HRMS (ESI) calcd for $C_{33}H_{43}ClN_3O_9S$ 692.2403 [M + H]$^+$, found 692.2394.

1.8.10. 4-(2-((5-(((S)-1-(2-((1R,3R,4S)-3-(3-chloroprop-1-en-2-yl)-4-methyl-4-vinylcyclohexyl)allyl)pyrrolidin-3-yl)oxy)-5-oxopentanoyl)oxy)ethoxy)-3-(phenylsulfonyl)-1,2,5-oxadiazole 2-oxide (IIIb)

Pale yellow liquid, yield 62%. ^1H NMR (400 MHz, $CDCl_3$) δ 8.05 (d, *J* = 7.5 Hz, 2H), 7.76 (t, *J* = 7.5 Hz, 1H), 7.63 (t, *J* = 7.8 Hz, 2H), 5.77 (dd, *J* = 17.0, 11.2 Hz, 1H), 5.25 (s, 1H), 5.16 (s, 1H), 5.03 – 4.85 (m, 5H), 4.66 – 4.59 (m, 2H), 4.53 – 4.46 (m, 2H), 4.09 (d, *J* = 11.6 Hz, 1H), 3.96 (d, *J* = 11.7 Hz, 1H), 3.17 – 2.91 (m, 2H), 2.71 (d, *J* = 6.6 Hz, 2H), 2.59 (d, *J* = 9.8 Hz, 1H), 2.42 (dt, *J* = 23.1, 7.3 Hz, 5H), 2.31 – 2.19 (m, 2H), 2.11 (s, 1H), 2.00 – 1.92 (m, 2H), 1.85 – 1.76 (m, 1H), 1.67 – 1.40 (m, 6H), 0.96 (s, 3H). ^{13}C NMR (100 MHz, $CDCl_3$) δ 172.9, 172.8, 158.8, 149.3, 147.7, 138.0, 135.8, 129.8, 128.7, 116.2, 111.3, 110.5, 74.4, 69.0, 61.2, 60.9, 60.0, 53.0, 51.1, 47.6, 42.0, 39.9, 39.8, 34.0, 33.4, 33.1, 32.0, 27.1, 20.0, 15.8. HRMS (ESI) calcd for

$C_{34}H_{45}ClN_3O_9S$ 706.2560 [M + H]$^+$, found 706.2555.

1.8.11. 4-((4-((4-(((S)-1-(2-((1R,3R,4S)-3-(3-chloroprop-1-en-2-yl)-4-methyl-4-vinylcyclohexyl)allyl)pyrrolidin-3-yl)oxy)-4-oxobutanoyl)oxy)but-2-yn-1-yl)oxy)-3-(phenylsulfonyl)-1,2,5-oxadiazole 2-oxide (IIIc)

Pale yellow liquid, yield 53%. ^{1}H NMR (400 MHz, $CDCl_3$) δ 8.07 (d, J = 7.4 Hz, 2H), 7.77 (t, J = 7.5 8Hz, 1H), 7.63 (t, J = 7.9 Hz, 2H), 5.78 (dd, J = 17.9, 10.3 Hz, 1H), 5.26 (s, 1H), 5.19 (s, 1H), 5.09 (s, 2H), 5.02 – 4.84 (m, 5H), 4.76 (s, 2H), 4.09 (d, J = 11.4 Hz, 1H), 3.97 (d, J = 11.8 Hz, 1H), 3.16 – 2.95 (m, 2H), 2.76 – 2.60 (m, 7H), 2.41 (s, 1H), 2.31 – 2.08 (m, 3H), 1.88 – 1.79 (m, 1H), 1.68 – 1.43 (m, 6H), 0.97 (s, 3H). ^{13}C NMR (100 MHz, $CDCl_3$) δ 172.0, 171.6, 158.1, 149.4, 147.8, 147.8, 138.0, 135.8, 129.8, 128.8, 116.2, 111.3, 110.7, 84.0, 78.8, 74.8, 60.9, 60.0, 58.7, 53.0, 52.2, 51.0, 47.9, 42.2, 39.9, 39.9, 34.1, 32.0, 29.2, 29.0, 27.2, 15.9. HRMS (ESI) calcd for $C_{35}H_{43}ClN_3O_9S$ 716.2403 [M + H]$^+$, found 716.2400.

1.8.12. 4-((4-((5-(((S)-1-(2-((1R,3R,4S)-3-(3-chloroprop-1-en-2-yl)-4-methyl-4-vinylcyclohexyl)allyl)pyrrolidin-3-yl)oxy)-5-oxopentanoyl)oxy)but-2-yn-1-yl)oxy) -3-(phenylsulfonyl)-1,2,5-oxadiazole 2-oxide (IIId)

Pale yellow liquid, yield 63%. ^{1}H NMR (400 MHz, $CDCl_3$) δ 8.07 (d, J = 7.4 Hz, 2H), 7.76 (t, J = 7.5 Hz, 1H), 7.63 (t, J = 7.8 Hz, 2H), 5.78 (dd, J = 17.9, 10.3 Hz, 1H), 5.26 (s, 1H), 5.17 (td, J = 6.2, 3.2 Hz, 1H), 5.09 (s, 2H), 5.01 – 4.82 (m, 5H), 4.74 (s, 2H), 4.09 (d, J = 11.5 Hz, 1H), 3.96 (d, J = 11.8 Hz, 1H), 3.11 (d, J = 13.4 Hz, 1H), 2.99 (d, J = 13.4 Hz, 1H), 2.78 – 2.66 (m, 2H), 2.60 (d, J = 10.9 Hz, 1H), 2.40 (dt, J = 23.9, 7.3 Hz, 5H), 2.31 – 2.17 (m, 2H), 2.10 (dq, J = 7.6, 5.3, 3.5 Hz, 1H), 1.95 (p, J = 7.3 Hz, 2H), 1.81 (dq, J = 13.7, 8.7, 6.9 Hz, 1H), 1.70 – 1.39 (m, 6H), 0.97 (s, 3H). ^{13}C NMR (100 MHz, $CDCl_3$) δ 172.8, 172.2, 158.0, 151.1, 149.3, 147.7, 137.8, 135.9, 129.8, 128.7, 116.2, 111.3, 110.7, 110.4, 84.0, 78.6, 74.4, 60.8, 60.0, 58.7, 53.0, 52.0, 51.0, 47.6, 42.0, 39.9, 39.8, 34.0, 33.3, 33.0, 31.9, 27.1, 20.0, 15.8. HRMS (ESI) calcd for $C_{36}H_{45}ClN_3O_9S$ 730.2560 [M + H]$^+$, found 730.255.

1.8.13. 4-(2-((4-((1-(2-((1R,3R,4S)-3-(3-chloroprop-1-en-2-yl)-4-methyl-4-vinylcyclohexyl)allyl)piperidin-4-yl)methoxy)-4-oxobutanoyl)oxy)ethoxy)-3-(phe

nylsulfonyl)-1,2,5-oxadiazole 2-oxide (IVa)

Pale yellow liquid, yield 67%. ^{1}H NMR (400 MHz, $CDCl_3$) δ 8.06 (d, J = 7.7 Hz, 2H), 7.76 (t, J = 7.5 Hz, 1H), 7.63 (t, J = 7.8 Hz, 2H), 5.78 (dd, J = 17.1, 11.1 Hz, 1H), 5.25 (s, 1H), 4.98 – 4.84 (m, 5H), 4.68 – 4.58 (m, 2H), 4.57 – 4.46 (m, 2H), 4.12 – 3.88 (m, 4H), 2.88 (d, J = 17.1 Hz, 4H), 2.68 (s, 4H), 2.33 – 1.95 (m, 3H), 1.85 (s, 2H), 1.73 – 1.39 (m, 10H), 0.96 (s, 3H). ^{13}C NMR (100 MHz, $CDCl_3$) δ 172.2, 172.2, 158.7, 149.3, 147.7, 137.9, 135.7, 129.7, 128.7, 116.1, 111.2, 110.4, 69.3, 68.9, 63.6, 61.4, 53.4, 53.3, 51.1, 47.6, 42.1, 39.9, 39.7, 35.3, 33.9, 28.9, 28.9, 27.0, 15.7. HRMS (ESI) calcd for C35H47ClN3O9S 720.2716 [M + H]$^+$, found 720.2709.

1.8.14. 4-(2-((5-((1-(2-((1R,3R,4S)-3-(3-chloroprop-1-en-2-yl)-4-methyl-4-vinylcyclohexyl)allyl)piperidin-4-yl)methoxy)-5-oxopentanoyl)oxy)ethoxy)-3-(phenylsulfonyl)-1,2,5-oxadiazole 2-oxide (IVb)

Pale yellow liquid, yield 70%. ^{1}H NMR (400 MHz, $CDCl_3$) δ 8.06 (d, J = 7.4 Hz, 2H), 7.76 (t, J = 7.5 Hz, 1H), 7.62 (t, J = 7.9 Hz, 2H), 5.78 (dd, J = 18.0, 10.3 Hz, 1H), 5.26 (s, 1H), 5.01 – 4.85 (m, 5H), 4.72 – 4.59 (m, 2H), 4.54 – 4.46 (m, 2H), 4.08 (d, J = 11.7 Hz, 1H), 3.98 – 3.90 (m, 3H), 3.03 – 2.80 (m, 4H), 2.42 (dt, J = 18.4, 7.3 Hz, 4H), 2.31 – 2.23 (m, 1H), 2.15 – 2.07 (m, 1H), 2.03 – 1.86 (m, 4H), 1.68 – 1.42 (m, 10H), 0.97 (s, 3H). ^{13}C NMR (100 MHz, $CDCl_3$) δ 173.0, 172.8, 158.8, 149.4, 147.8, 138.1, 135.8, 129.8, 128.7, 116.2, 111.3, 110.5, 69.0, 69.0, 63.6, 61.2, 53.4, 53.3, 51.1, 47.8, 42.2, 39.9, 39.8, 35.4, 34.0, 33.3, 33.2, 29.8, 28.8, 27.1, 20.1, 15.8. HRMS (ESI) calcd for $C_{36}H_{49}ClN_3O_9S$ 734.2873 [M + H]$^+$, found 734.889.

1.8.15. 4-((4-((4-((1-(2-((1R,3R,4S)-3-(3-chloroprop-1-en-2-yl)-4-methyl-4-vinylcyclohexyl)allyl)piperidin-4-yl)methoxy)-4-oxobutanoyl)oxy)but-2-yn-1-yl)oxy)-3-(phenylsulfonyl)-1,2,5-oxadiazole 2-oxide (IVc)

Pale yellow liquid, yield 65%. ^{1}H NMR (400 MHz, $CDCl_3$) δ 8.07 (d, J = 7.4 Hz, 2H), 7.76 (t, J = 7.5 Hz, 1H), 7.63 (t, J = 7.8 Hz, 2H), 5.78 (dd, J = 17.9, 10.4 Hz, 1H), 5.26 (s, 1H), 5.09 (s, 2H), 5.02 – 4.85 (m, 5H), 4.76 (s, 2H), 4.08 (d, J = 11.4 Hz, 1H), 3.99 – 3.94 (m, 3H), 2.98 (s, 4H), 2.66 (q, J = 3.7 Hz, 4H), 2.30 – 2.25 (m, 1H), 2.17 – 2.11 (m, 1H), 1.97 (d, J = 17.5 Hz, 2H), 1.71 – 1.43 (m, 10H), 0.97 (s, 3H). ^{13}C NMR (100 MHz, $CDCl_3$) δ 172.1, 171.6, 158.1, 149.3, 147.9, 138.1, 135.8, 129.8, 128.8, 116.1, 111.3, 110.7, 84.0, 78.8, 69.1, 65.7, 63.5, 58.7, 53.4, 53.3, 52.2,

50.9, 48.0, 42.3, 39.9, 39.9, 35.3, 34.1, 29.8, 29.1, 29.0, 27.2, 15.9. HRMS (ESI) calcd for $C_{37}H_{47}ClN_3O_9S$ 744.2716 $[M + H]^+$, found 744.2700.

1.8.16. 4-((4-((5-((1-(2-((1R,3R,4S)-3-(3-chloroprop-1-en-2-yl)-4-methyl-4-vinylcyclohexyl)allyl)piperidin-4-yl)methoxy)-5-oxopentanoyl)oxy)but-2-yn-1-yl) oxy)-3-(phenylsulfonyl)-1,2,5-oxadiazole 2-oxide (IVd)

Pale yellow liquid, yield 60%. ^1H NMR (400 MHz, $CDCl_3$) δ 8.07 (d, J = 7.5 Hz, 2H), 7.77 (t, J = 7.5 Hz, 1H), 7.63 (t, J = 7.8 Hz, 2H), 5.78 (dd, J = 17.5, 10.8 Hz, 1H), 5.25 (s, 1H), 5.09 (s, 2H), 5.00 – 4.85 (m, 5H), 4.74 (s, 2H), 4.09 (d, J = 11.6 Hz, 1H), 4.01 – 3.87 (m, 3H), 2.89 (d, J = 17.0 Hz, 4H), 2.41 (dt, J = 18.4, 7.3 Hz, 5H), 2.30 – 2.23 (m, 1H), 2.16 – 2.06 (m, 1H), 2.04 – 1.77 (m, 5H), 1.73 – 1.39 (m, 10H), 0.96 (s, 3H). ^{13}C NMR (100 MHz, $CDCl_3$) δ 173.0, 172.2, 158.0, 149.4, 147.8, 137.8, 135.9, 129.8, 128.8, 116.2, 111.3, 110.7, 84.0, 78.6, 76.8, 69.1, 63.7, 58.7, 53.5, 53.4, 52.0, 51.1, 47.7, 42.2, 39.9, 39.8, 35.4, 34.0, 33.2, 33.0, 29.0, 27.0, 20.1, 15.8. HRMS (ESI) calcd for $C_{38}H_{49}ClN_3O_9S$ 758.2873 $[M + H]^+$, found 758.2866.

1.8.17. 4-(2-((4-((1-(2-((1R,3R,4S)-3-(3-chloroprop-1-en-2-yl)-4-methyl-4-vinylcyclohexyl)allyl)piperidin-3-yl)methoxy)-4-oxobutanoyl)oxy)ethoxy)-3-(phenylsulfonyl)-1,2,5-oxadiazole 2-oxide (Va)

Pale yellow liquid, yield 70%. ^1H NMR (400 MHz, $CDCl_3$) δ 8.07 (d, J = 7.4 Hz, 2H), 7.77 (t, J = 7.5 Hz, 1H), 7.63 (t, J = 7.9 Hz, 2H), 5.78 (dd, J = 17.9, 10.3 Hz, 1H), 5.26 (s, 1H), 4.98 – 4.82 (m, 5H), 4.65 – 4.60 (m, 2H), 4.54 – 4.50 (m, 2H), 4.14 – 3.87 (m, 4H), 2.88 (s, 2H), 2.67 (t, J = 3.8 Hz, 6H), 2.32 – 2.23 (m, 1H), 2.11 (s, 1H), 1.91 (s, 2H), 1.78 – 1.41 (m, 12H), 0.97 (s, 3H). ^{13}C NMR (100 MHz, $CDCl_3$) δ 172.3, 172.2, 158.8, 151.1, 149.4, 147.8, 138.1, 135.8, 129.8, 128.8, 116.2, 111.3, 111.3, 110.5, 69.0, 67.7, 64.0, 61.5, 57.0, 54.2, 51.1, 47.8, 42.2, 39.9, 39.9, 35.7, 34.1, 34.0, 29.0, 27.3, 27.1, 24.6, 15.8. HRMS (ESI) calcd for $C_{35}H_{47}ClN_3O_9S$ 720.2716 $[M + H]^+$, found 720.2721.

1.8.18. 4-(2-((5-((1-(2-((1R,3R,4S)-3-(3-chloroprop-1-en-2-yl)-4-methyl-4-vinylcyclohexyl)allyl)piperidin-3-yl)methoxy)-5-oxopentanoyl)oxy)ethoxy)-3-(phenylsulfonyl)-1,2,5-oxadiazole 2-oxide (Vb)

Pale yellow liquid, yield 74%. ^{1}H NMR (400 MHz, $CDCl_3$) δ 8.06 (d, J = 7.5 Hz, 2H), 7.76 (t, J = 7.5 Hz, 1H), 7.63 (t, J = 7.8 Hz, 2H), 5.77 (dd, J = 17.3, 10.9 Hz, 1H), 5.25 (s, 1H), 4.98 – 4.80 (m, 5H), 4.68 – 4.57 (m, 2H), 4.55 – 4.45 (m, 2H), 4.09 (d, J = 11.6 Hz, 1H), 3.94 (dd, J = 21.4, 11.3 Hz, 3H), 2.99 – 2.54 (m, 4H), 2.42 (dt, J = 20.6, 7.3 Hz, 4H), 2.30 – 2.23 (m, 1H), 2.11 (s, 1H), 1.97 (p, J = 7.3 Hz, 4H), 1.76 – 1.36 (m, 10H), 0.97 (s, 3H). ^{13}C NMR (100 MHz, $CDCl_3$) δ 173.0, 172.8, 158.8, 149.4, 147.8, 138.0, 135.8, 129.8, 128.7, 116.2, 111.3, 110.5, 69.0, 67.3, 63.9, 61.2, 57.0, 54.2, 51.1, 47.8, 42.2, 39.9, 39.8, 35.7, 34.0, 34.0, 33.2, 33.1, 27.3, 27.0, 24.6, 20.0, 15.8. HRMS (ESI) calcd for $C_{36}H_{49}ClN_3O_9S$ 734.2873 $[M + H]^+$, found 734.2871.

1.8.19. 4-((4-((4-((1-(2-((1R,3R,4S)-3-(3-chloroprop-1-en-2-yl)-4-methyl-4-vinylcyclohexyl)allyl)piperidin-3-yl)methoxy)-4-oxobutanoyl)oxy)but-2-yn-1-yl)oxy)-3-(phenylsulfonyl)-1,2,5-oxadiazole 2-oxide (Vc)

Pale yellow liquid, yield 70%. ^{1}H NMR (400 MHz, $CDCl_3$) δ 8.07 (d, J = 7.5 Hz, 2H), 7.77 (t, J = 7.5 Hz, 1H), 7.63 (t, J = 7.8 Hz, 2H), 5.78 (dd, J = 17.8, 10.4 Hz, 1H), 5.25 (s, 1H), 5.10 (s, 2H), 4.91 (d, J = 14.0 Hz, 5H), 4.76 (s, 2H), 4.11 – 3.91 (m, 4H), 2.88 (s, 2H), 2.80 – 2.51 (m, J = 3.7 Hz, 7H), 2.31 – 2.21 (m, 1H), 2.12 (s, 1H), 1.92 (s, 3H), 1.71 – 1.39 (m, 10H), 0.97 (s, 3H). ^{13}C NMR (100 MHz, $CDCl_3$) δ 172.2, 171.6, 158.0, 149.4, 147.8, 137.9, 135.9, 129.8, 128.8, 116.2, 111.3, 110.7, 83.9, 78.7, 67.7, 63.9, 58.7, 57.0, 54.2, 52.2, 51.1, 47.8, 42.2, 39.9, 39.8, 35.7, 34.0, 29.0, 28.9, 27.3, 27.0, 24.6, 15.8. HRMS (ESI) calcd for $C_{37}H_{47}ClN_3O_9S$ 744.2716 $[M + H]^+$, found 744.2709.

1.8.20. 4-((4-((5-((1-(2-((1R,3R,4S)-3-(3-chloroprop-1-en-2-yl)-4-methyl-4-vinylcyclohexyl)allyl)piperidin-3-yl)methoxy)-5-oxopentanoyl)oxy)but-2-yn-1-yl)oxy)-3-(phenylsulfonyl)-1,2,5-oxadiazole 2-oxide (Vd)

Pale yellow liquid, yield 73%. ^{1}H NMR (400 MHz, $CDCl_3$) δ 8.07 (d, J = 7.7 Hz, 2H), 7.77 (t, J = 7.5 Hz, 1H), 7.63 (t, J = 7.8 Hz, 2H), 5.78 (dd, J = 17.9, 10.4 Hz, 1H), 5.26 (s, 1H), 5.10 (s, 2H), 4.92 (d, J = 14.0 Hz, 5H), 4.74 (s, 2H), 4.09 (d, J = 11.7 Hz, 1H), 3.94 (dd, J = 18.4, 11.3 Hz, 3H), 3.02 – 2.57 (m, 5H), 2.40 (dt, J = 20.6, 7.3 Hz, 5H), 2.31 – 2.23 (m, 1H), 2.11 (s, 1H), 1.96 (p, J = 7.3 Hz, 4H), 1.70 – 1.42 (m, 10H), 0.97 (s, 3H). ^{13}C NMR (100 MHz, $CDCl_3$) δ 172.9, 172.2, 158.0, 151.1, 149.4, 147.8, 138.0, 135.9, 129.8, 128.8, 116.2, 111.3, 110.7, 84.1, 78.7, 67.4,

64.0, 58.7, 57.0, 54.2, 52.0, 51.1, 47.9, 42.3, 39.9, 39.9, 35.8, 34.1, 33.2, 33.1, 27.3, 27.1, 24.7, 20.1, 15.9. HRMS (ESI) calcd for $C_{38}H_{49}ClN_3O_9S$ 758.2873 [M + H]$^+$, found 758.2897.

1.8.21. 4-(2-((4-(2-(4-(2-((1R,3R,4S)-3-(3-chloroprop-1-en-2-yl)-4-methyl-4-vinylcyclohexyl)allyl)piperazin-1-yl)ethoxy)-4-oxobutanoyl)oxy)ethoxy)-3-(phenylsulfonyl)-1,2,5-oxadiazole 2-oxide (VIa)

Pale yellow liquid, yield 53%. ^{1}H NMR (400 MHz, $CDCl_3$) δ 8.06 (d, J = 7.3 Hz, 2H), 7.75 (t, J = 7.5 Hz, 1H), 7.62 (t, J = 7.8 Hz, 2H), 5.78 (dd, J = 17.8, 10.4 Hz, 1H), 5.26 (s, 1H), 4.98 – 4.83 (m, 5H), 4.62 (dd, J = 5.6, 3.5 Hz, 2H), 4.51 (dd, J = 5.4, 3.6 Hz, 2H), 4.21 (t, J = 6.0 Hz, 2H), 4.08 (d, J = 11.6 Hz, 1H), 3.96 (d, J = 11.7 Hz, 1H), 2.97 – 2.84 (m, 2H), 2.67 (s, 4H), 2.63 (t, J = 6.0 Hz, 2H), 2.46 (d, J = 40.2 Hz, 8H), 2.29 – 2.24 (m, 1H), 2.15 – 2.08 (m, 1H), 1.64 – 1.43 (m, 6H), 0.97 (s, 3H). ^{13}C NMR (100 MHz, $CDCl_3$) δ 172.2, 172.1, 158.8, 150.7, 149.4, 147.9, 138.3, 135.7, 129.8, 128.8, 116.2, 111.2, 111.0, 110.5, 69.0, 63.5, 62.4, 61.5, 56.7, 53.7, 53.2, 51.0, 48.0, 42.4, 39.9, 34.1, 29.1, 29.1, 27.1, 15.9. HRMS (ESI) calcd for $C_{35}H_{48}ClN_4O_9S$ 735.2825 [M + H]$^+$, found 735.2831.

1.8.22. 4-(2-((4-(2-(4-(2-((1R,3R,4S)-3-(3-chloroprop-1-en-2-yl)-4-methyl-4-vinylcyclohexyl)allyl)piperazin-1-yl)ethoxy)-4-oxobutanoyl)oxy)ethoxy)-3-(phenylsulfonyl)-1,2,5-oxadiazole 2-oxide (VIb)

Pale yellow liquid, yield 56%. ^{1}H NMR (400 MHz, $CDCl_3$) δ 8.06 (d, J = 7.3 Hz, 2H), 7.76 (t, J = 7.5 Hz, 1H), 7.62 (t, J = 7.9 Hz, 2H), 5.78 (dd, J = 17.9, 10.4 Hz, 1H), 5.26 (s, 1H), 4.99 – 4.86 (m, 5H), 4.65 – 4.61 (m, 2H), 4.53 – 4.47 (m, 2H), 4.21 (t, J = 5.9 Hz, 2H), 4.09 (d, J = 11.1 Hz, 1H), 3.97 (d, J = 11.7 Hz, 1H), 2.92 (s, 2H), 2.62 (t, J = 5.9 Hz, 2H), 2.59 – 2.36 (m, 12H), 2.30 – 2.24 (m, 1H), 2.16 – 2.09 (m, 1H), 2.01 – 1.95 (m, 2H), 1.81 (s, 2H), 1.64 – 1.41 (m, 6H), 0.98 (s, 3H). ^{13}C NMR (100 MHz, $CDCl_3$) δ 172.9, 172.7, 158.8, 150.7, 149.4, 147.9, 138.3, 135.8, 129.8, 128.8, 116.2, 111.3, 111.0, 110.6, 69.1, 63.5, 62.0, 61.3, 56.8, 53.6, 53.2, 51.1, 48.0, 42.4, 40.0, 34.1, 33.3, 33.2, 29.8, 27.2, 20.1, 15.9. HRMS (ESI) calcd for $C_{36}H_{50}ClN_4O_9S$ 749.2982 [M + H]$^+$, found 749.2993.

1.8.23. 4-((4-((4-(2-(4-(2-((1R,3R,4S)-3-(3-chloroprop-1-en-2-yl)-4-methyl-4-

vinylcyclohexyl)allyl)piperazin-1-yl)ethoxy)-4-oxobutanoyl)oxy)but-2-yn-1-yl)oxy)-3-(phenylsulfonyl)-1,2,5-oxadiazole 2-oxide (VIc)

Pale yellow liquid, yield 60%. ^{1}H NMR (400 MHz, $CDCl_3$) δ 8.07 (d, J = 7.5 Hz, 2H), 7.77 (t, J = 7.5 Hz, 1H), 7.63 (t, J = 7.8 Hz, 2H), 5.78 (dd, J = 17.8, 10.4 Hz, 1H), 5.26 (s, 1H), 5.10 (s, 2H), 4.98 – 4.86 (m, 5H), 4.76 (s, 2H), 4.23 (t, J = 5.9 Hz, 2H), 4.09 (d, J = 11.6 Hz, 1H), 3.97 (d, J = 11.7 Hz, 1H), 2.92 (s, 2H), 2.69 – 2.62 (m, 6H), 2.48 (d, J = 40.6 Hz, 7H), 2.30 – 2.24 (m, 1H), 2.16 – 2.08 (m, 1H), 1.89 (s, 1H), 1.64 – 1.43 (m, 6H), 0.98 (s, 3H). ^{13}C NMR (100 MHz, $CDCl_3$) δ 172.1, 171.6, 158.1, 150.7, 149.4, 147.9, 138.1, 135.8, 129.8, 128.8, 116.2, 111.3, 111.1, 110.7, 84.0, 78.8, 63.5, 62.4, 58.7, 56.7, 53.6, 53.2, 52.2, 51.1, 48.0, 42.4, 40.0, 34.1, 29.1, 29.0, 27.2, 15.9. HRMS (ESI) calcd for $C_{37}H_{48}ClN_4O_9S$ 759.2825 $[M + H]^+$, found 759.2811.

1.8.24. 4-((4-((5-(2-(4-(2-((1R,3R,4S)-3-(3-chloroprop-1-en-2-yl)-4-methyl-4-vinylcyclohexyl)allyl)piperazin-1-yl)ethoxy)-5-oxopentanoyl)oxy)but-2-yn-1-yl)oxy)-3-(phenylsulfonyl)-1,2,5-oxadiazole 2-oxide (VId)

Pale yellow liquid, yield 58%. ^{1}H NMR (400 MHz, $CDCl_3$) δ 8.06 (d, J = 7.5 Hz, 2H), 7.76 (t, J = 7.5 Hz, 1H), 7.63 (t, J = 7.9 Hz, 2H), 5.77 (dd, J = 17.5, 10.7 Hz, 1H), 5.25 (s, 1H), 5.09 (s, 2H), 4.96 – 4.86 (m, 5H), 4.73 (s, 2H), 4.20 (t, J = 5.9 Hz, 2H), 4.08 (d, J = 11.5 Hz, 1H), 3.96 (d, J = 11.7 Hz, 1H), 2.91 (s, 2H), 2.62 (t, J = 5.9 Hz, 2H), 2.42 (dt, J = 20.1, 7.3 Hz, 12H), 2.29 – 2.24 (m, 1H), 2.13 – 2.07 (m, 1H), 1.98 – 1.91 (m, 2H), 1.63 – 1.41 (m, 6H), 0.96 (s, 3H). ^{13}C NMR (100 MHz, $CDCl_3$) δ 172.8, 172.1, 157.9, 150.5, 149.3, 147.7, 137.8, 135.8, 129.8, 128.7, 116.2, 111.2, 111.0, 110.6, 84.0, 78.6, 63.3, 61.8, 58.6, 56.7, 53.5, 53.0, 51.9, 51.1, 47.6, 42.1, 39.8, 39.7, 33.9, 33.1, 32.9, 27.0, 19.9, 15.7. HRMS (ESI) calcd for $C_{38}H_{50}ClN_4O_9S$ 773.2982 $[M + H]^+$, found 773.2974.

4.2. NO-releasing test

The NO-releasing test was performed using a NO Determination Kit (Microplate Method), purchased from Nanjing Jiancheng Institute of Biological Engineering. Different concentrations of sodium nitrite solutions were prepared to obtain a calibration curve. 0.1 mM of each compound in phosphate buffer solution (PBS) containing 2% dimethyl sulfoxide and 5.0 mM L-cysteine at pH 7.4 was incubated at

37 ^{o}C for 10–150 min and were sampled every 15 min for 120 min and then every 30 min for the remaining time. The collected 160 uL samples were mixed with 0.08 mL of color developing agent and incubated at 37 ^{o}C for 15 min, then measured at 550 nm [17].

4.3 Cell culture

H520, SW620, U87MG and HFL-1 cells were purchased from Nanjing Key Gen Biotech Co. Ltd. (Nanjing, China), which were cultured in RPMI-1640, L-15, DMEM and Ham's F12K medium, respectively, supplemented with 10%fetal bovine serum (FBS), 100 units/mL penicillin, and 100 g/mL streptomycin at 37 ^{o}C in 5%CO_2 atmosphere.

4.4. Anti-proliferative assay

The anti-proliferative activity of the compounds was determined by MTT assay. 100 μL H520, SW620, U87MG and HFL-1 cell suspensions (~5.0×10^4 cell/mL) were added to a 96-well cell culture plate and incubated for 24 h at 37 °C under an atmosphere of 5% CO_2. β-Elemene NO donor hybrids were dissolved in the culture medium with 0.5% DMSO at different concentrations and treated to the cells for another 72 h. Then MTT (5 mg/mL) PBS solution was added and incubated for another 4 h. The IC_{50} values were calculated according to the dose-dependent curves. All the tests were repeated in three independent experiments [8].

4.5. U87MG orthotopic glioma model

BABLc female nude mice were anesthetized and fixed on a stereotactic frame. The skin of the head was disinfected with an alcohol swab and cut slightly to the right along the midline of the brain. The parietal bone was first exposed and drilled a burr hole in the right 2 mm and posterior 1 mm of the bregma. The needle was slowly injected into the cranial cavity of mice vertically at a depth of 3.5 mm, then withdrawn 0.5 mm and stop for 1 min, and slowly injected the luciferase transfected

U87MG cells suspension (5 X 10^7 cells/mL, 20 μL). After the injection, stayed for 1 min more and slowly withdrew the needle. Sterilized the skin with alcohol swabs, sutured the skin and put the mice back into the cage to wake up naturally [18].

After 14 days of cell inoculation, the animals were randomly divided into 3 groups of 5 animals. At the same time, the nude mice in each group were administrated saline, β-elemene (60 mg/kg) and compound **Id** (60 mg/kg) intraperitoneally for three weeks. Mice in each group were intraperitoneally injected *D*-luciferin (100 μL/mouse)(Nanjing Key Gen Biotech Co. Ltd., KGAF040) each week after administration. After 10 min, the mice were anesthetized with isoflurane and placed on the stage of the dark instrument box and performed the *in vivo* imaging detection to observe the fluorescence of tumors in the brain of mice with IVIS® Spectrum (PerkinElmer). At the end of the experiment, the mice were sacrificed immediately, the brain tissues were surgically stripped, photographed and weighed.

4.6 Histological analysis

The tissue samples were first deparaffinized. The tissues sections were soaked in xylene for 5 min and soaked for another 5 minutes after replacing xylene. Then the samples were dipped in absolute ethanol (5 min), followed by 95% ethanol (5 min), 85% ethanol (5 min), 70% ethanol (5 min) and PBS (3 min×3 times). Finally, the samples were H & E stained for further microscopic analysis.

5. ^{1}H and ^{13}C NMR spectra of compounds Ia-d, IIa-b, IIIa-d, IVa-d, Va-b, and VIa-b

4-(2-((4-(2-((2-((1R,3R,4S)-3-(3-chloroprop-1-en-2-yl)-4-methyl-4-vinylcyclohexyl)allyl)(methyl)amino)ethoxy)-4-oxobutanoyl)oxy)ethoxy)-3-(phenylsulfonyl)-1,2,5-oxadiazole 2-oxide (Ia)

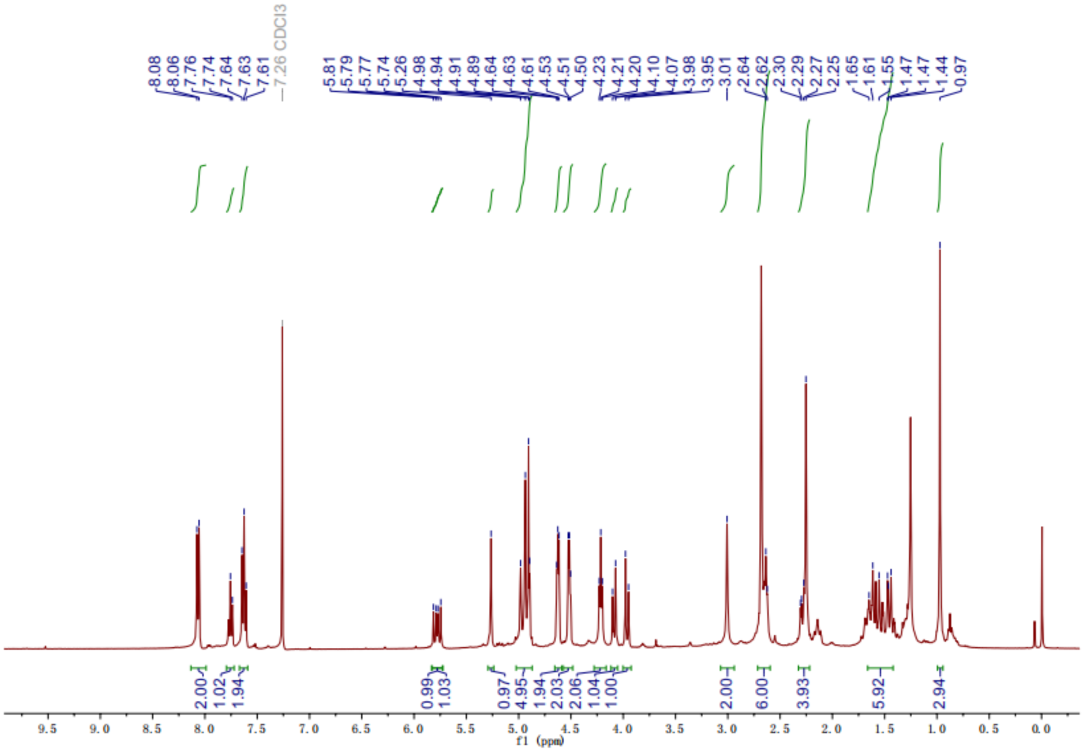

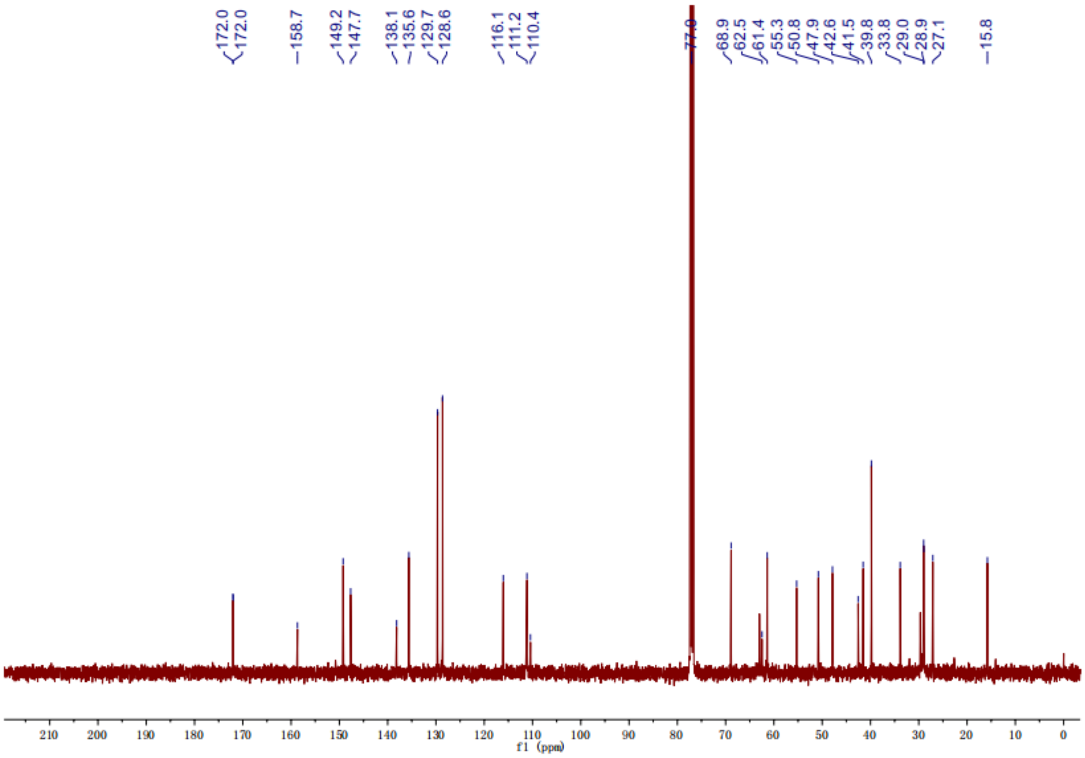

172.0
172.0
158.7
149.2
147.7
138.1
135.6
129.7
128.6
116.1
111.2
110.4
77.0
68.9
62.5
61.4
55.3
50.8
47.9
42.6
41.5
39.8
33.8
29.0
28.9
27.1
15.8
210 200 190 180 170 160 150 140 130 120 110 100 90 80 70 60 50 40 30 20 10 0
f1 (ppm)

4-(2-((5-(2-((2-((1R,3R,4S)-3-(3-chloroprop-1-en-2-yl)-4-methyl-4-vinylcyclohexyl)allyl)(methyl)amino)ethoxy)-5-oxopentanoyl)oxy)ethoxy)-3-(phenylsulfonyl)-1,2,5-oxadiazole 2-oxide (Ib)

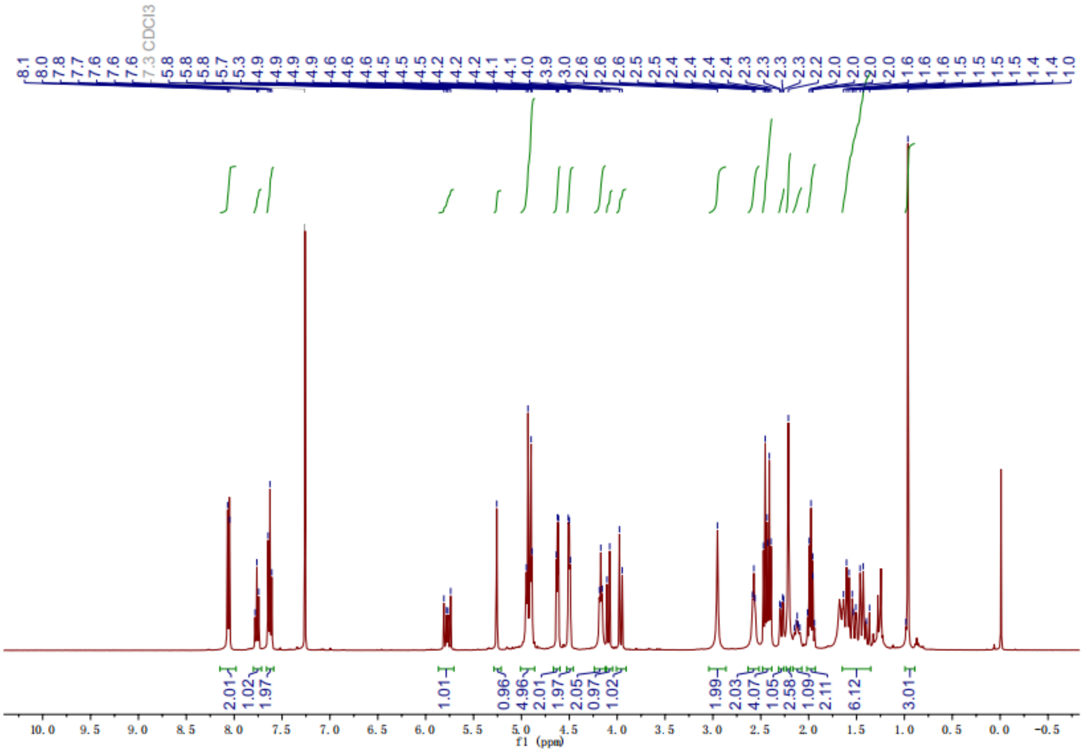

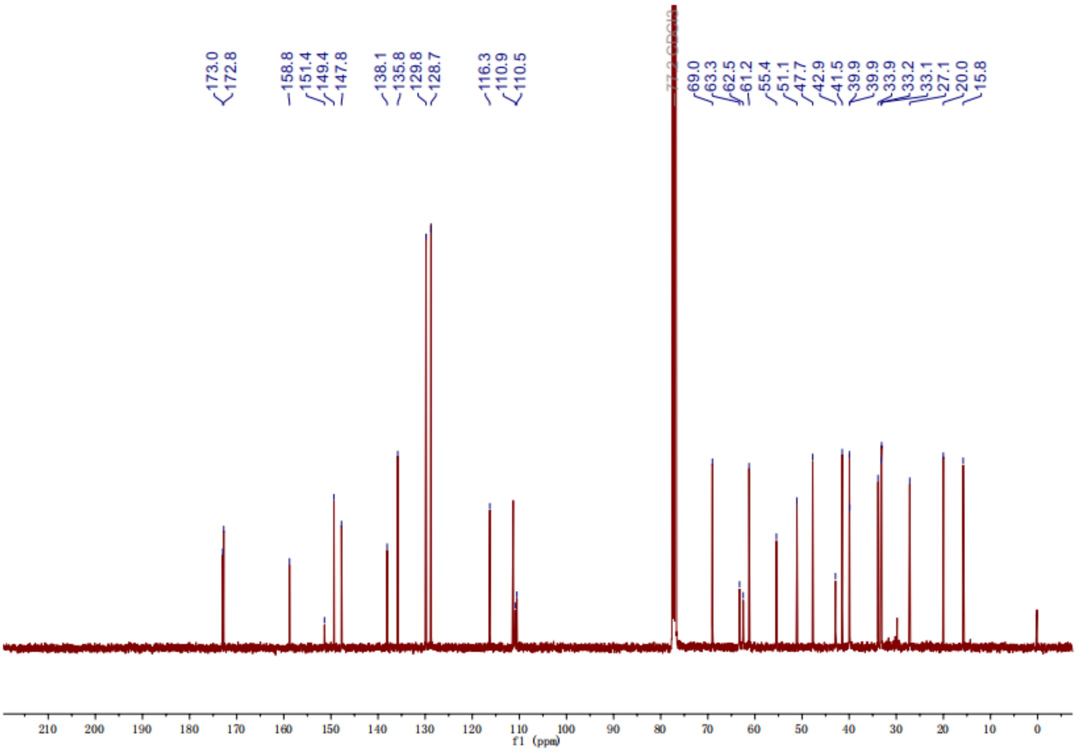

4-((4-((4-(2-((2-((1R,3R,4S)-3-(3-chloroprop-1-en-2-yl)-4-methyl-4-vinylcyclohexyl)allyl)(methyl)amino)ethoxy)-4-oxobutanoyl)oxy)but-2-yn-1-yl)oxy)-3-(phenylsulfonyl)-1,2,5-oxadiazole 2-oxide (Ic)

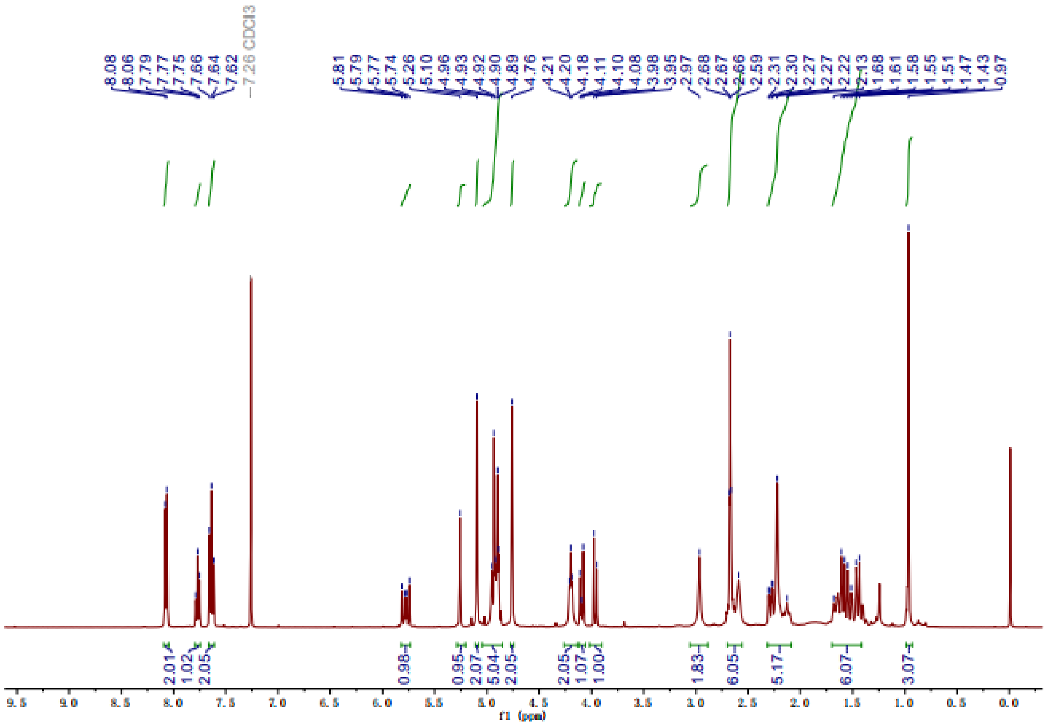

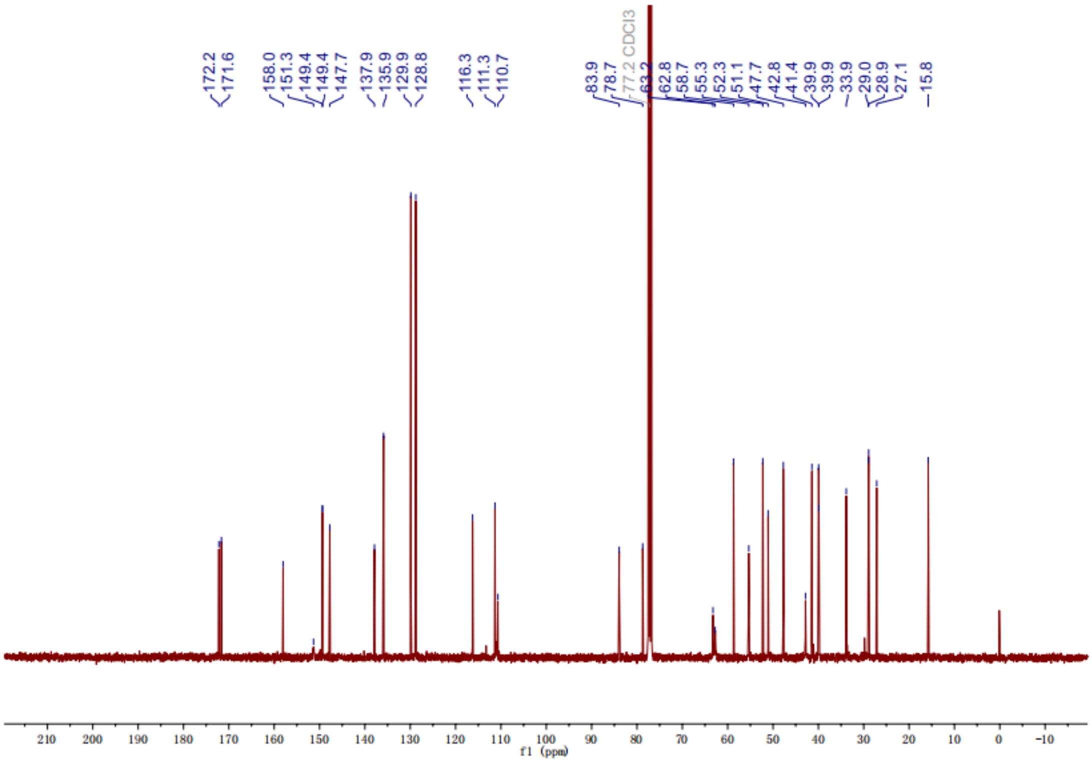

4-((4-((5-(2-((2-((1R,3R,4S)-3-(3-chloroprop-1-en-2-yl)-4-methyl-4-vinylcyclohexyl)allyl)(methyl)amino)ethoxy)-5-oxopentanoyl)oxy)but-2-yn-1-yl)oxy)-3-(phenylsulfonyl)-1,2,5-oxadiazole 2-oxide (Id)

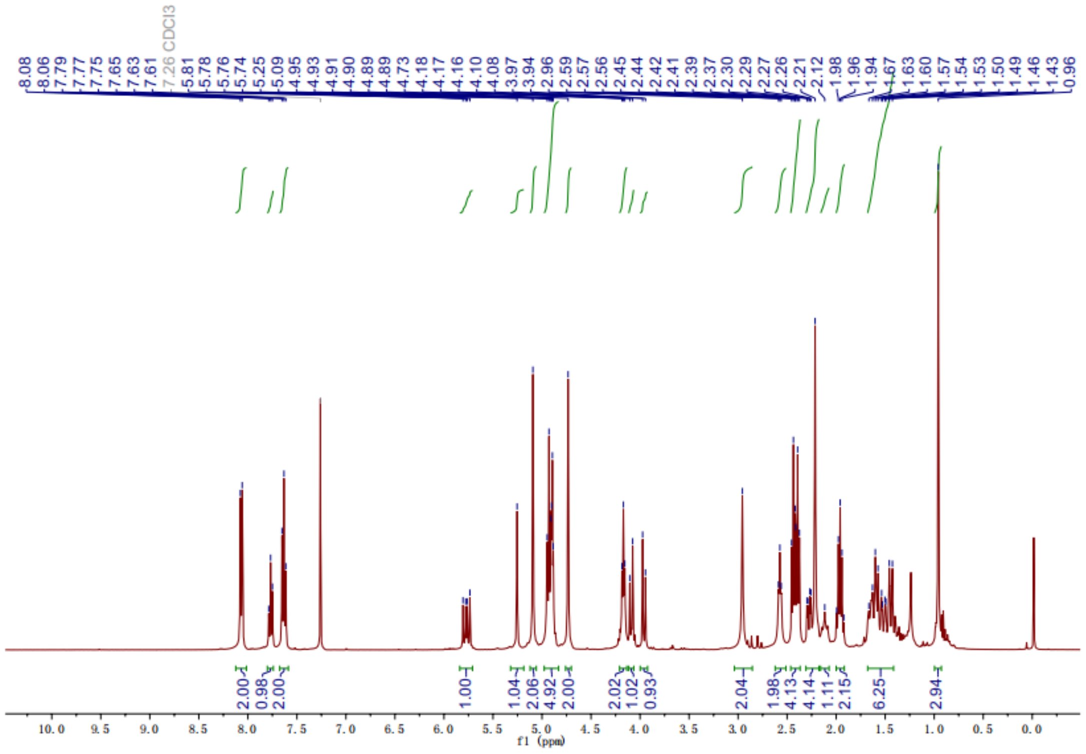

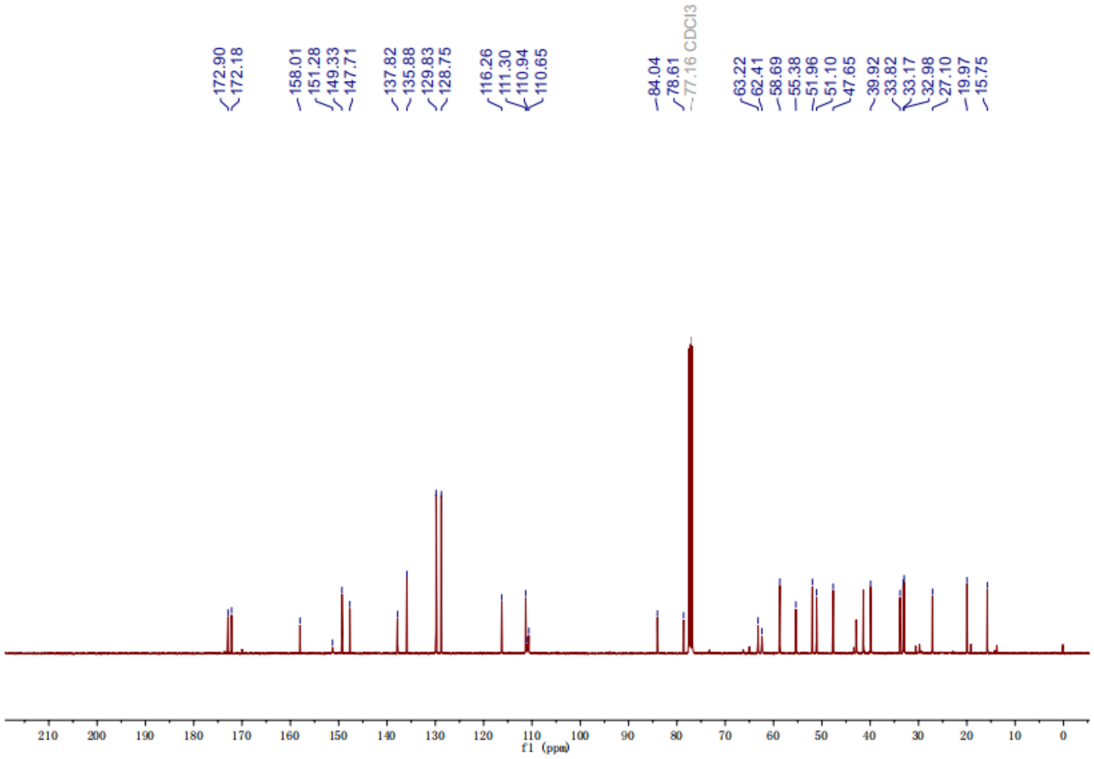

4-(2-((4-(2-((2-((1R,3R,4S)-3-(3-chloroprop-1-en-2-yl)-4-methyl-4-vinylcyclohexyl)allyl)(isopropyl)amino)ethoxy)-4-oxobutanoyl)oxy)ethoxy)-3-(phenylsulfonyl)-1,2,5-oxadiazole 2-oxide (IIa)

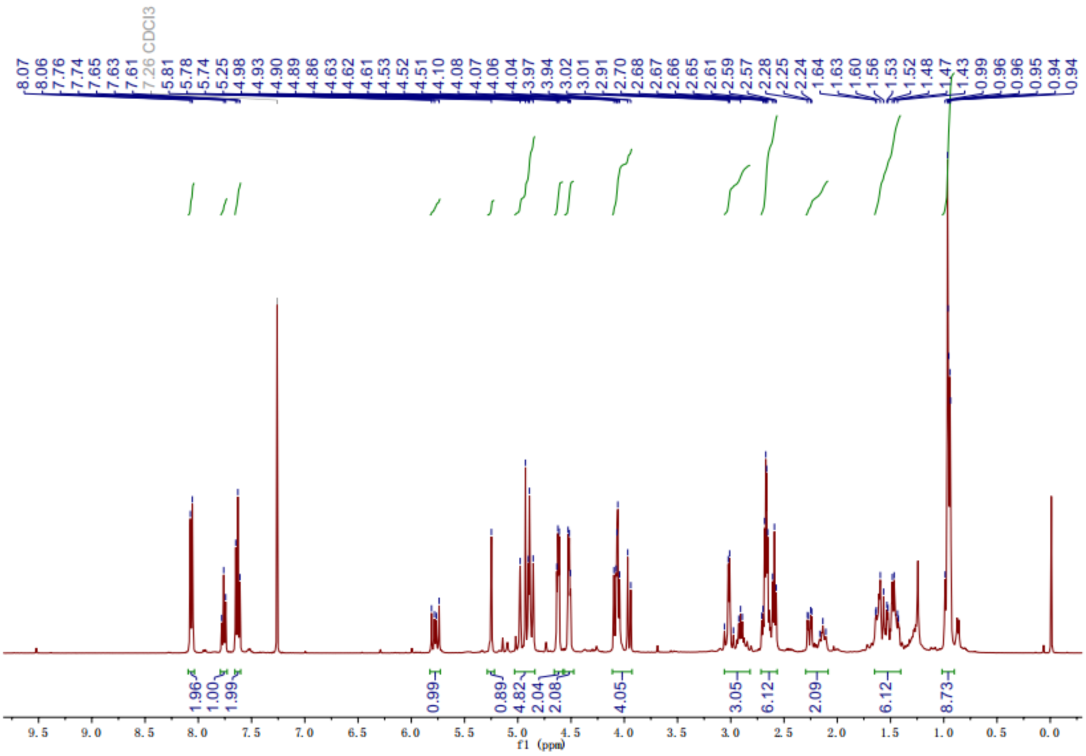

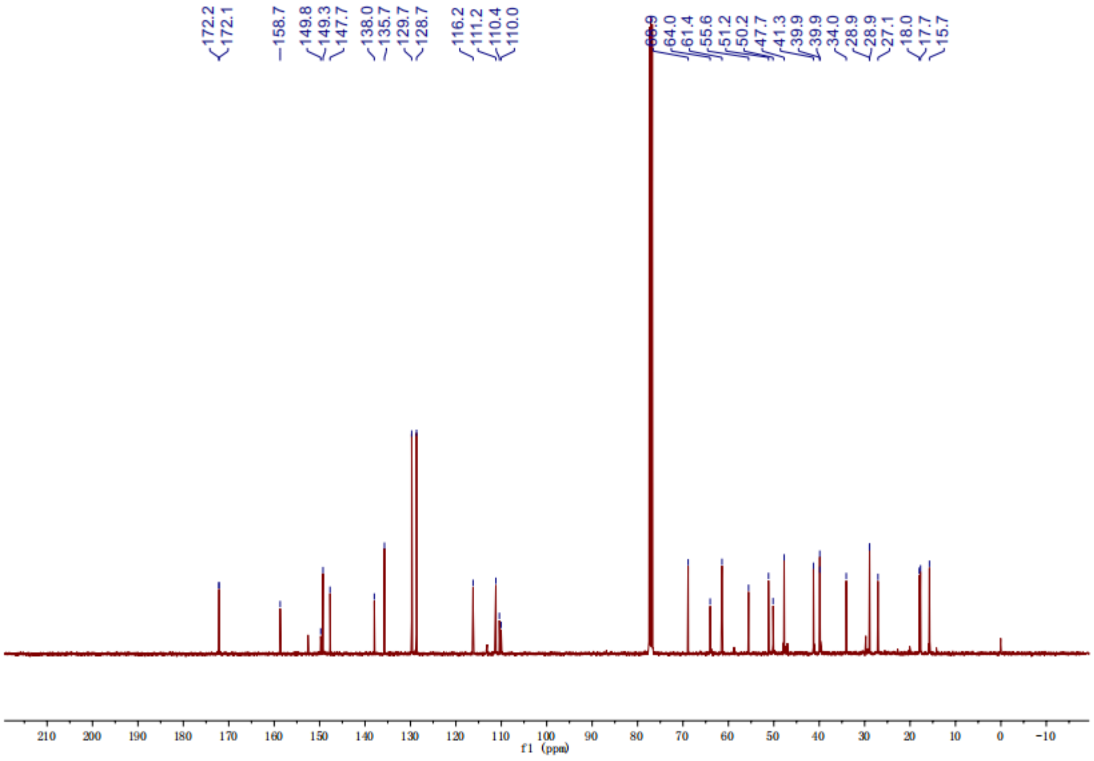

4-(2-((5-(2-((2-((1R,3R,4S)-3-(3-chloroprop-1-en-2-yl)-4-methyl-4-vinylcyclohexyl)allyl)(isopropyl)amino)ethoxy)-5-oxopentanoyl)oxy)ethoxy)-3-(phenylsulfonyl)-1,2,5-oxadiazole 2-oxide (IIb)

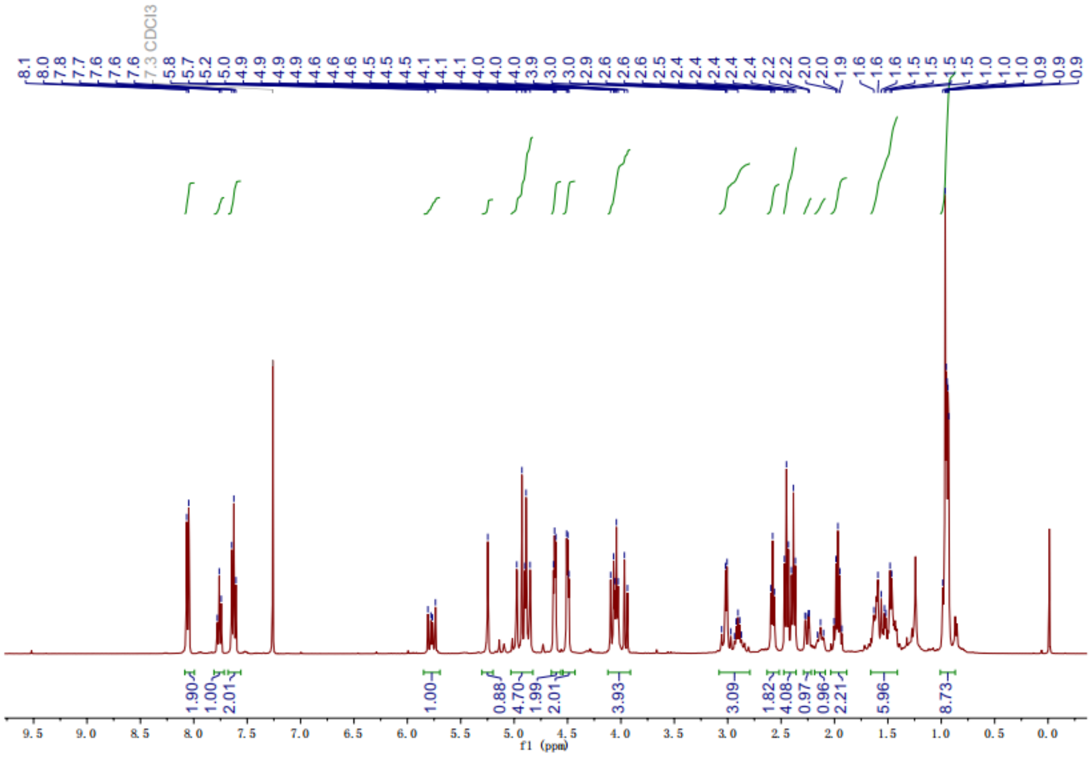

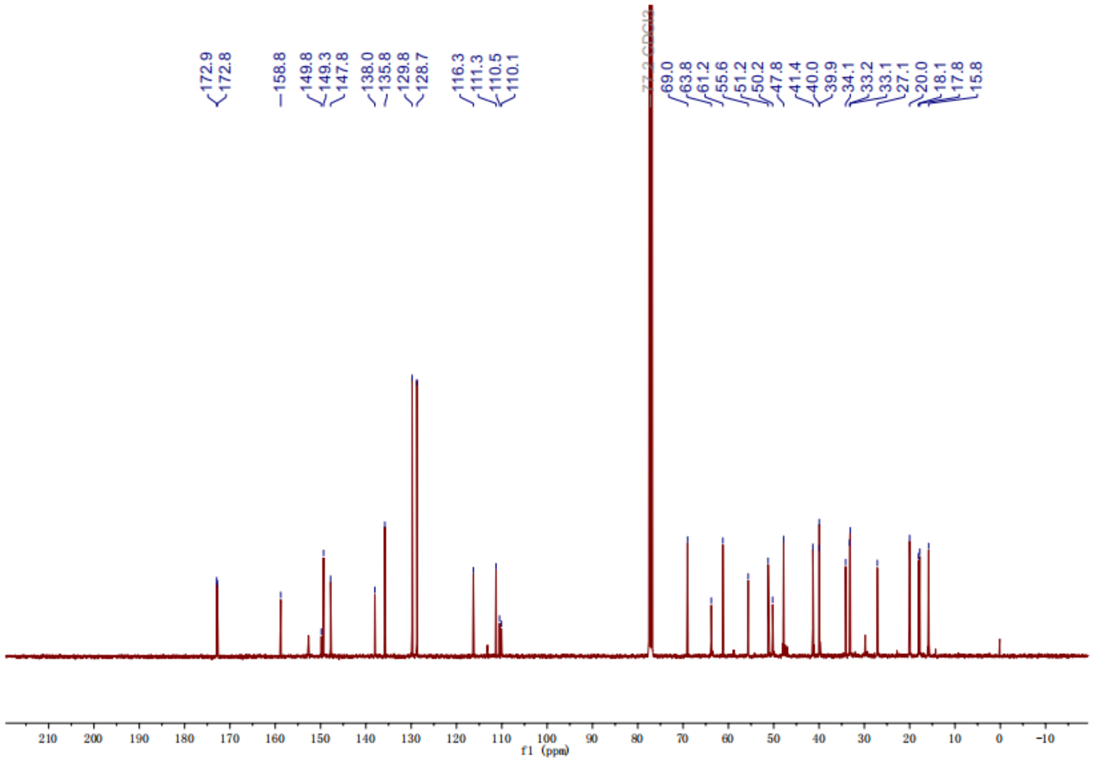

4-((4-((4-(2-((2-((1R,3R,4S)-3-(3-chloroprop-1-en-2-yl)-4-methyl-4-vinylcyclohexyl)allyl)(isopropyl)amino)ethoxy)-4-oxobutanoyl)oxy)but-2-yn-1-yl)oxy)-3-(phenylsulfonyl)-1,2,5-oxadiazole 2-oxide (IIc)

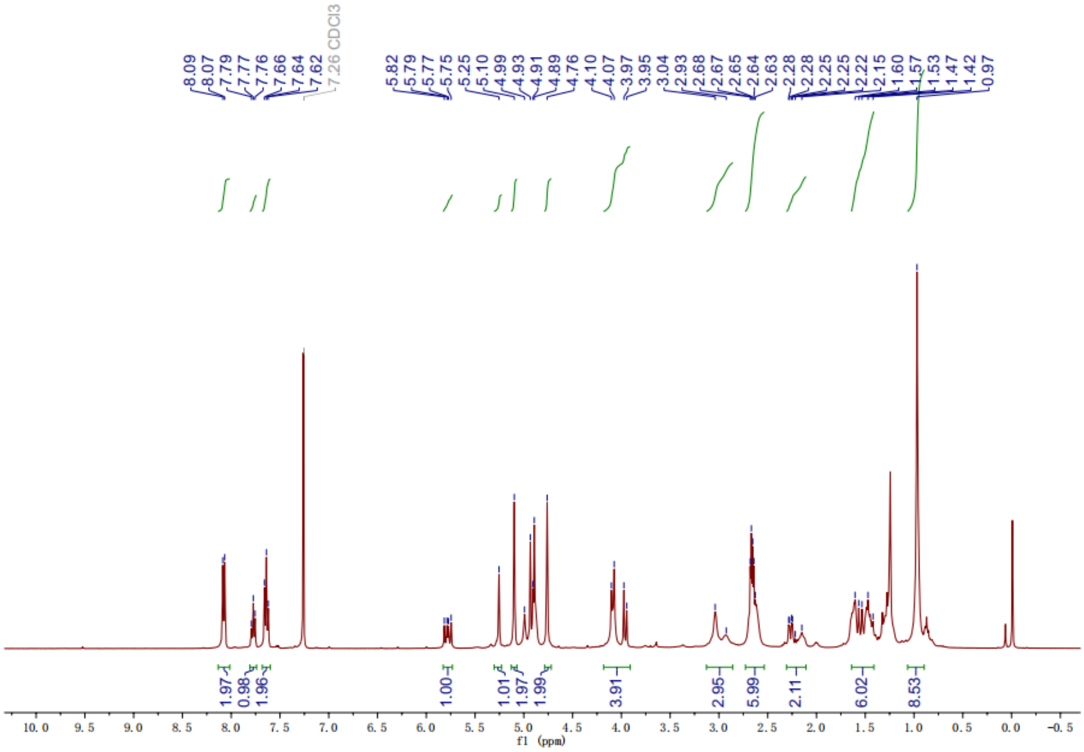

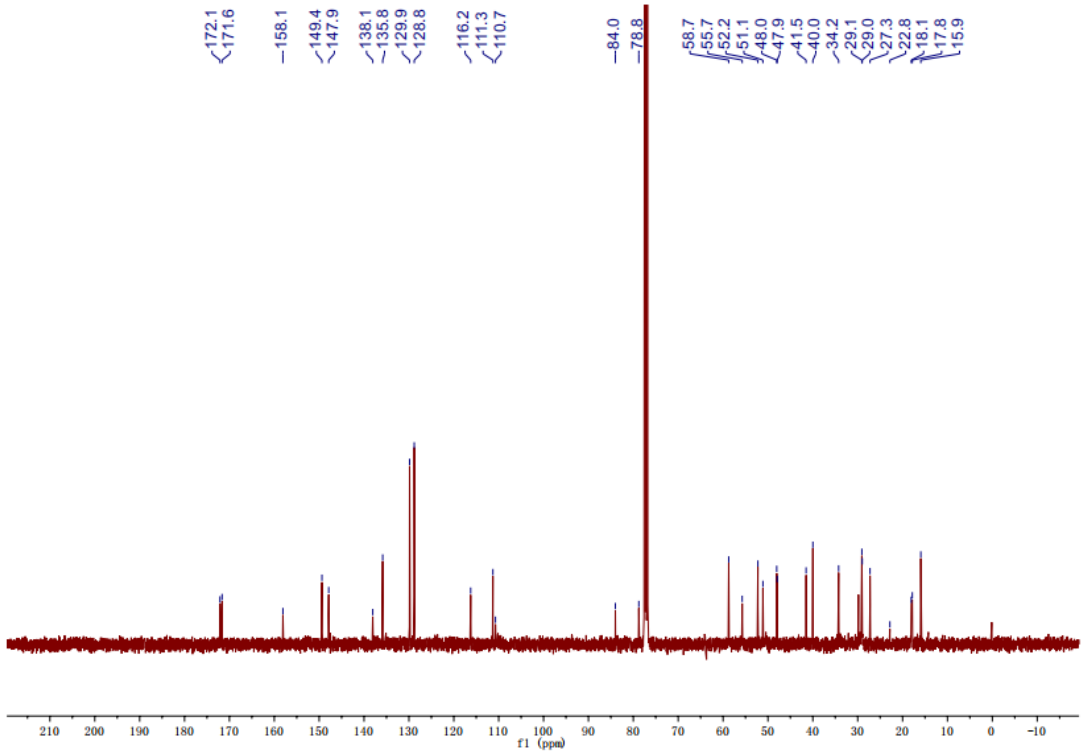

4-((4-((5-(2-((2-((1R,3R,4S)-3-(3-chloroprop-1-en-2-yl)-4-methyl-4-vinylcyclohexyl)allyl)(isopropyl)amino)ethoxy)-5-oxopentanoyl)oxy)but-2-yn-1-yl)oxy)-3-(phenylsulfonyl)-1,2,5-oxadiazole 2-oxide (IId)

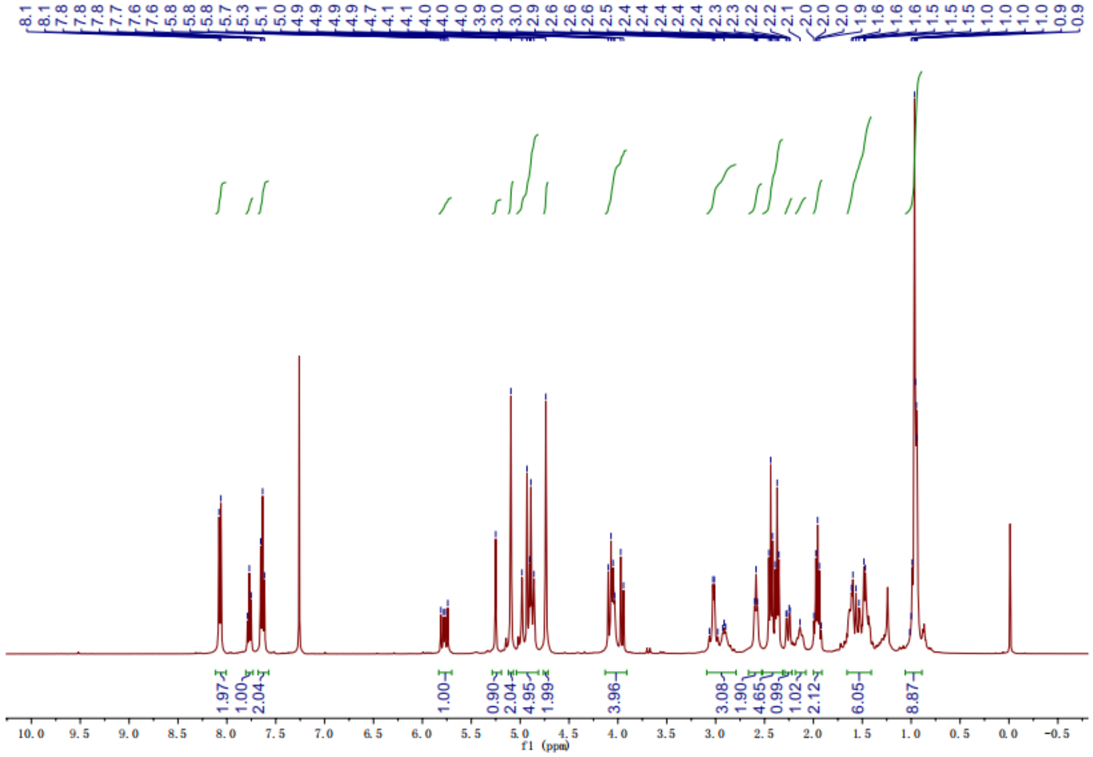

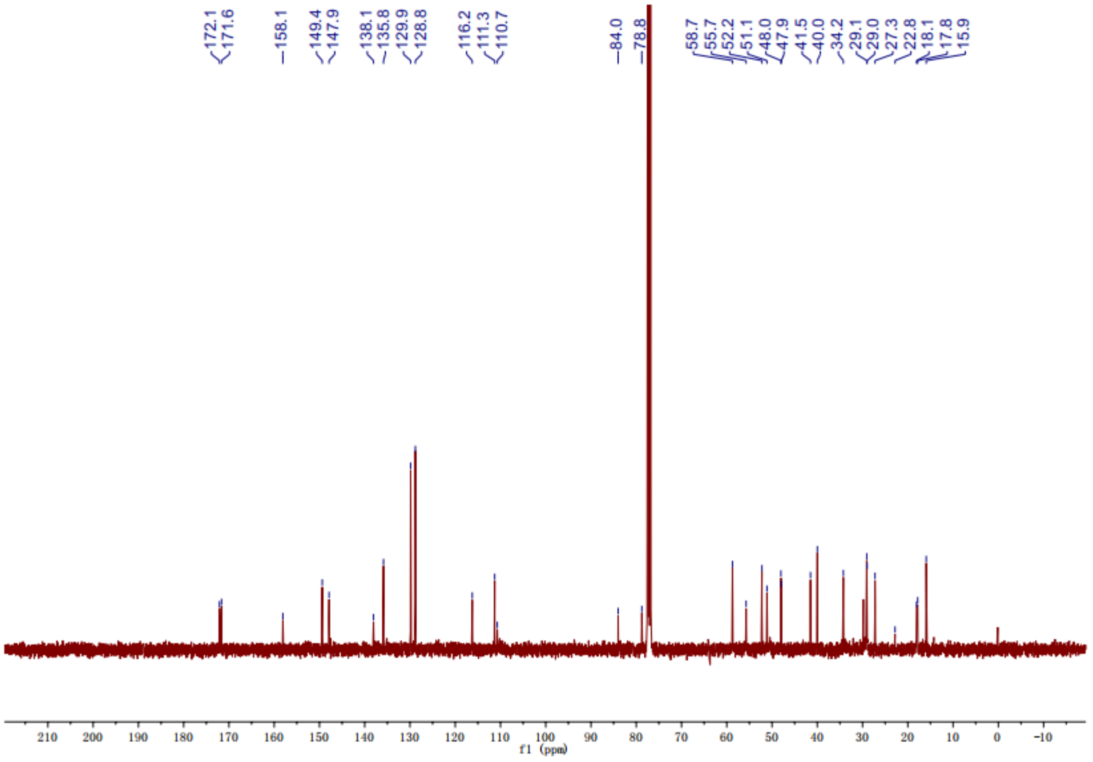

4-(2-((4-(((S)-1-(2-((1R,3R,4S)-3-(3-chloroprop-1-en-2-yl)-4-methyl-4-vinylcyclohexyl)allyl)pyrrolidin-3-yl)oxy)-4-oxobutanoyl)oxy)ethoxy)-3-(phenylsulfonyl)-1,2,5-oxadiazole 2-oxide (IIIa)

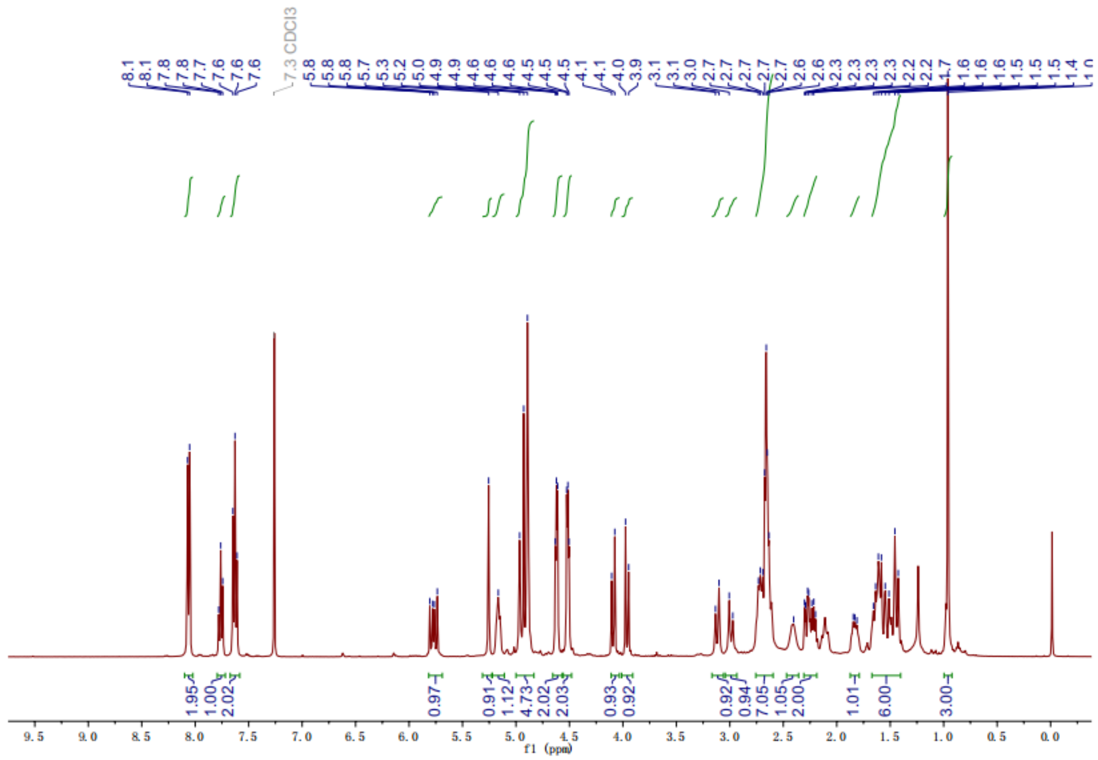

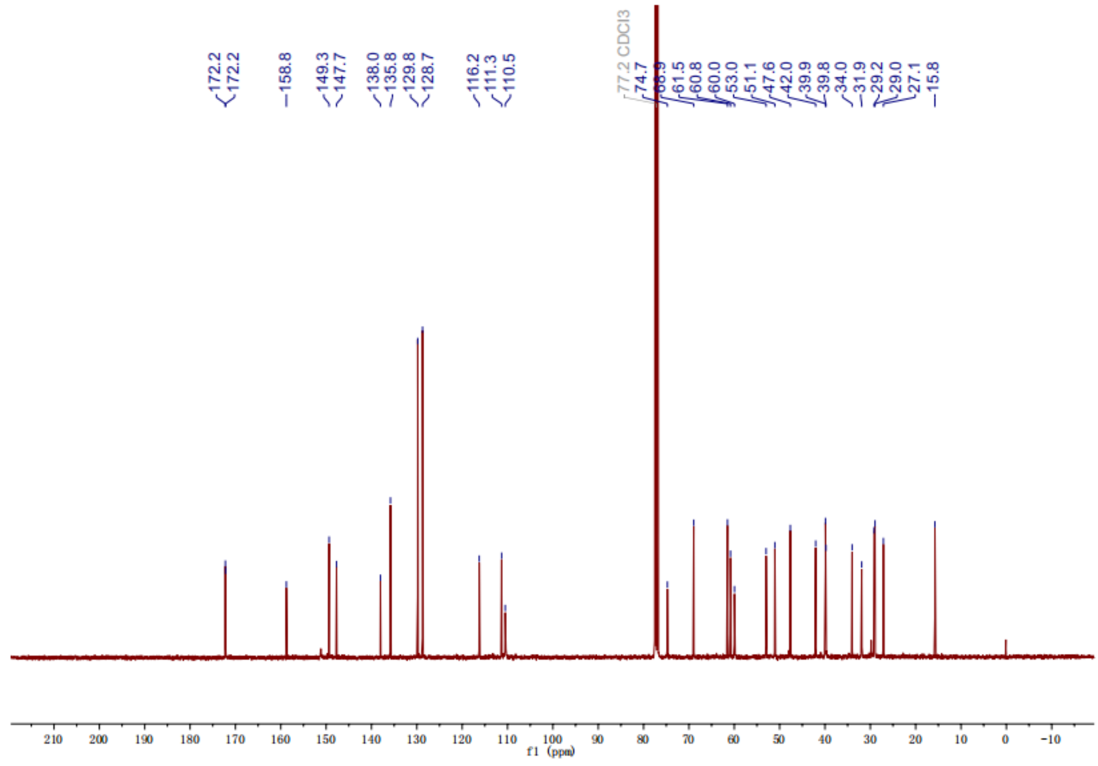

4-(2-((5-(((S)-1-(2-((1R,3R,4S)-3-(3-chloroprop-1-en-2-yl)-4-methyl-4-vinylcyclohexyl)allyl)pyrrolidin-3-yl)oxy)-5-oxopentanoyl)oxy)ethoxy)-3-(phenylsulfonyl)-1,2,5-oxadiazole 2-oxide (IIIb)

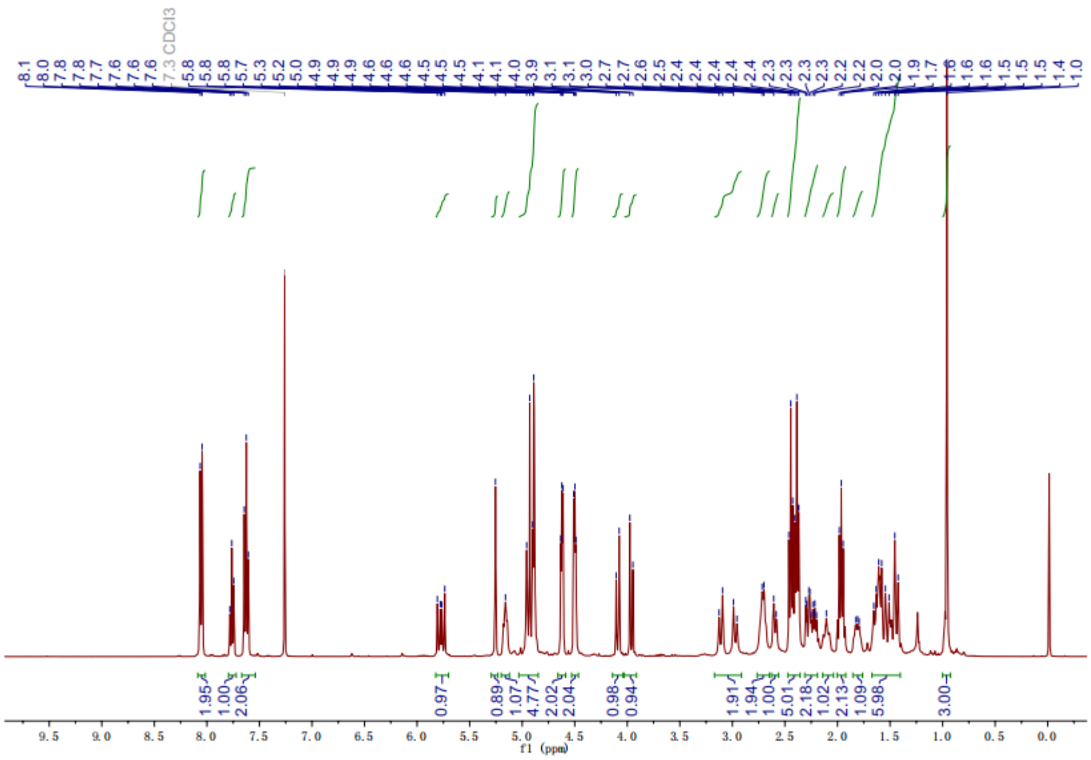

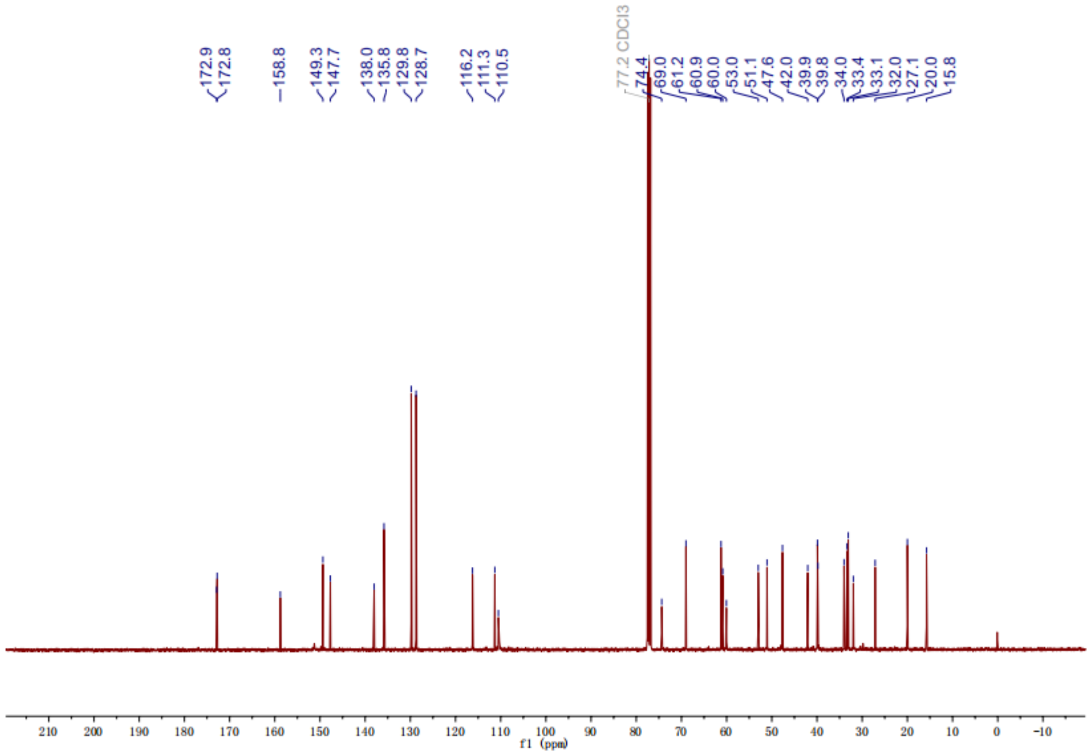

4-((4-((4-(((S)-1-(2-((1R,3R,4S)-3-(3-chloroprop-1-en-2-yl)-4-methyl-4-vinylcyclohexyl)allyl)pyrrolidin-3-yl)oxy)-4-oxobutanoyl)oxy)but-2-yn-1-yl)oxy)-3-(phenylsulfonyl)-1,2,5-oxadiazole 2-oxide (IIIc)

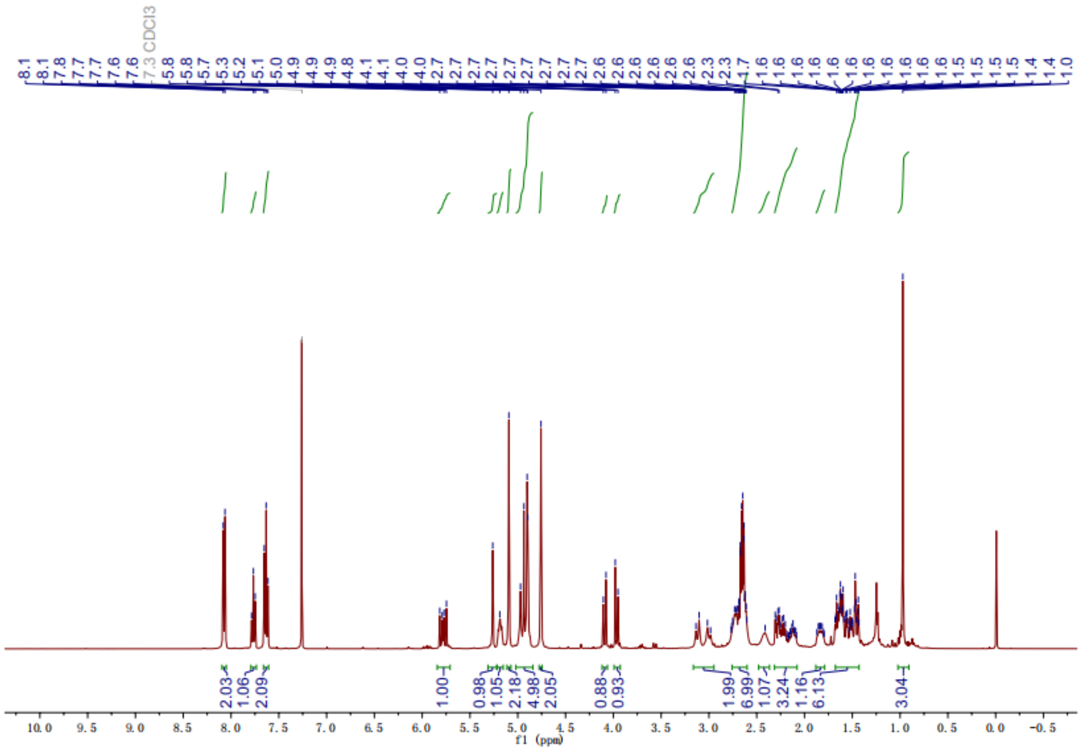

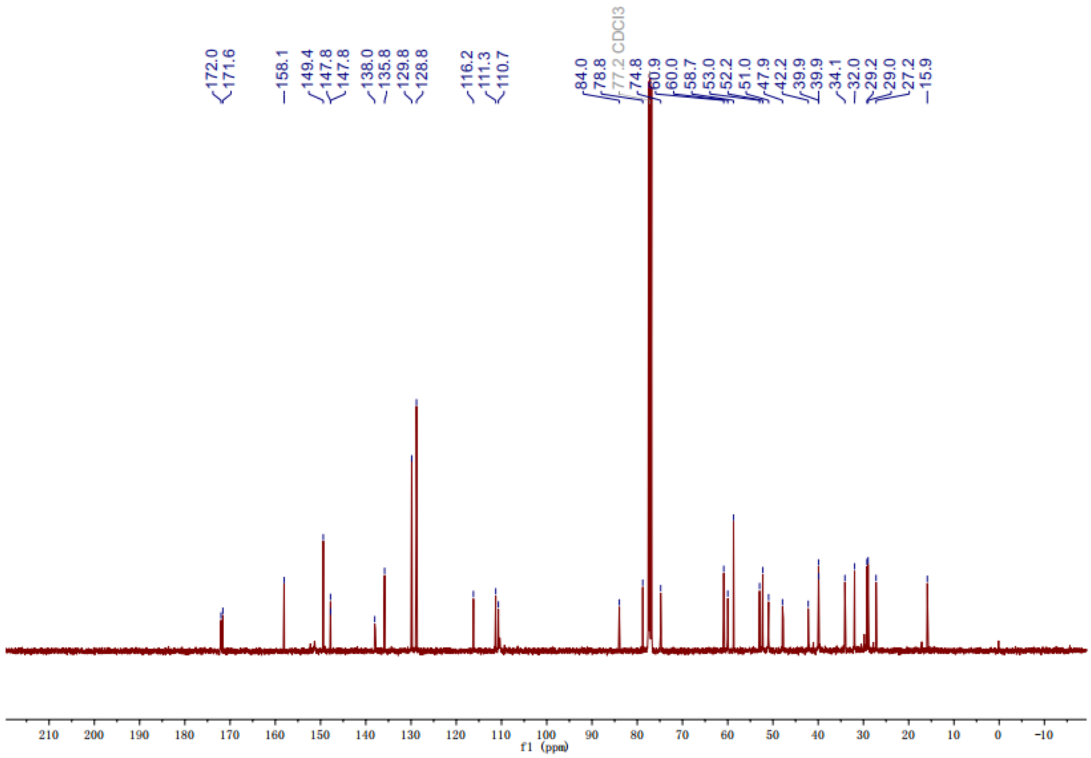

4-((4-((5-(((S)-1-(2-((1R,3R,4S)-3-(3-chloroprop-1-en-2-yl)-4-methyl-4-vinylcyclohexyl)allyl)pyrrolidin-3-yl)oxy)-5-oxopentanoyl)oxy)but-2-yn-1-yl)oxy)-3-(phenylsulfonyl)-1,2,5-oxadiazole 2-oxide (IIId)

5-

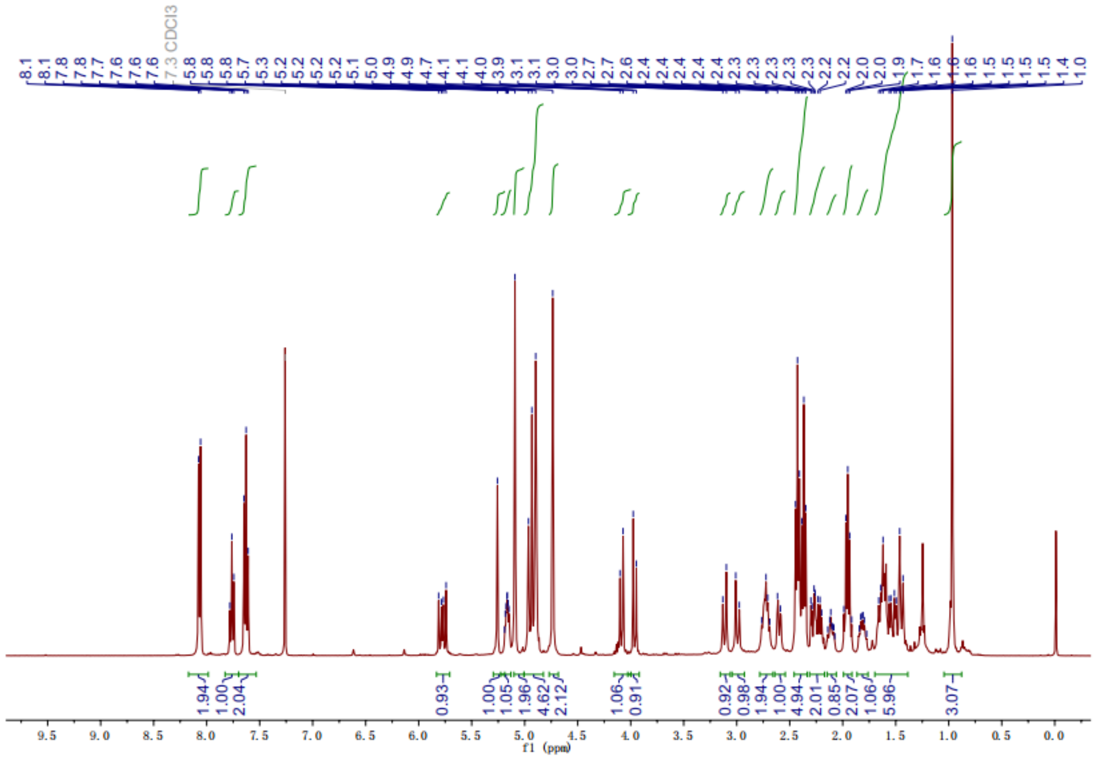

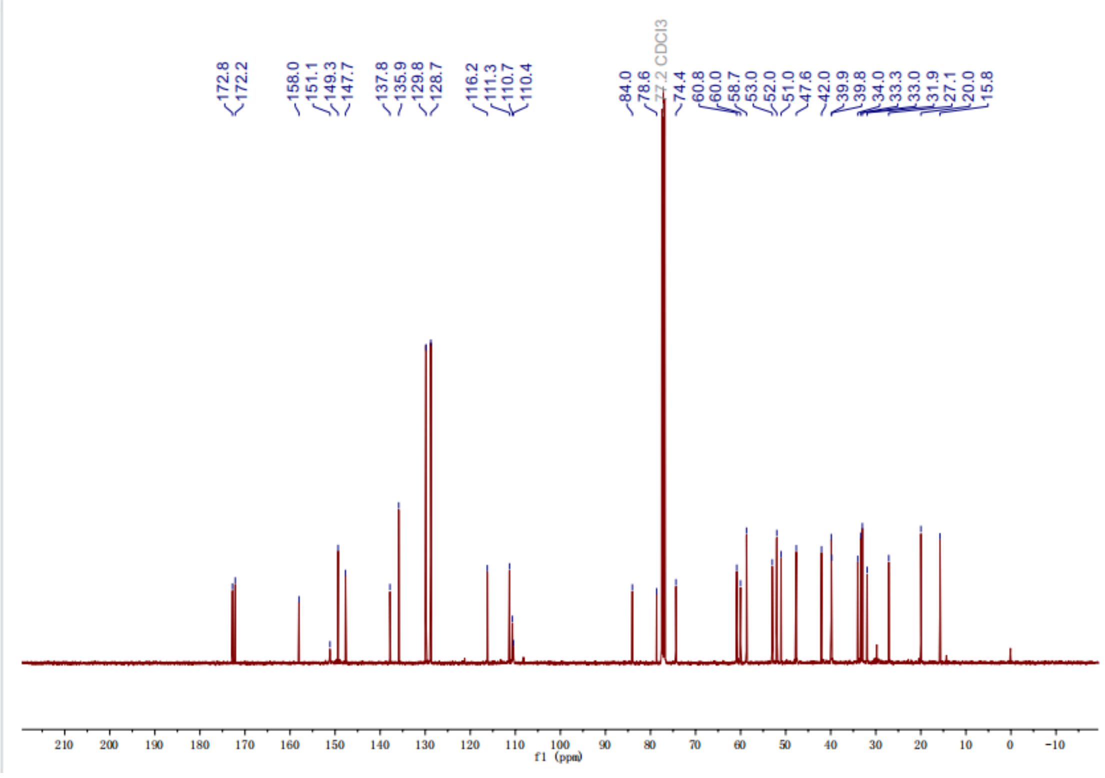

4-(2-((4-((1-(2-((1R,3R,4S)-3-(3-chloroprop-1-en-2-yl)-4-methyl-4-vinylcyclohexyl)allyl)piperidin-4-yl)methoxy)-4-oxobutanoyl)oxy)ethoxy)-3-(phenylsulfonyl)-1,2,5-oxadiazole 2-oxide (IVa)

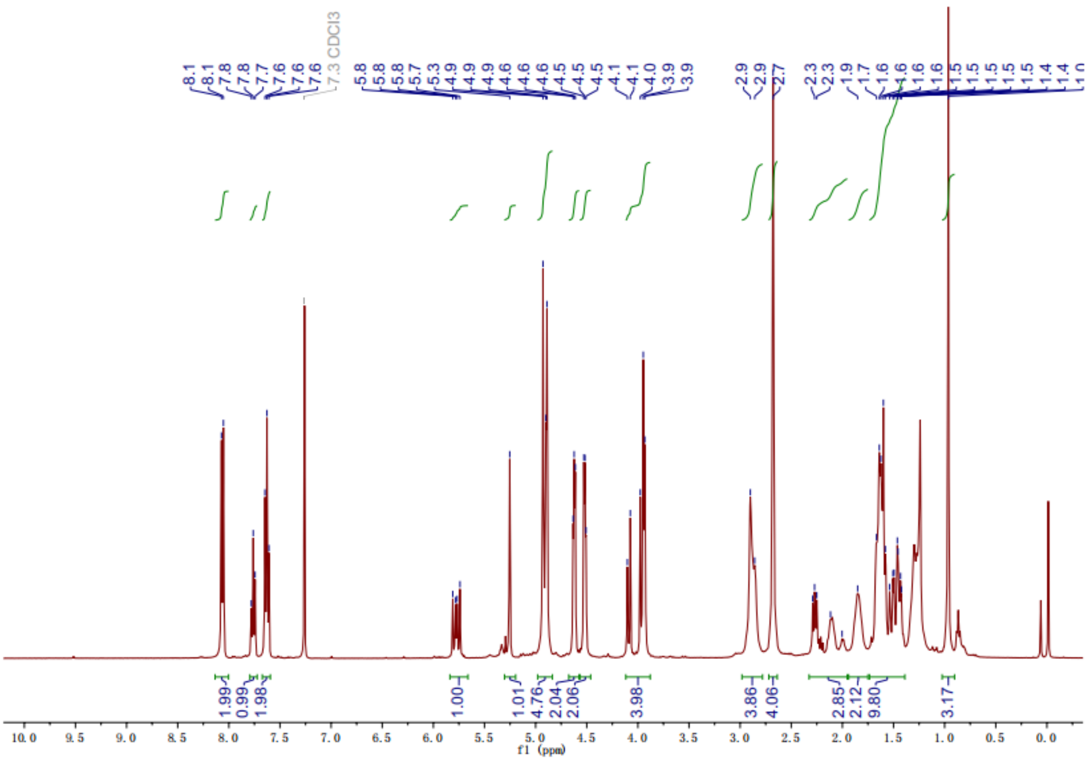

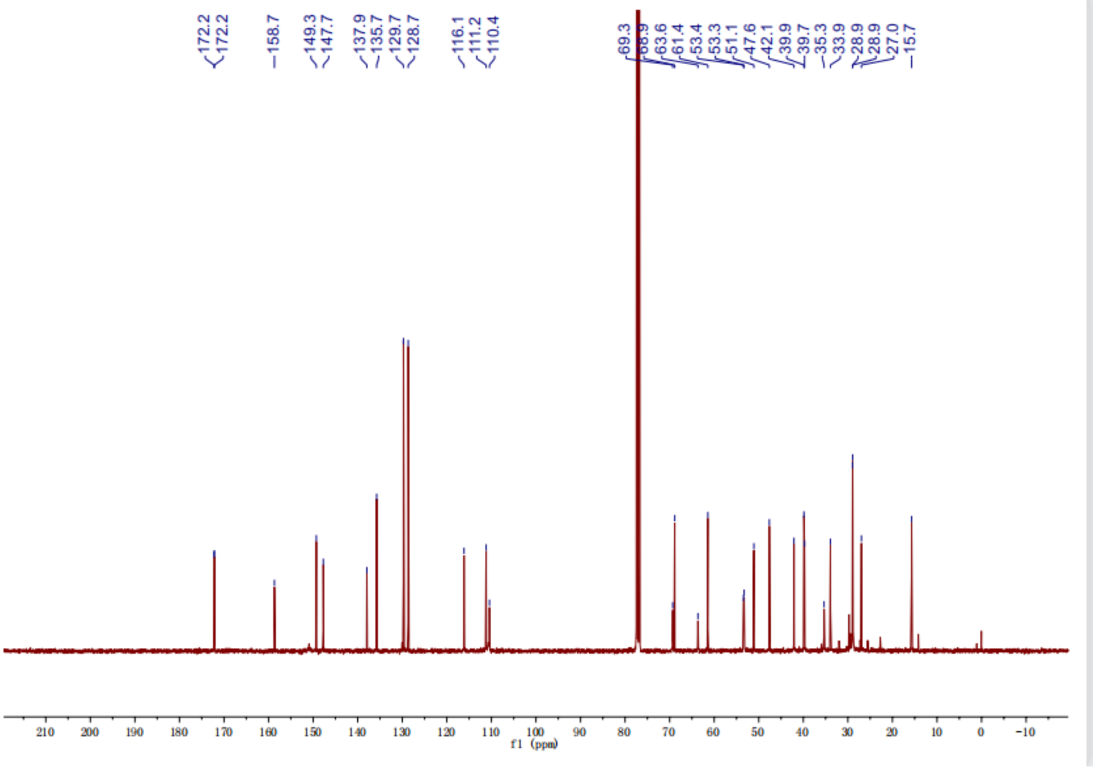

4-(2-((5-((1-(2-((1R,3R,4S)-3-(3-chloroprop-1-en-2-yl)-4-methyl-4-vinylcyclohexyl)allyl)piperidin-4-yl)methoxy)-5-oxopentanoyl)oxy)ethoxy)-3-(phenylsulfonyl)-1,2,5-oxadiazole 2-oxide (IVb)

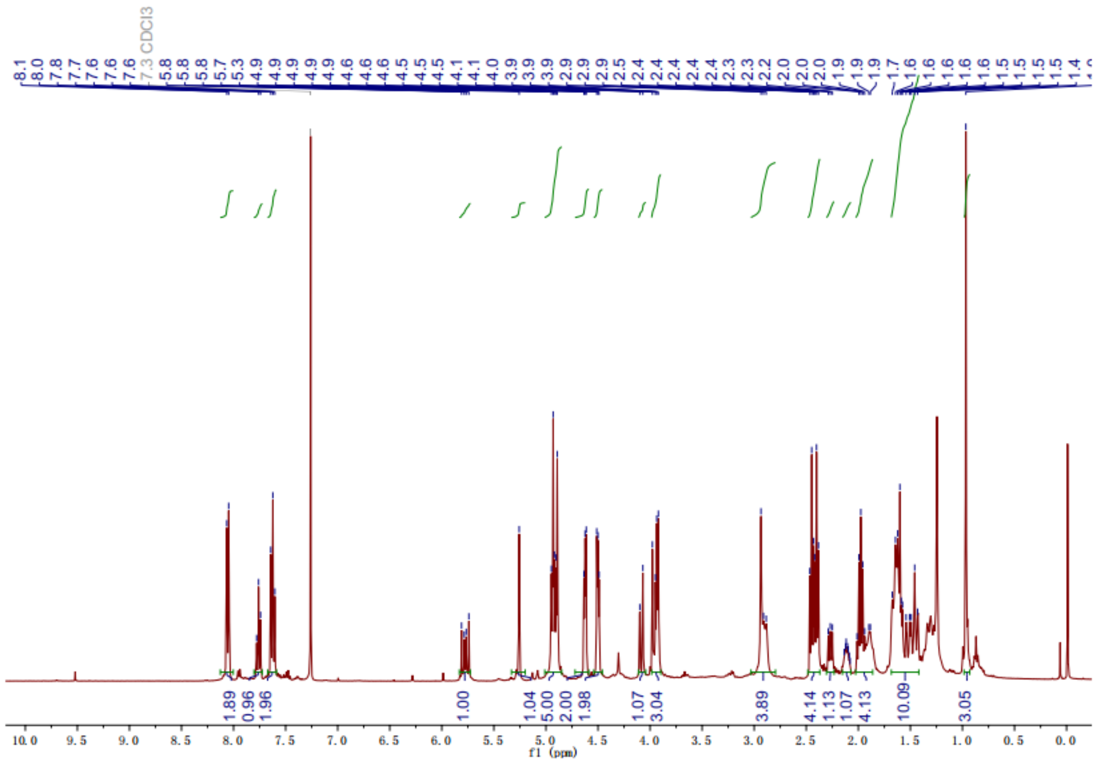

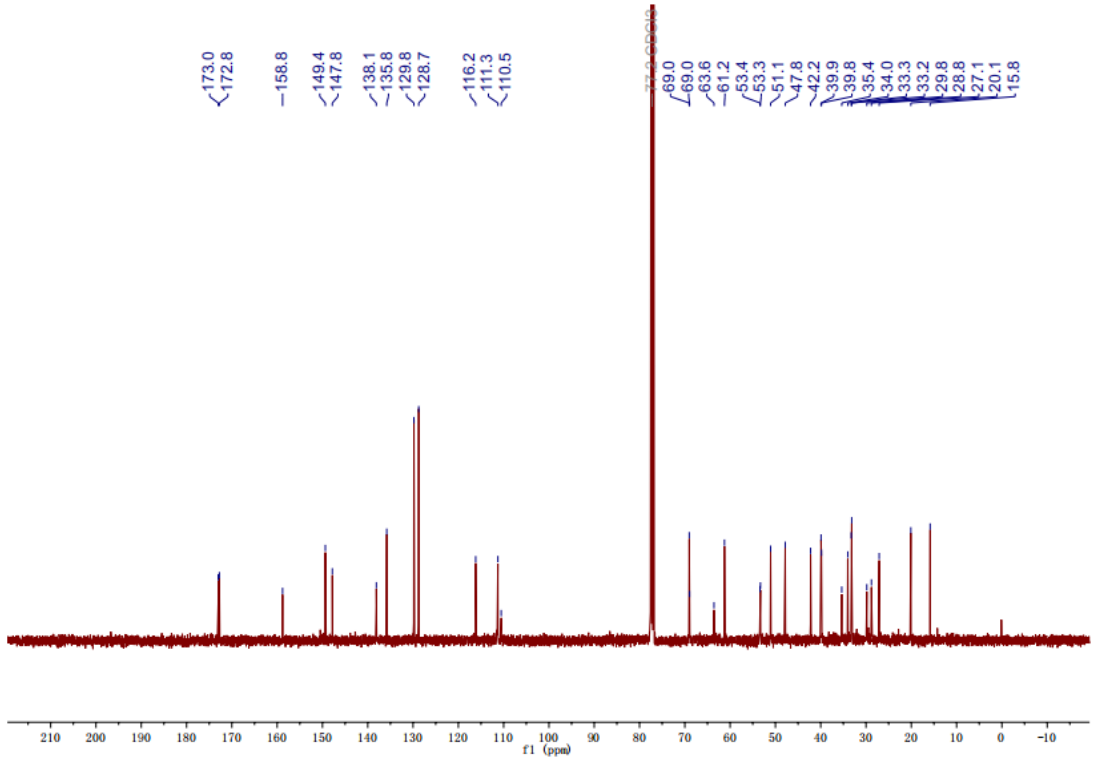

4-((4-((4-((1-(2-((1R,3R,4S)-3-(3-chloroprop-1-en-2-yl)-4-methyl-4-vinylcyclohexyl)allyl)piperidin-4-yl)methoxy)-4-oxobutanoyl)oxy)but-2-yn-1-yl)oxy)-3-(phenylsulfonyl)-1,2,5-oxadiazole 2-oxide (IVc)

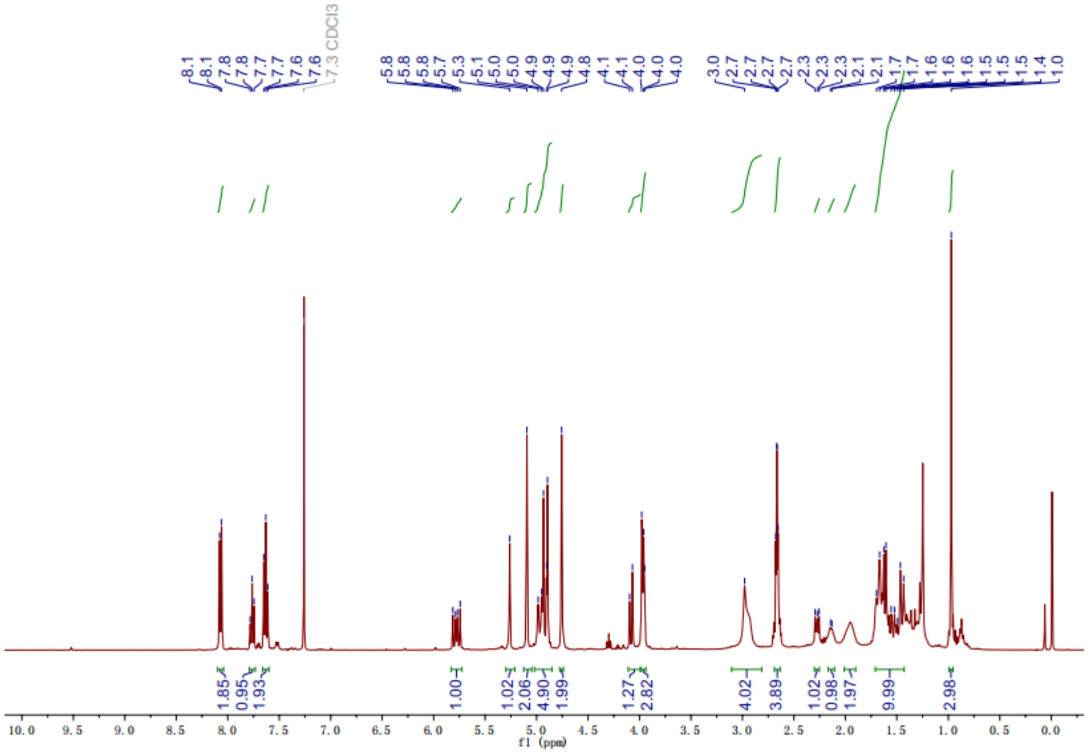

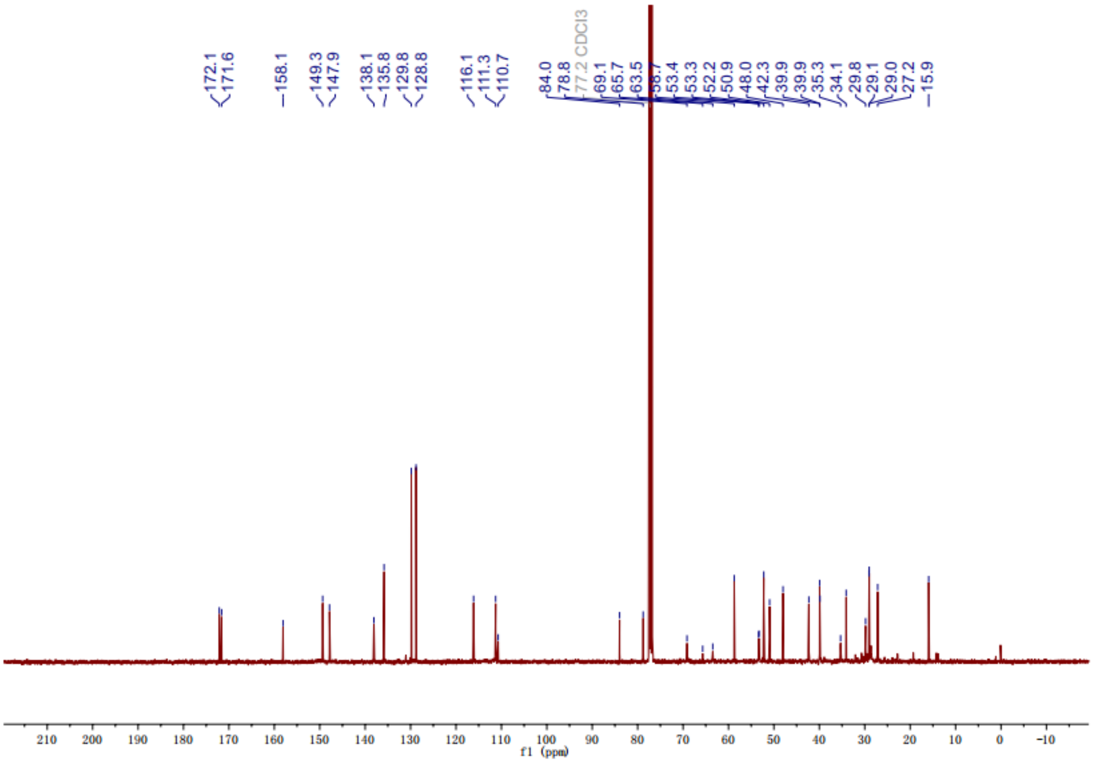

4-((4-((5-((1-(2-((1R,3R,4S)-3-(3-chloroprop-1-en-2-yl)-4-methyl-4-vinylcyclohexyl)allyl)piperidin-4-yl)methoxy)-5-oxopentanoyl)oxy)but-2-yn-1-yl)oxy)-3-(phenylsulfonyl)-1,2,5-oxadiazole 2-oxide (IVd)

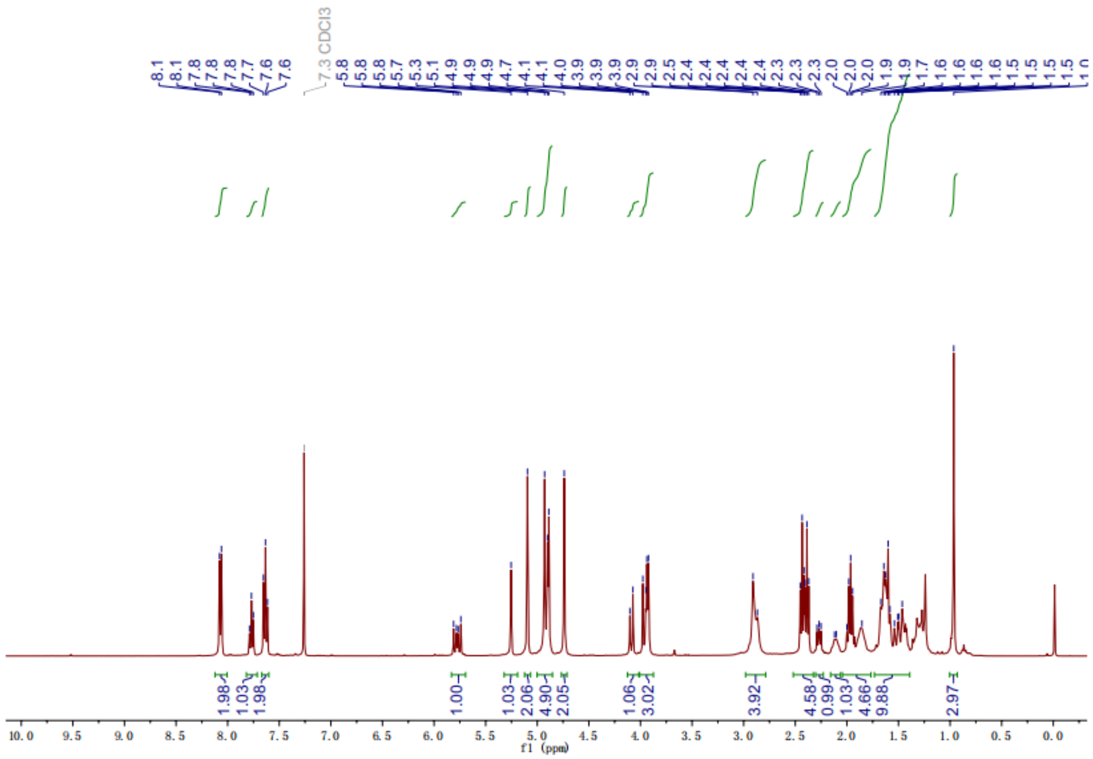

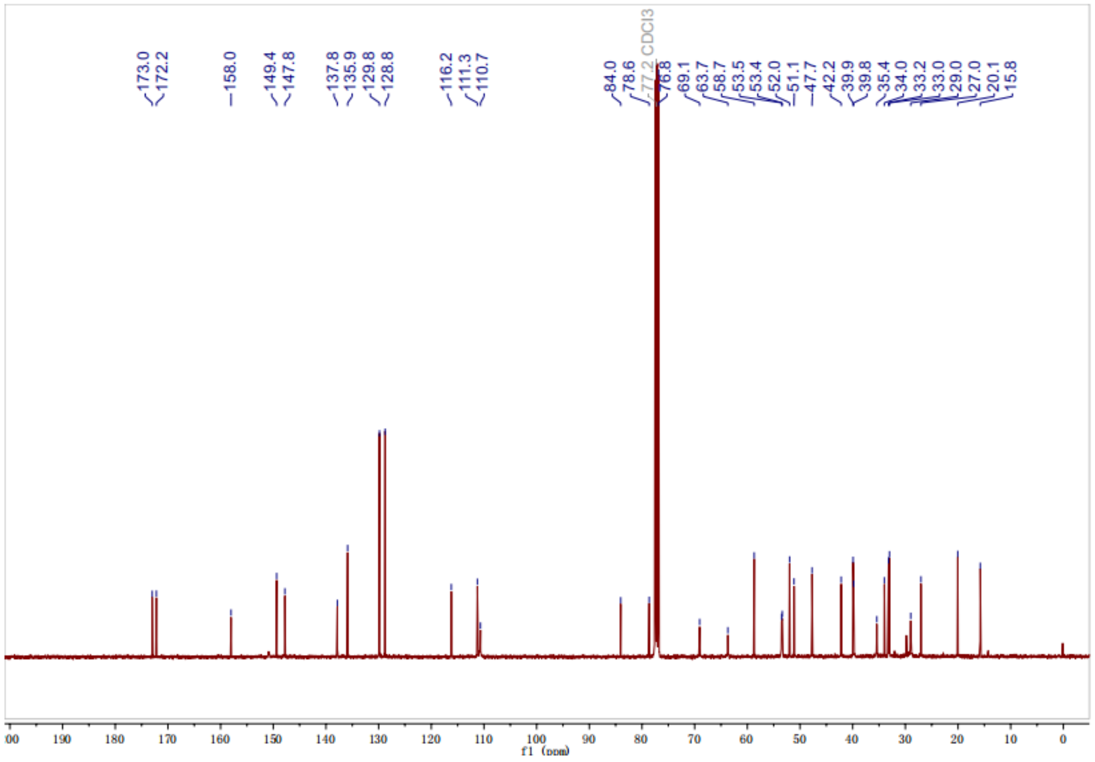

4-(2-((4-((1-(2-((1R,3R,4S)-3-(3-chloroprop-1-en-2-yl)-4-methyl-4-vinylcyclohexyl)allyl)piperidin-3-yl)methoxy)-4-oxobutanoyl)oxy)ethoxy)-3-(phenylsulfonyl)-1,2,5-oxadiazole 2-oxide (Va)

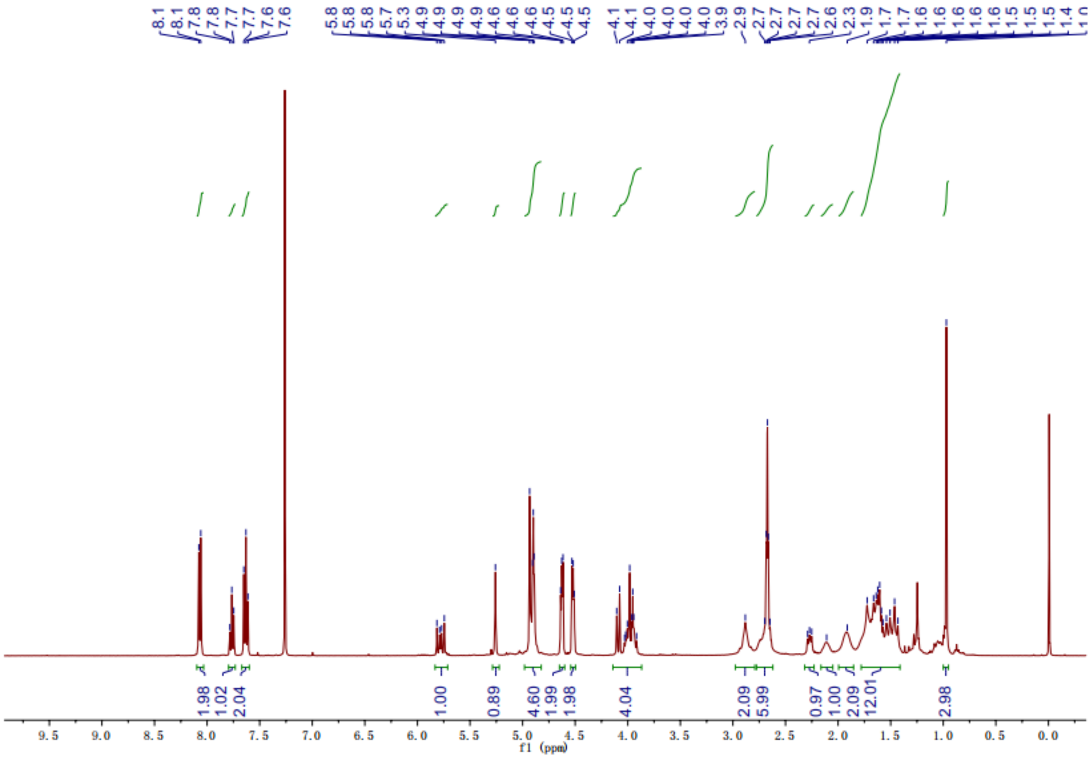

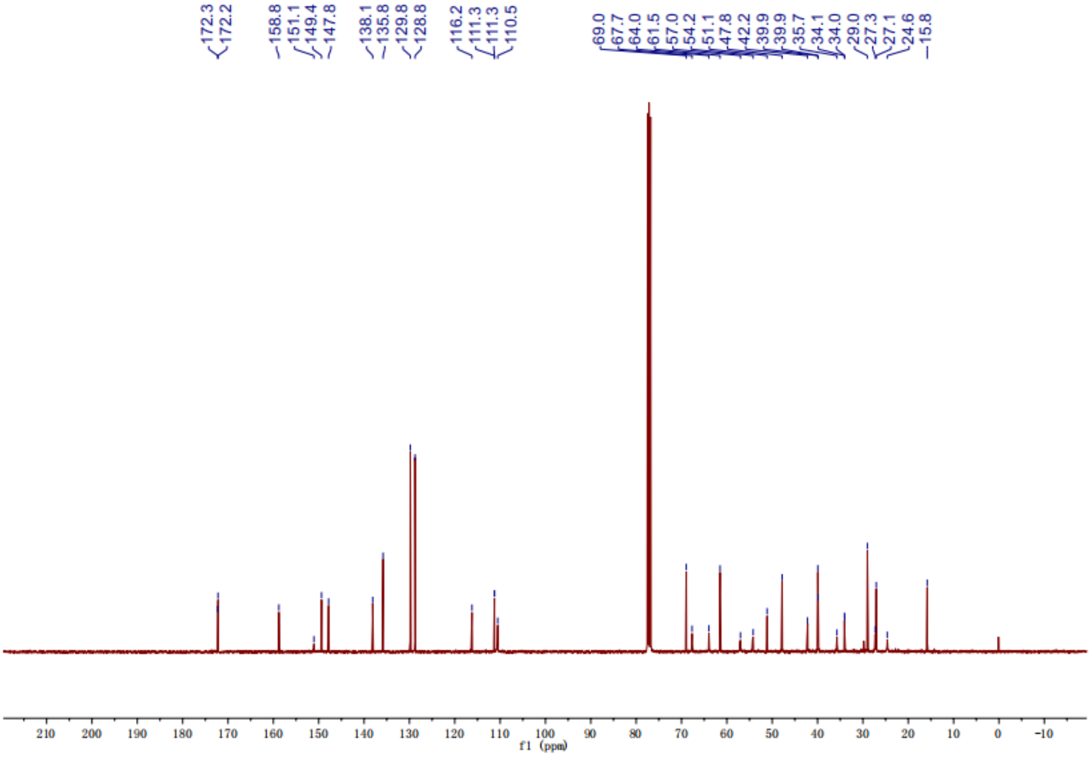

4-(2-((5-((1-(2-((1R,3R,4S)-3-(3-chloroprop-1-en-2-yl)-4-methyl-4-vinylcyclohexyl)allyl)piperidin-3-yl)methoxy)-5-oxopentanoyl)oxy)ethoxy)-3-(phenylsulfonyl)-1,2,5-oxadiazole 2-oxide (Vb)

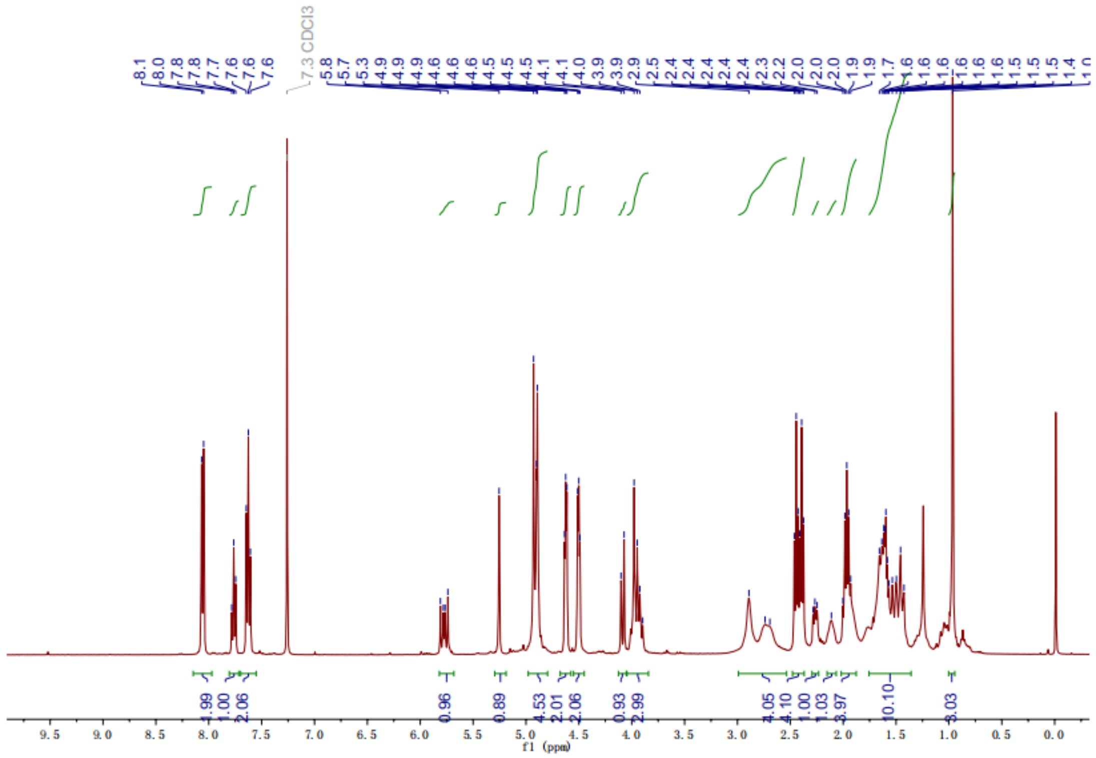

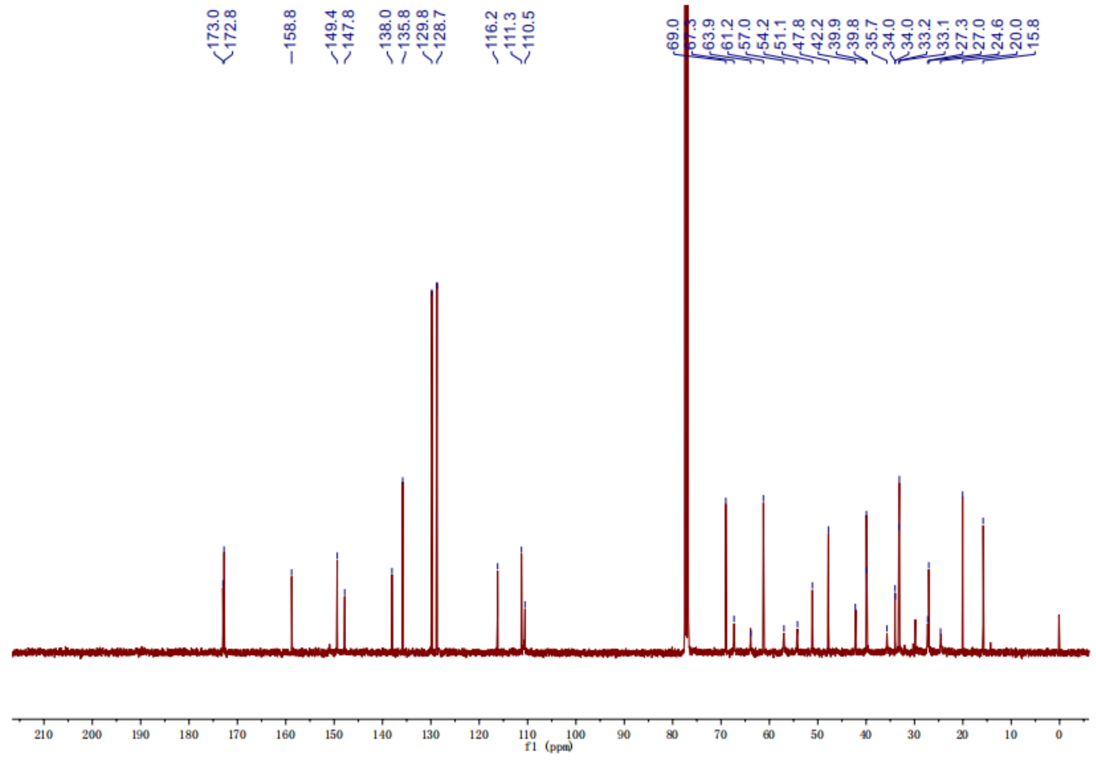

4-((4-((4-((1-(2-((1R,3R,4S)-3-(3-chloroprop-1-en-2-yl)-4-methyl-4-vinylcyclohexyl)allyl)piperidin-3-yl)methoxy)-4-oxobutanoyl)oxy)but-2-yn-1-yl)oxy)-3-(phenylsulfonyl)-1,2,5-oxadiazole 2-oxide (Vc)

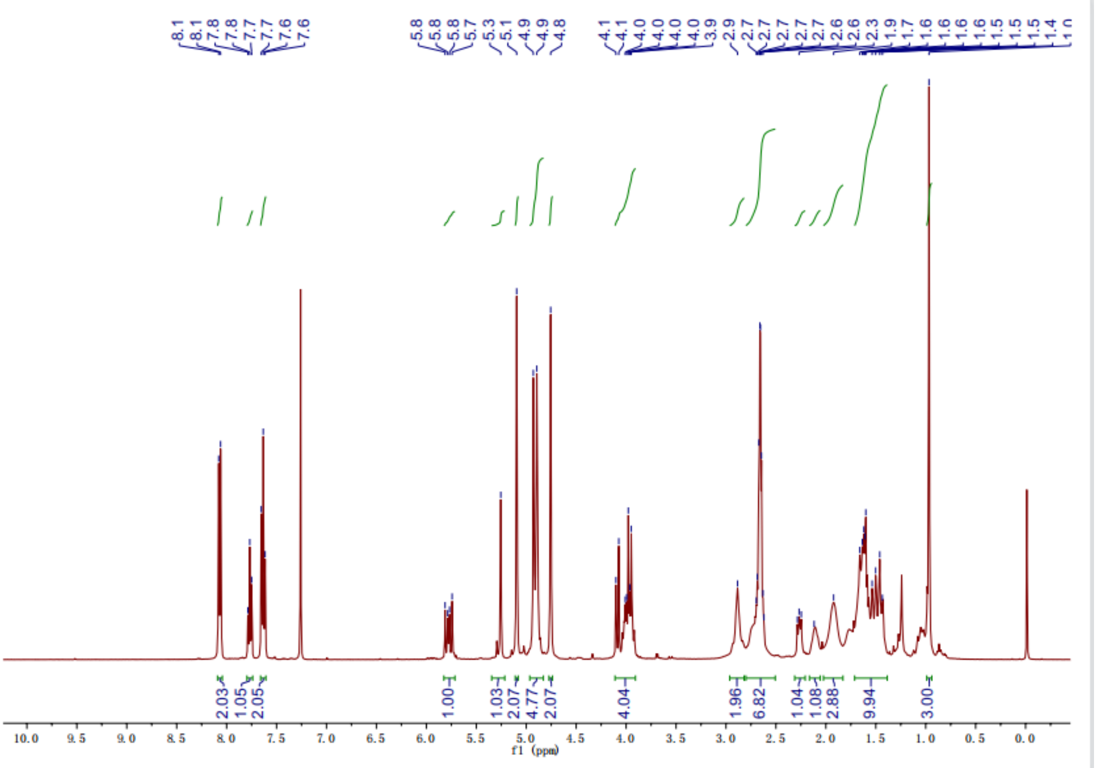

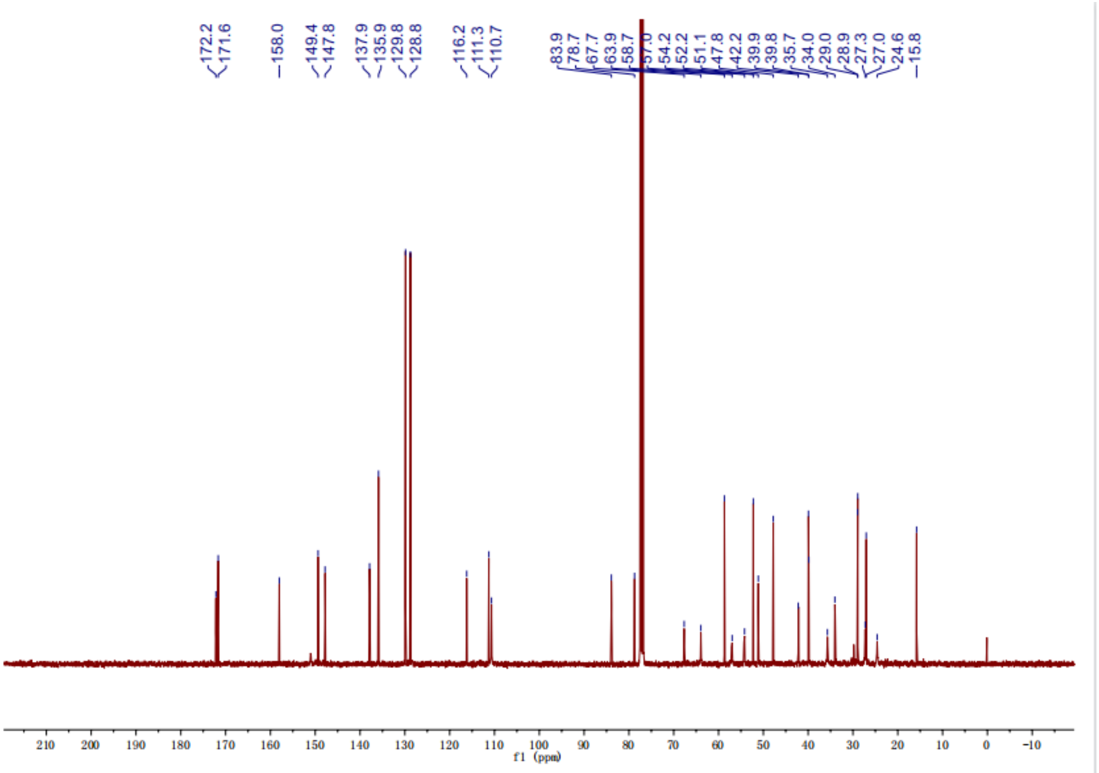

4-((4-((5-((1-(2-((1R,3R,4S)-3-(3-chloroprop-1-en-2-yl)-4-methyl-4-vinylcyclohexyl)allyl)piperidin-3-yl)methoxy)-5-oxopentanoyl)oxy)but-2-yn-1-yl)oxy)-3-(phenylsulfonyl)-1,2,5-oxadiazole 2-oxide (Vd)

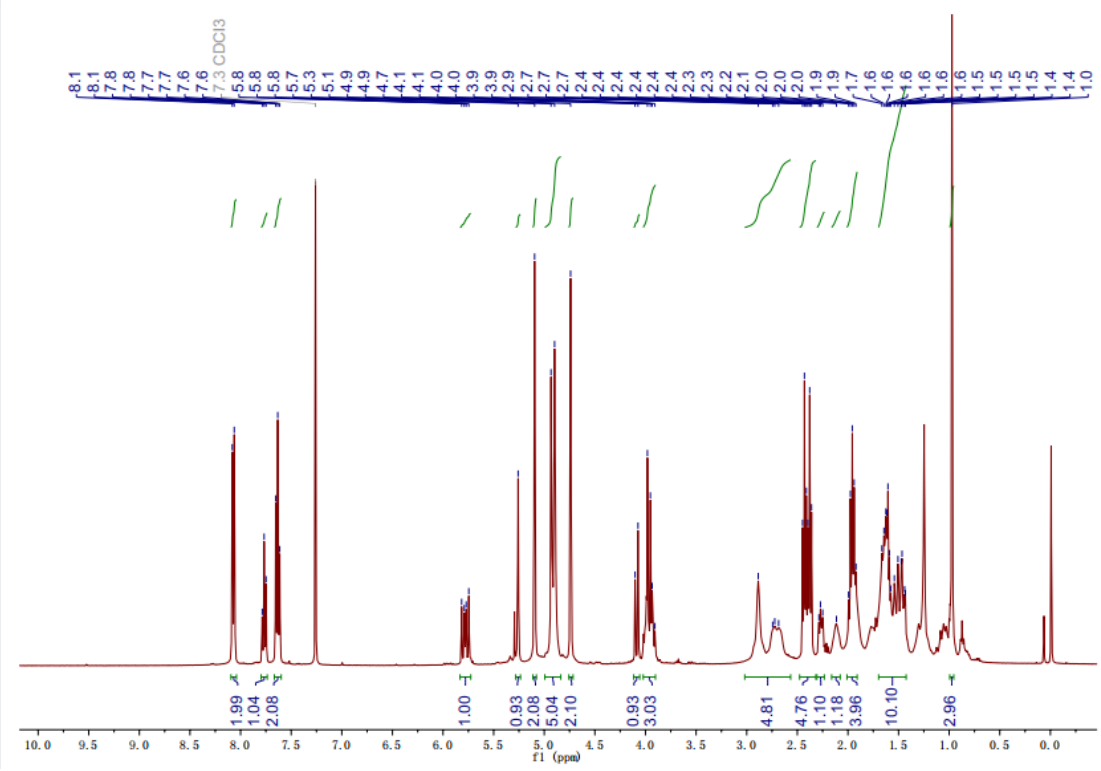

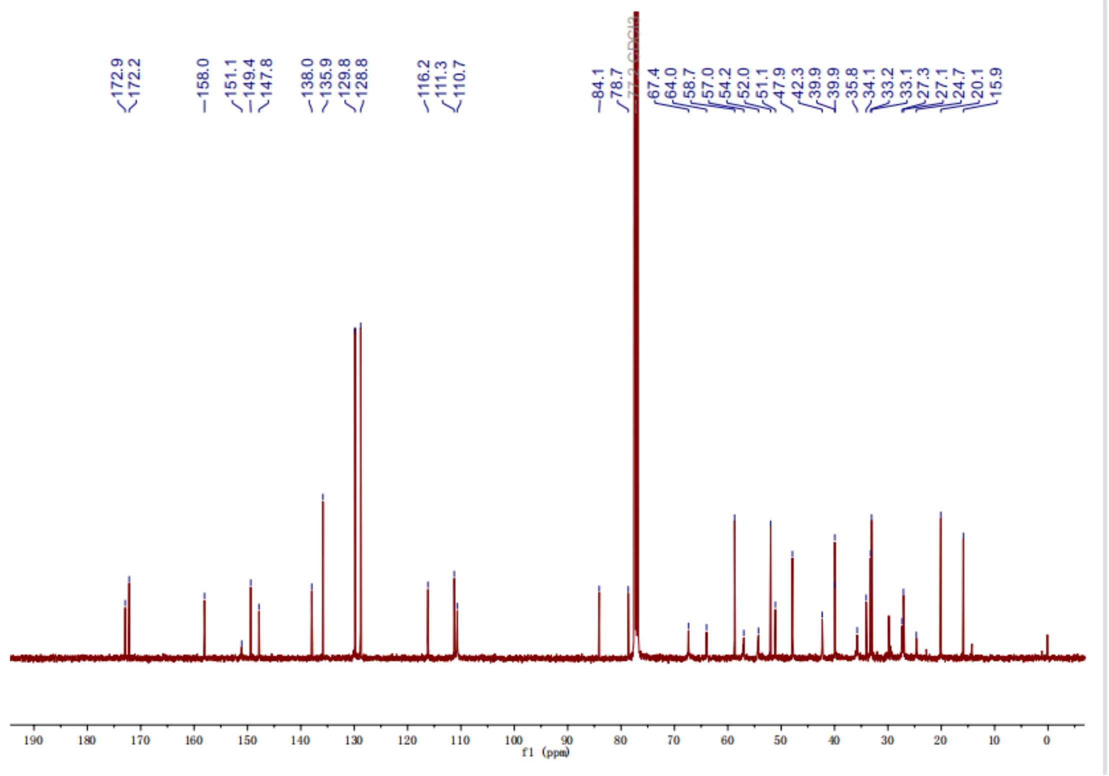

4-(2-((4-(2-(4-(2-((1R,3R,4S)-3-(3-chloroprop-1-en-2-yl)-4-methyl-4-vinylcyclohexyl)allyl)piperazin-1-yl)ethoxy)-4-oxobutanoyl)oxy)ethoxy)-3-(phenylsulfonyl)-1,2,5-oxadiazole 2-oxide (VIa)

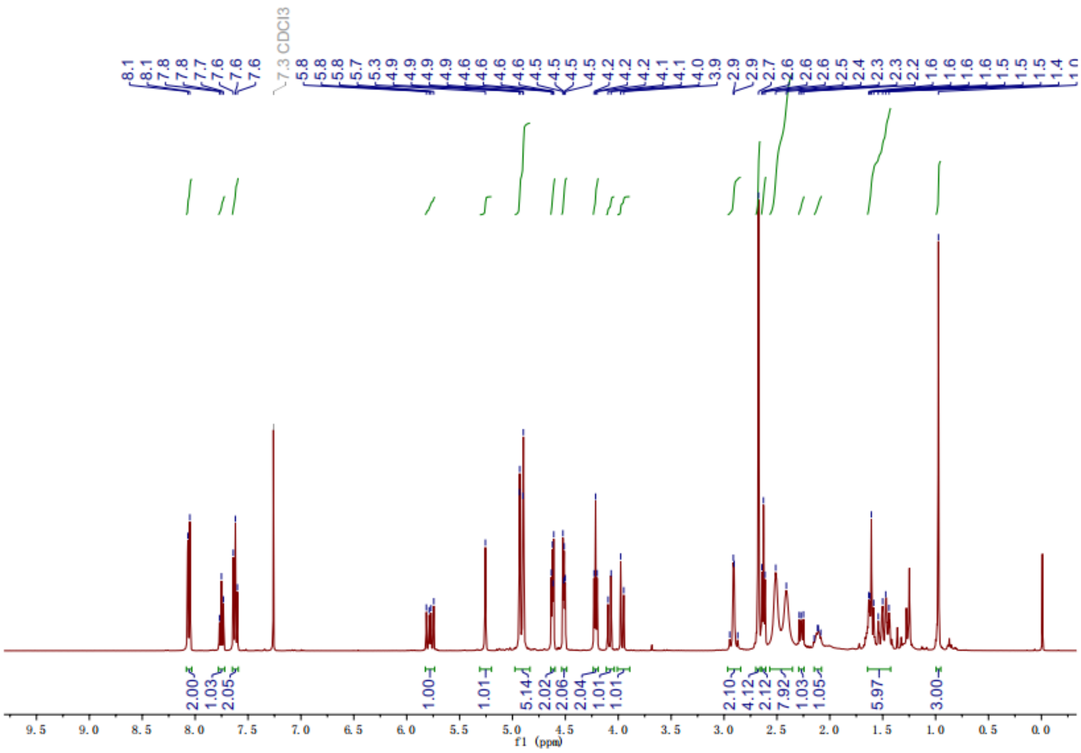

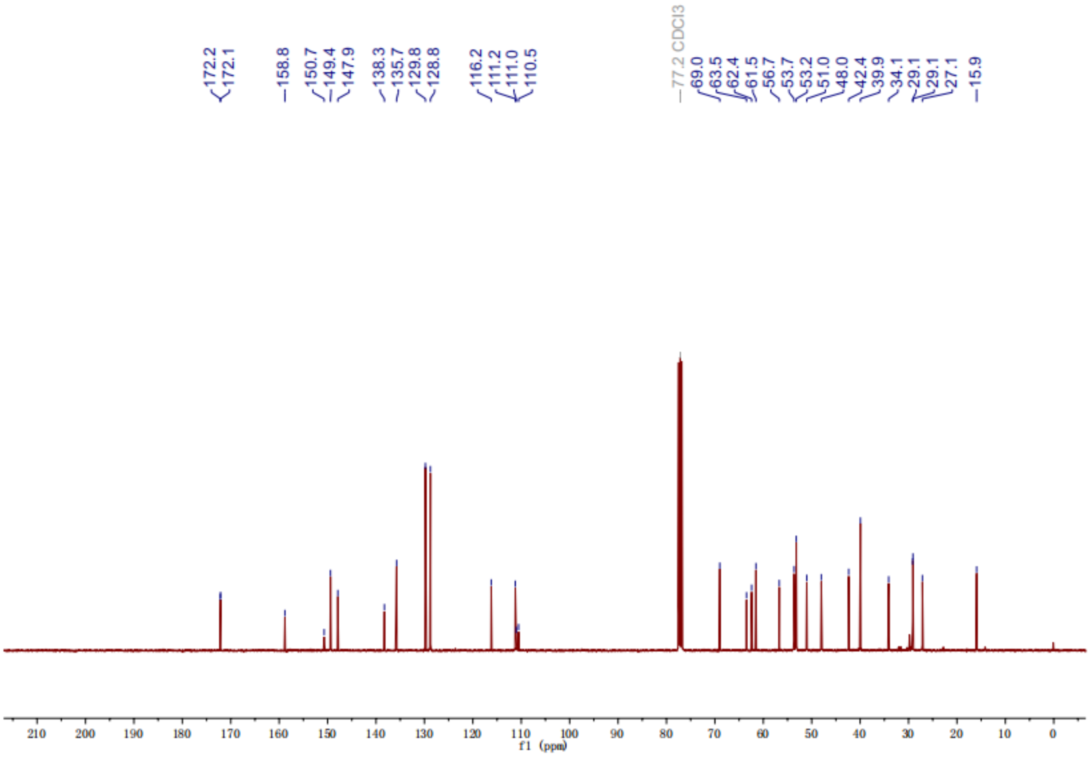

4-(2-((4-(2-(4-(2-((1R,3R,4S)-3-(3-chloroprop-1-en-2-yl)-4-methyl-4-vinylcyclohexyl)allyl)piperazin-1-yl)ethoxy)-4-oxobutanoyl)oxy)ethoxy)-3-(phenylsulfonyl)-1,2,5-oxadiazole 2-oxide (VIb)

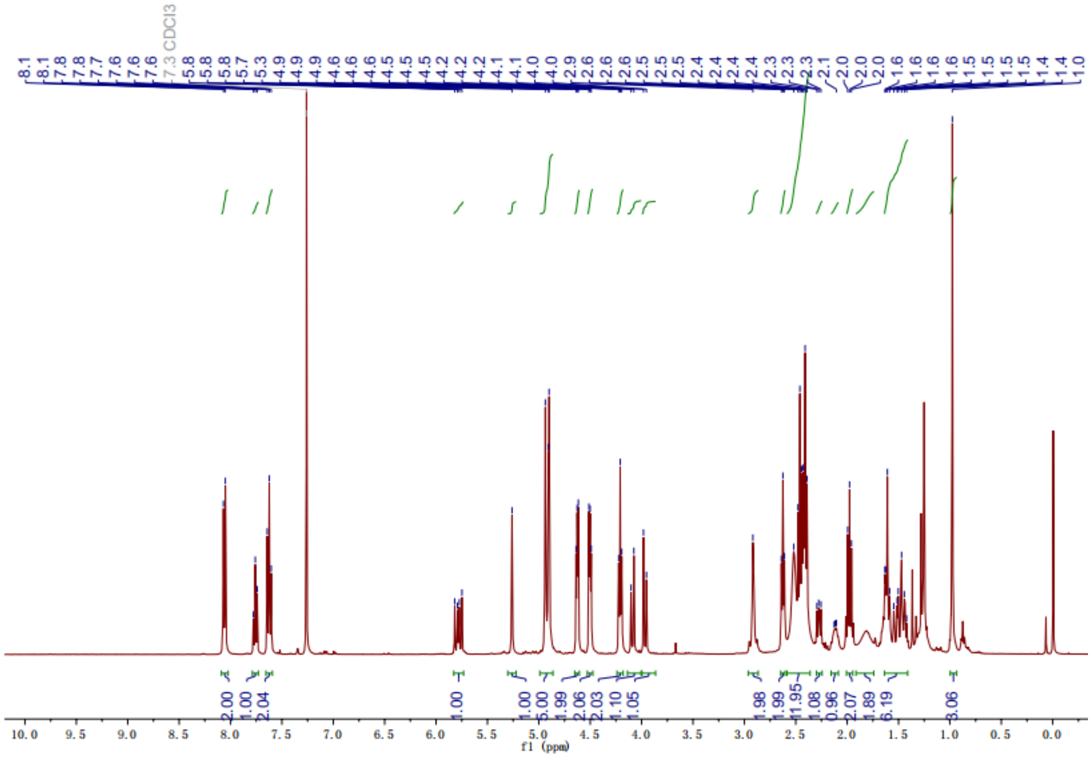

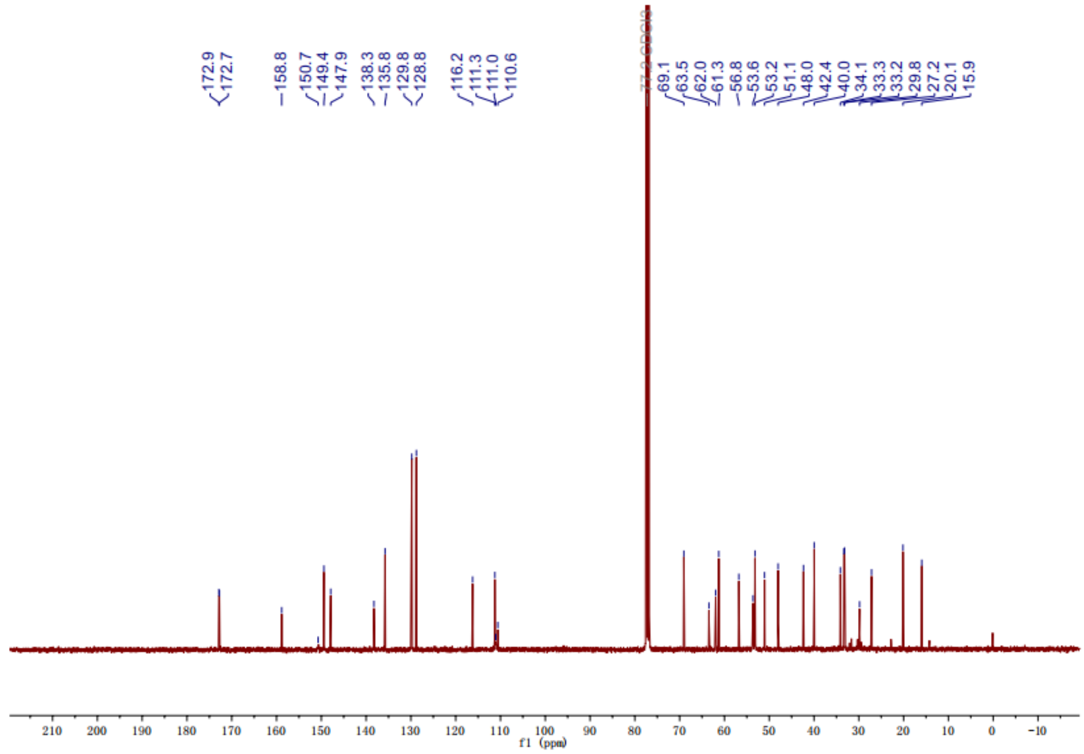

4-((4-((4-(2-(4-(2-((1R,3R,4S)-3-(3-chloroprop-1-en-2-yl)-4-methyl-4-vinylcyclohexyl)allyl)piperazin-1-yl)ethoxy)-4-oxobutanoyl)oxy)but-2-yn-1-yl)oxy)-3-(phenylsulfonyl)-1,2,5-oxadiazole 2-oxide (VIc)

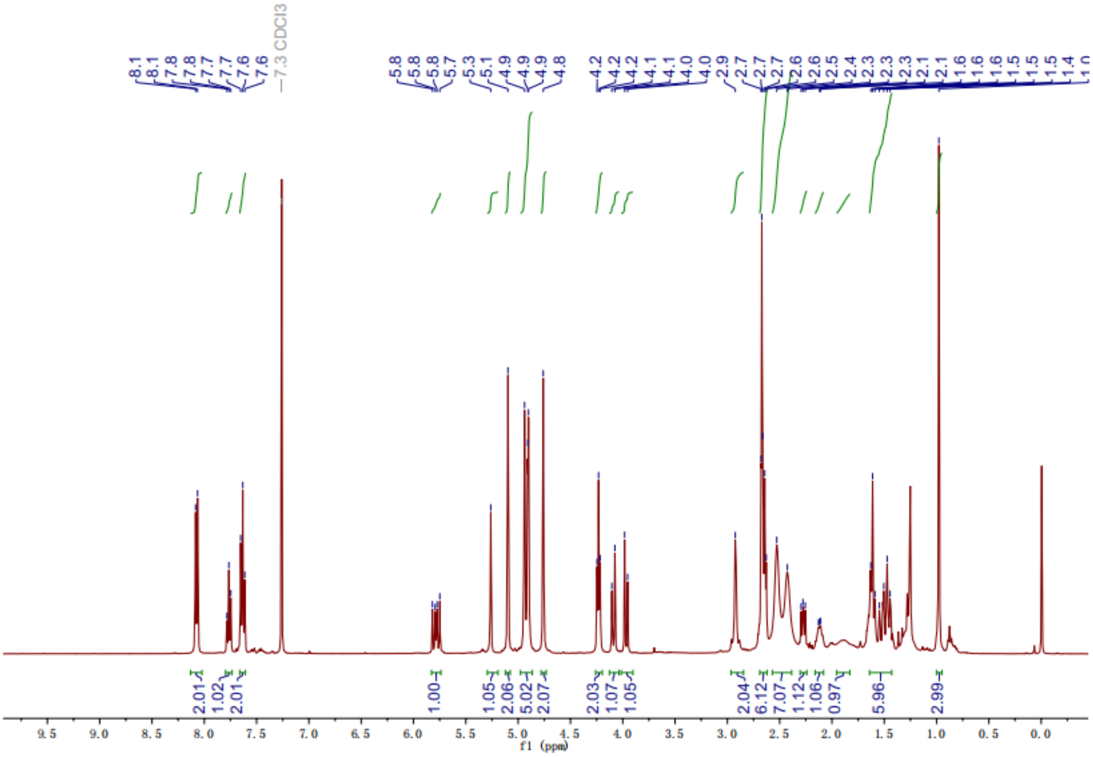

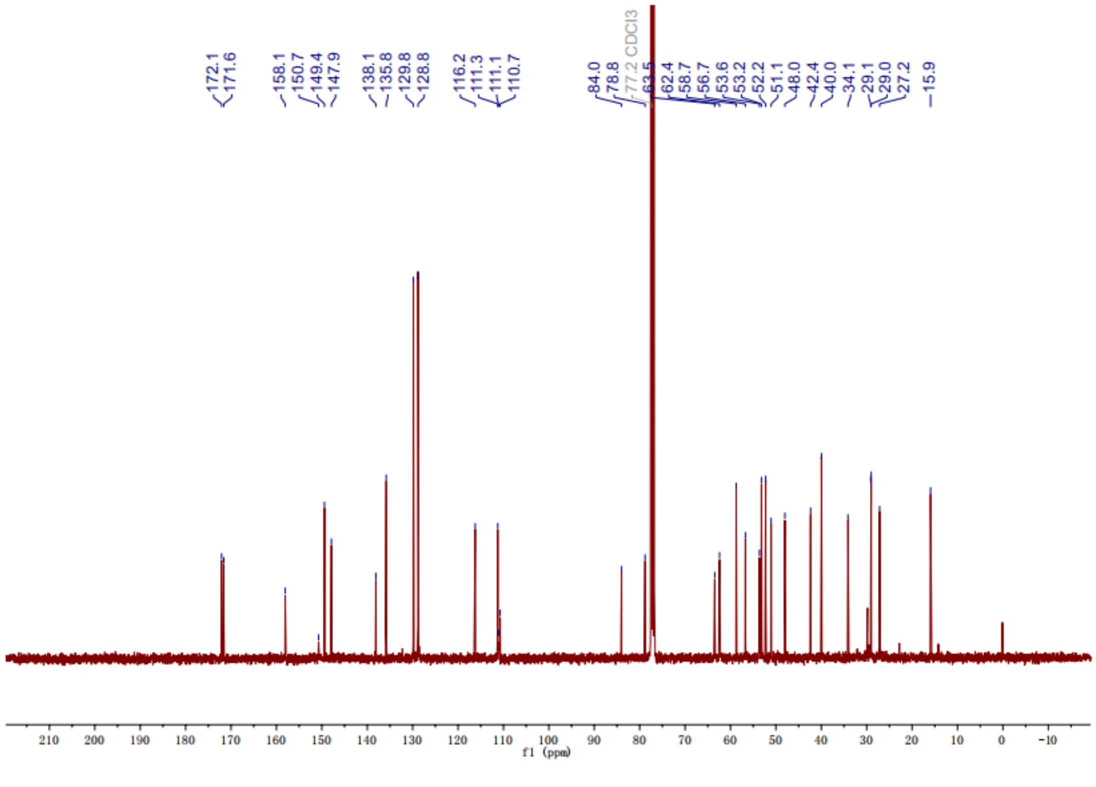

4-((4-((5-(2-(4-(2-((1R,3R,4S)-3-(3-chloroprop-1-en-2-yl)-4-methyl-4-vinylcyclohexyl)allyl)piperazin-1-yl)ethoxy)-5-oxopentanoyl)oxy)but-2-yn-1-yl)oxy)-3-(phenylsulfonyl)-1,2,5-oxadiazole 2-oxide (VId)

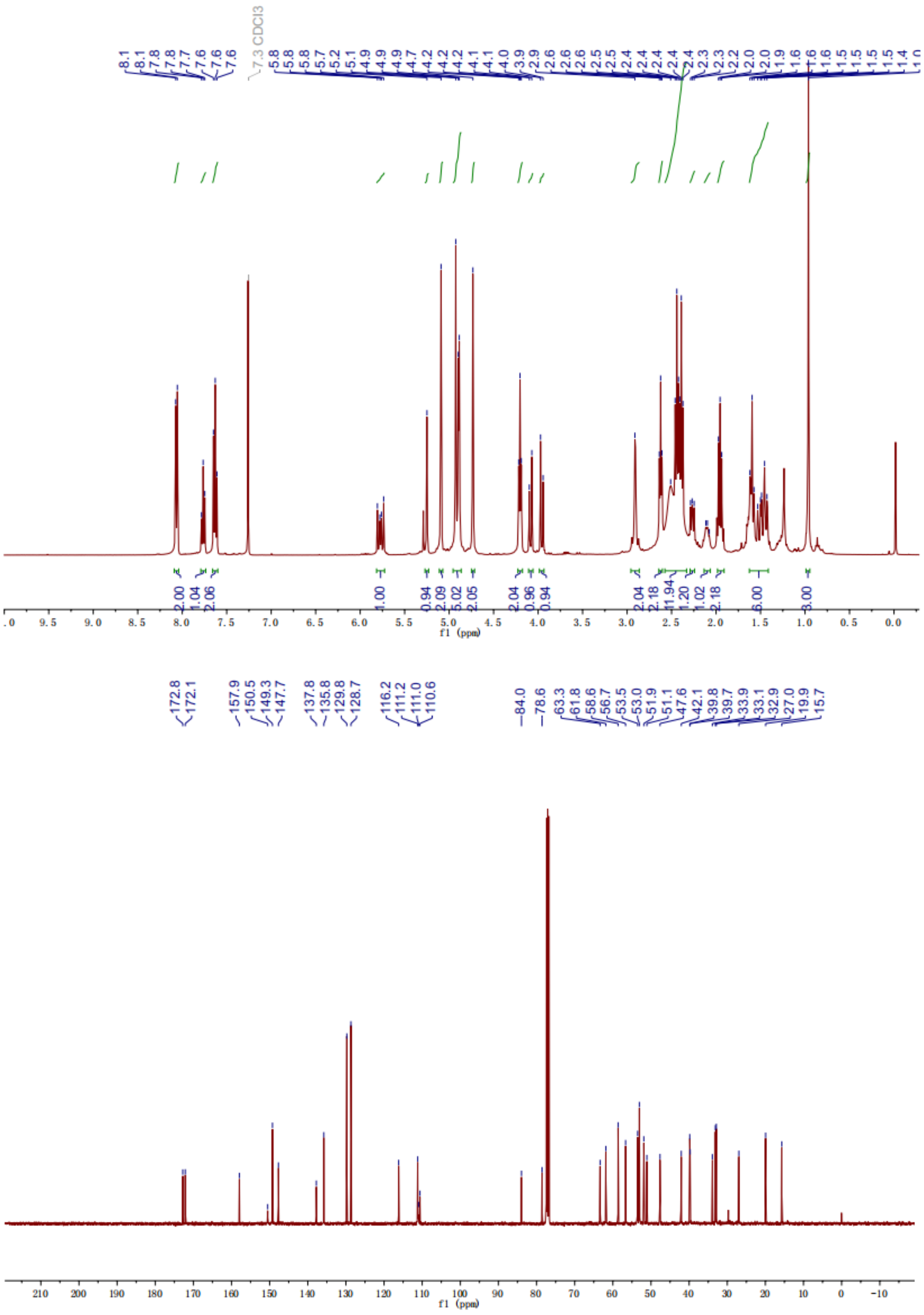

Supplement: Supplemental Material [file IENZ_A_2016734_SM3921.pdf]
